# The effect of a novel extracorporeal cytokine hemoadsorption device on IL-6 elimination in septic patients: A randomized controlled trial

**DOI:** 10.1371/journal.pone.0187015

**Published:** 2017-10-30

**Authors:** Dirk Schädler, Christine Pausch, Daniel Heise, Andreas Meier-Hellmann, Jörg Brederlau, Norbert Weiler, Gernot Marx, Christian Putensen, Claudia Spies, Achim Jörres, Michael Quintel, Christoph Engel, John A. Kellum, Martin K. Kuhlmann

**Affiliations:** 1 Department of Anesthesiology and Intensive Care Medicine, University Medical Center Schleswig-Holstein, Campus Kiel, Kiel, Germany; 2 Institute for Medical Informatics, Statistics and Epidemiology, University of Leipzig, Leipzig, Germany; 3 Centre of Anaesthesiology, Emergency and Intensive Care Medicine, University Hospital Göttingen, Göttingen, Germany; 4 Department of Anesthesiology and Intensive Care Medicine, HELIOS Klinikum, Erfurt, Germany; 5 Department of Intensive Care Medicine, Helios Hospital Berlin-Buch, Berlin, Germany; 6 Department of Intensive Care and Intermediate Care, RWTH University Hospital Aachen, Aachen, Germany; 7 Department of Anesthesiology and Intensive Care Medicine, University of Bonn, Bonn, Germany; 8 Anaesthesiology and Intensive Care Medicine, Campus Charité Mitte and Campus Charité Virchow-Klinikum, Charité - University Medicine Berlin, Berlin, Germany; 9 Department of Medicine I - Nephrology, Transplantation & Medical Intensive Care, University Witten/Herdecke, Medical Center Cologne-Merheim, Cologne, Germany; 10 Department of Critical Care Medicine, University of Pittsburgh School of Medicine, Pittsburgh, Pennsylvania, United States of America; 11 Department of Nephrology, Vivantes Klinikum im Friedrichshain, Berlin, Germany; Medizinische Universitat Graz, AUSTRIA

## Abstract

**Objective:**

We report on the effect of hemoadsorption therapy to reduce cytokines in septic patients with respiratory failure.

**Methods:**

This was a randomized, controlled, open-label, multicenter trial. Mechanically ventilated patients with severe sepsis or septic shock and acute lung injury or acute respiratory distress syndrome were eligible for study inclusion. Patients were randomly assigned to either therapy with CytoSorb hemoperfusion for 6 hours per day for up to 7 consecutive days (treatment), or no hemoperfusion (control). Primary outcome was change in normalized IL-6-serum concentrations during study day 1 and 7.

**Results:**

97 of the 100 randomized patients were analyzed. We were not able to detect differences in systemic plasma IL-6 levels between the two groups (n = 75; p = 0.15). Significant IL-6 elimination, averaging between 5 and 18% per blood pass throughout the entire treatment period was recorded. In the unadjusted analysis, 60-day-mortality was significantly higher in the treatment group (44.7%) compared to the control group (26.0%; p = 0.039). The proportion of patients receiving renal replacement therapy at the time of enrollment was higher in the treatment group (31.9%) when compared to the control group (16.3%). After adjustment for patient morbidity and baseline imbalances, no association of hemoperfusion with mortality was found (p = 0.19).

**Conclusions:**

In this patient population with predominantly septic shock and multiple organ failure, hemoadsorption removed IL-6 but this did not lead to lower plasma IL-6-levels. We did not detect statistically significant differences in the secondary outcomes multiple organ dysfunction score, ventilation time and time course of oxygenation.

## Introduction

At the early onset of sepsis [1] an overshoot of multiple pro-inflammatory mediators is often observed, caused by pathogen-associated molecular patterns (PAMPs) and damage-associated molecular patterns (DAMPs) [2]. It is known that mortality is highest when both pro- and anti-inflammatory cytokine levels are highest [3]. It has been suggested that blocking this overproduction would stop sepsis and improve patient outcomes. Therapeutic monoclonal antibodies targeting specific cytokines such as tumor necrosis factor-α (anti-TNF-α) have demonstrated mortality benefit in animal sepsis models and small scale human sepsis studies [4,5] but have not succeeded in large scale pivotal trials in septic patients. There are no currently approved specific therapies to treat sepsis, following the withdrawal of recombinant activated protein C (Xigris; Eli Lilly, USA) from the market in 2011 following the results of the PROWESS-SHOCK trial [6,7].

Another approach to decrease cytokine overproduction is extracorporeal blood purification. De Vriese et al. observed a significant short-term decrease of pro- and anti-inflammatory cytokines one hour after the start of continuous venovenous hemofiltration in patients with septic shock and acute kidney injury [4]. However, moderate sized trials of standard [5] or high-volume hemofiltration [6] did not show an improvement of survival and did not influence plasma cytokine levels measured in a small subgroup [5]. In a recent meta-analysis of the various forms of blood purification, only plasma exchange, and hemoadsorption appeared to be potentially effective for treatment of sepsis [7].

Hemoperfusion using polymyxin b is currently used in the treatment of sepsis in Japan [8–12]. The EUPHAS study, a randomized controlled multi-center trial investigating the effect of polymyxin b hemoperfusion in patients with abdominal septic shock showed promising results as vasopressor requirements in the hemoperfusion group was significantly lower, and the P-F-ratio (partial pressure of oxygen divided by the inspired fraction of oxygen) was significantly higher compared to controls. In a post-hoc analysis, hemoperfusion was associated with improved survival [13]. However, in a large randomized controlled multi-center trial in France, Payen et al. did not detect a significant effect of hemoperfusion on mortality or improvement of organ dysfunction [14].

CytoSorb (Cytosorbents, Corporation, New Jersey, USA) is a hemoadsorption device containing hemocompatible, porous polymeric beads capable of removing cytokines and other mid-molecular weight toxins from blood by size exclusion and surface adsorption. The polymer is both highly adsorptive and biocompatible and has been approved in the European Union. Multiple pre-clinical studies using animal models of sepsis have demonstrated reductions in various circulating cytokines and chemokines, reduced organ injury, and improved survival [15–18]. Kellum et al. detected reduction rates in circulating IL-6 in the range of 56% to 78% in an experimental endotoxemia setting [15]. A feasibility study in brain-dead humans confirmed reductions in cytokines without any adverse effects [19]. In that study, the authors reported IL-6 extraction rates ranging from 27.5% to 30.2%. Thus, in this “first-in-septic patients” phase 2 randomized, controlled clinical trial, we sought to investigate the efficacy of cytokine reduction using the change in plasma interleukin (IL)-6 concentrations over time as a primary outcome. Although the trial was neither designed nor powered to evaluate outcome, we also evaluated organ function parameters as well as all-cause mortality.

## Methods

This open label, randomized, controlled multi-center trial was conducted in 10 German study sites during April 2008 (first patient in) and June 2011 (last patient out). The study was approved by the ethics committee of Göttingen University and registered at clinicaltrials.gov (NCT00559130). Mechanically ventilated patients with severe sepsis or septic shock according to the American College of Chest Physicians and Society of Critical Care Medicine criteria [20] in the setting of acute lung injury (ALI) or acute respiratory distress syndrome (ARDS) according to the American-European consensus definition [21] established within the last 72 hours were eligible for study inclusion (please see S3 File for all inclusion and exclusion criteria). Before study inclusion, written informed consent was obtained from all patients. If a patient was not able to give consent the legal representative was asked to consent. In the latter case patients were asked to give study consent after being capable to understand the study’s nature and importance.

Patients were randomly assigned either to be treated with standard of care therapy and CytoSorb hemoperfusion for 6 hours per day, up to seven consecutive days (treatment group) or to standard of care therapy alone (control group). In the treatment group, the CytoSorb cartridge was either used alone in hemoperfusion mode or, if renal replacement therapy was clinically indicated, inserted proximally into a conventional continuous veno-venous hemofiltration (CVVH) or continuous veno-venous hemodiafiltration (CVVHDF) circuit. Target blood flow rates were 200–250 mL/min, where blood flow enters the bottom of a vertically-oriented cartridge, and exits through the top. Anti-coagulation using either regional citrate anticoagulation per hospital protocol (target postfilter ionized calcium < 0.4 mmol/l), or systemic heparin anti-coagulation with a target partial thromboplastin time (PTT) of 60–80, or an activated clotting time (ACT) of 180–210, with confirmation of adequate anti-coagulation before initiation of treatment, was recommended. Administration of antibiotics was recommended after CytoSorb treatment where possible. Hemoperfusion was stopped earlier if patients were successfully weaned from mechanical ventilation.

Randomization was performed in a 1:1 fashion with a block length of 4, using sealed envelopes prepared by the clinical trial contract research organization, MedPass International (Paris, France). Patients were enrolled and randomized by the local study site investigators. Because of observed and suspected irregularities with regard to the use of the sealed randomization envelopes (envelopes were not security envelopes as originally planned and could be seen through and in a random audit by MedPass, two envelopes were opened without patient enrollment) during a time period of 11 months pertaining the randomization of n = 32 patients, the randomization method was switched to an electronic system (using a block length of 6) following a recommendation of the independent data monitoring and safety board.

The primary endpoint of the study was normalized IL-6 serum concentrations shortly before the initiation of treatment (T_0_) and shortly after the end of 6 hours of treatment (T_360_) from Day 1 to Day 7. Normalized values were derived as the observed value divided by the individual baseline measurement at Day 1 before device application (D1T_0_). Secondary endpoints were ventilation time, normalized levels of chemokine ligand 2 (CCL2), CCL3, Interferon- γ (IFN-γ), Il-1β, IL-1ra, IL-4, IL-8, Il-10, tumor necrosis factor alpha (TNF-α), vascular endothelial growth factor (VEGF), CD4^+^ T cell activation (Immuknow assay, ViraCor-IBT Laboratories, Missouri, USA), 28-day all-cause mortality, oxygenation index, P/F-ratio and multiple organ dysfunction score (MODS). MODS summarizes the organ function of the cardiovascular system (pressure adjusted heart rate), the respiratory system (P/F-ratio), the renal system (serum creatinine concentration), the hepatic system (serum bilirubin concentration), the hematologic system (platelet count) and the central nervous system (Glasgow Coma Scale) [22]. We prospectively defined the following exploratory endpoints: IL-6 elimination by the study device, independent risk factors for death and IL-6-plasma levels, and an analysis of the time course of vital parameters and laboratory values.

IL-6 elimination by the study device was analyzed on study Day 2, where five additional blood samples were drawn during hemoperfusion for IL-6 kinetic analyses (T_0_, start of treatment; T_15_, 15 minutes after start of treatment; T_60_, 60 minutes after start of treatment; T_180_, 180 minutes after start of treatment; T_360_, 360 minutes after start of treatment). IL-6 elimination was calculated using the following formula:

$$IL-6\;elimination\;=\;100\times \frac{\left({BI}_{a}-{BO}_{v}\right)}{{BI}_{a}}$$

where BI_a_ = arterial (inlet) blood IL-6 level, BO_v_ = venous (outlet) blood IL-6 level.

### Statistical analysis

Previously, the device was only applied in selected cases. Therefore, a power analysis could not be performed. A sample size of 100 patients was considered adequate to collect complete data sets including seven days of study treatment on 30 subjects in each group.

The confirmatory analyses followed the intention-to-treat principle based on all patients who were randomized and provided informed consent. Mixed models were used to analyze the primary endpoint and the secondary cytokine endpoints. They allow to assess the treatment effect taking the repeated measurements structure of the data into account, i.e. the fact that multiple correlated measurements per patients exist. Patients are included as ‘random effects’ (including random slopes concerning variable day), day (modeled as a polynomial of degree three in order to account for the possible non-linearity of daily changes) and time points as ‘fixed effects’. Normalized values were log-transformed to obtain approximately normally distributed data. Secondary and other exploratory endpoints were analyzed using the chi-squared test and the Mann-Whitney-U test, as appropriate. Overall survival was estimated using the Kaplan-Meier method and compared between groups using the log-rank test. To adjust for baseline imbalances and to adjust for variables known to be predictive for mortality, multivariate regression models were used for selected endpoints. All reported P values are 2-sided. P values ≤ 0.05 were considered statistically significant. All statistical analyses were performed using R [23].

## Results

**Fig 1:**
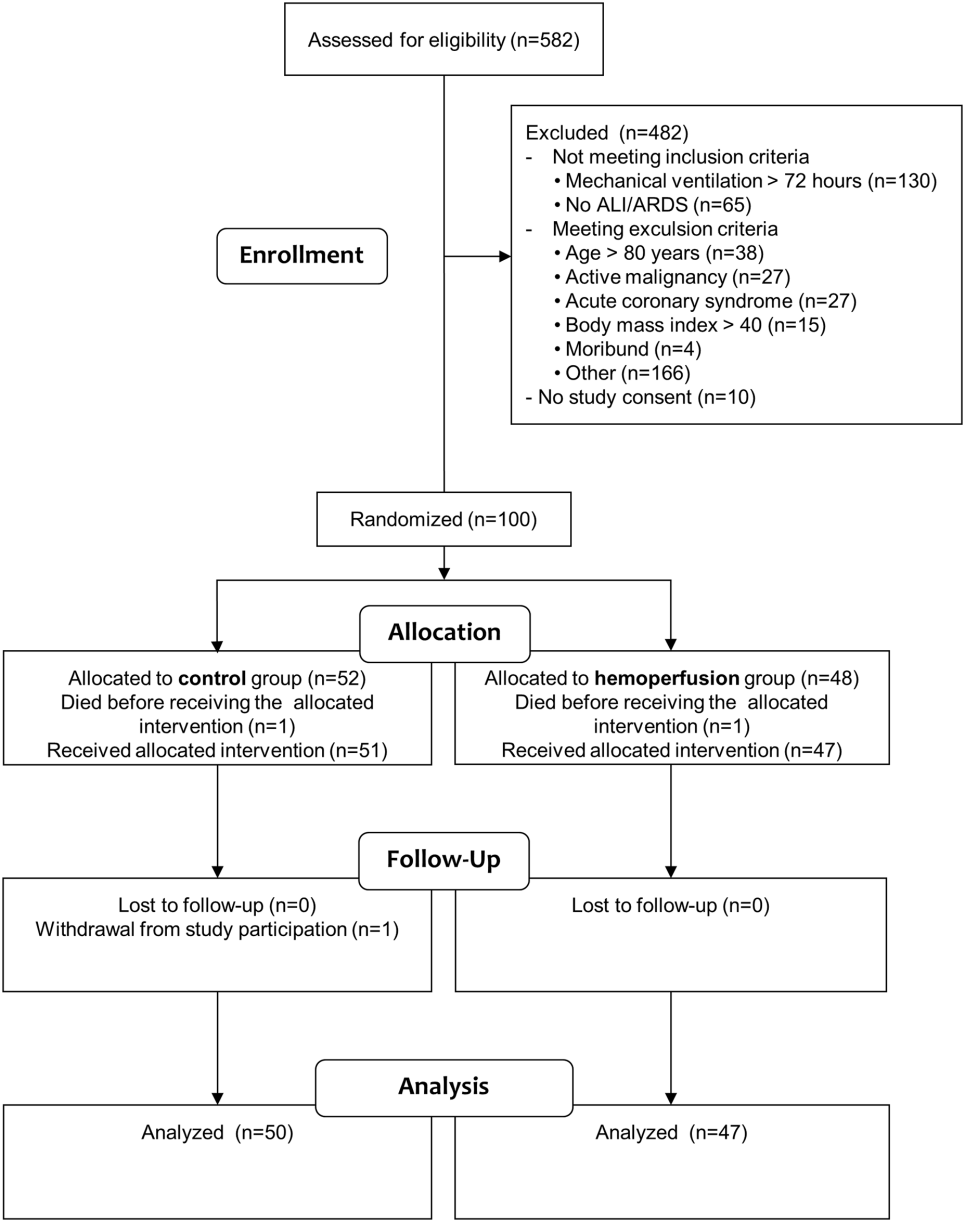
Flowchart of patients.

A total of 582 patients were screened for study enrollment. The trial ended as planned after the enrollment of 100 patients. Two patients who died before receiving their allocated study intervention and one patient who withdrew study consent after regaining consciousness were excluded from all analyses. The remaining 97 patients were included in the analysis (full analysis set) (Fig 1).

Baseline characteristics on the 97 patients are provided in Table 1. The majority of patients had septic shock (96% control, 91.5% treatment) and ARDS (64% control, 70.2% treatment). The proportion of patients receiving renal replacement therapy at the time of enrollment into the trial was higher in the treatment group (31.9%) when compared to the control group (16.3%). 25 patients in the treatment group and 12 patients in the control group showed at least one serious adverse event. One serious adverse event in the treatment group (drop in platelet count) was classified to be probably connected with the study device. A complete list of adverse events and serious adverse events is given in Table 2. No interruption during the treatment period was recorded due to technical failure of the hemoperfusion device. Hemoadsorption was interrupted three times in three patients during the study due to adverse events.

**Table 1:** Demographic data and baseline characteristics of all studied patients.

Variable	Treatment group (n = 47)	Control group (n = 50)
Age [years]	66.0 [55–73]	65 [56.5–71]
Male gender, no. (%)	35 (74.5%)	35 (70%)
Weight [kg]	77.8±13.7	84.5±17.9
Height [cm]	171.9±8.3	171.8±8.3
Body mass index [kg/m^2^]	26.4±4.6	28.5±5.2
APACHE II score*	24.6±5.2	23.8±5.7
Renal replacement therapy, no. (%)	15 (31.9%)	8 (16.3%)
Diabetes mellitus, no. (%)	17 (36.2)	19 (38.8%)
White blood cell count (1/μl)	13.4 [8.6–18.1]	16.2 [12.4–21.3]
Creatinine (mg/dl)	1.7 [0.9–2.1]	1.9 [1.1–3.0]
Albumin (g/dl)	1.9 [1.5–2.4]	2.1 [1.8–2.2]
Total protein (g/l)	4.5 [4.0–5.1]	4.7 [4.3–5.1]
**Lung injury category**		
Sepsis		
Primary, no. (%)	17 (36.2%)	13 (26.0%)
Secondary, no. (%)	30 (63.8%)	37 (74.0%)
Trauma		
Primary, no. (%)	3 (6.7%)	3 (6.1%)
Secondary, no. (%)	1 (2.2%)	0 (0.0%)
Aspiration		
Primary, no. (%)	3 (6.5%)	6 (12.2%)
Secondary, no. (%)	0 (0.0%)	3 (6.1%)
Multiple transfusion		
Primary, no. (%)	0 (0.0%)	3 (6.2%)
Secondary, no. (%)	5 (11.1%)	2 (4.2%)
Pneumonia		
Primary, no. (%)	27 (57.4%)	23 (46.0%)
Secondary, no. (%)	10 (21.3%)	20 (40.0%)
Other		
Primary, no. (%)	5 (11.6%)	10 (20.8%)
Secondary, no. (%)	8 (18.6%)	5 (10.4%)
Other comorbid conditions, no. (%)	34 (72.3%)	41 (83.7%)

Values are given as median and interquartile ranges or as mean ± standard deviation unless otherwise noted.

* Missing subscores on the Acute Physiologic and Chronic Health Evaluation (APACHE II Score) were counted as 0.

**Table 2:** Number of adverse and serious adverse events of all studied patients.

Variable	Treatment group (n = 47)	Control group (n = 50)
**Adverse events**	53	48
Possibly in connection with study device	7	n/a
Probably in connection with study device	1	n/a
**Serious adverse events**	26	15
Possibly in connection with study device	4	n/a
Probably in connection with study device	1	n/a
Reduction in platelet count	1	n/a
Patients with at least one adverse event	30	25
Patients with at least one serious adverse event	25	12

During the treatment period (the first seven study days) six patients (12.8%) died in the treatment group and five (10.0%) in the control group. In the unadjusted analysis, there was a trend to a higher 28-day-mortality in the treatment group (36.2%) when compared to the control group (18.0%, p = 0.073). In the treatment group 60-day-mortality was significantly higher (44.7%) compared to the control group (26.0%; p = 0.039). A multivariate Cox regression model on overall survival adjusting for age, male sex, Acute Physiologic and Chronic Health Evaluation II score (APACHE-II) and renal replacement therapy revealed no significant association of the study intervention and mortality (p = 0.192, please see Table 3).

**Table 3:** Univariate and multivariate cox regression models on overall survival of all studied patients (n = 97).

Univariate	Estimate	Hazard ratio	95% CI	P Value
Treatment group	0.732	2.080	[1.022, 4.232]	**0.043**
Age	0.042	1.043	[1.010, 1.077]	**0.010**
Male sex	0.170	1.185	[0.534, 2.627]	0.676
APACHE II score	0.119	1.126	[1.052, 1.206]	**0.001**
Renal replacement therapy	1.157	3.180	[1.589, 6.393]	**0.001**
**Multivariate**				
Treatment group	0.513	1.670	[0.773, 3.610]	0.192
Age	0.031	1.032	[0.991, 1.074]	0.128
Male sex	0.110	1.116	[0.484, 2.574]	0.797
APACHE II score	0.062	1.064	[0.970, 1.167]	0.187
Renal replacement therapy	0.442	1.556	[0.676, 3.581]	0.299

Definition of abbreviation: APACHE, Acute Physiologic and Chronic Health Evaluation; CI, Confidence interval.

In 22 patients, no valid measurement of the primary end-point was available due to technical problems with the cytokine blood probe management. These patients were excluded from all further analyses. Baseline demographic data of the remaining 75 patients are shown in S1–S3 Tables. The majority of patients had septic shock (97.4% control, 91.7% treatment) and ARDS (61.5% control, 72.2% treatment). The proportion of patients receiving renal replacement therapy was higher in the treatment group (38.9%) when compared to the control group (17.9%). Cytokine measurements at baseline are given in Table 4. All 36 patients randomized into the treatment group received hemoperfusion. Of these, 26 patients were treated on each of the planned seven days of treatment. During five study days the study device was not applied as recommended by the study protocol. No patient in the control group was treated with the study device.

**Table 4:** Baseline cytokines measurements in pg/ml of patients with available primary endpoint.

Variable	Treatment group (n = 36)	No. treatment group	Control group (n = 39)	No. control group
IL-6	552 [162–874]	33	590 [125–2147]	39
TNF-α	¤	32	¤	36
IL-1β	#	32	#	36
IL-10	17.6 [17.6–21.6]	32	17.6 [17.6–21.9]	36
CCL2 (MCP-1)	411 [220–1350]	32	371 [215–1024]	36
CCL3 (MIP-1α)	116 [90–166]	32	127 [90–247]	36
IFN-γ	§	31	§	36
IL-1ra	8450 [3774–16112]	32	15265 [4719–22000]	36
IL-4	*	32	*	36
IL-8	43.8 [21.5–105.2]	32	39.8 [21.5–159.3]	36
VEGF	15.6 [15.6–28.6]	32	26.1 [15.6–62.4]	36

Values are given as median and interquartile ranges or as mean ± standard deviation.

^¤^ n = 62 (82,7%) measurements ≤ 24.8 (below the assay limit of detection)

^#^ n = 71 (94,7%) measurements ≤ 13.2 (below the assay limit of detection)

^§^ n = 74 (98,7%) measurements ≤ 18 (below the assay limit of detection)

* n = 75 (100%) measurements ≤ 20.4 (below the assay limit of detection)

Definition of abbreviations: CCL, chemokine ligand; IFN, Interferon; IL, Interleukin; MCP, monocyte chemoattractant protein); MIP, macrophage inflammatory protein; ra, receptor antagonist; TNF, tumor necrosis factor; VEGF, vascular endothelial growth factor.

**Fig 2:**
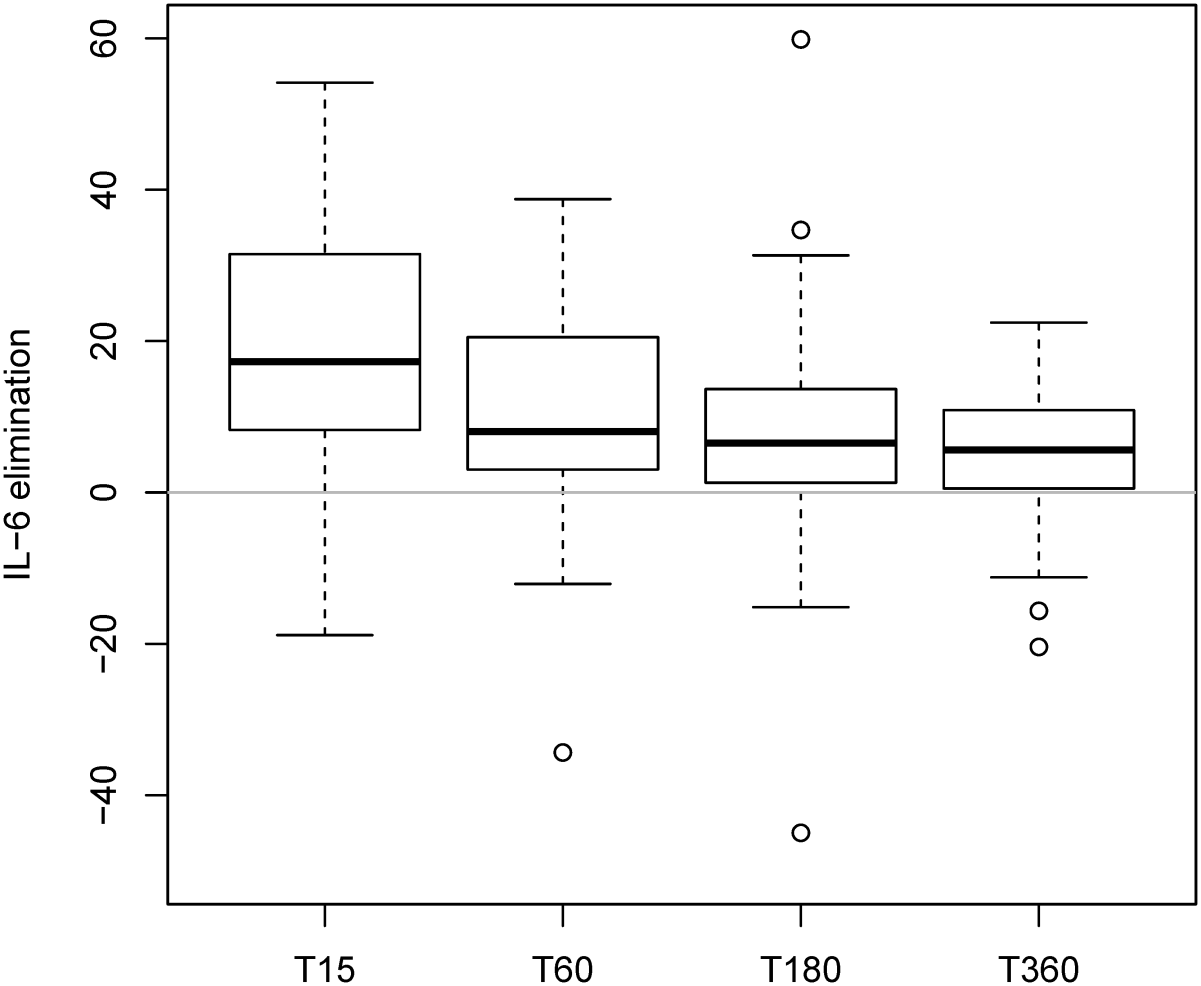
Interleukin-6 elimination (calculated as difference of arterial and venous Interleukin 6 values) in the treatment group studied during a specific kinetic day where Interleukin-6 measurements were performed before the start of the study intervention (T0), 15 minutes (T15), 60 minutes (T60), 180 minutes (T180) and 360 minutes (T360) after the start of the hemoperfusion.

**Fig 3:**
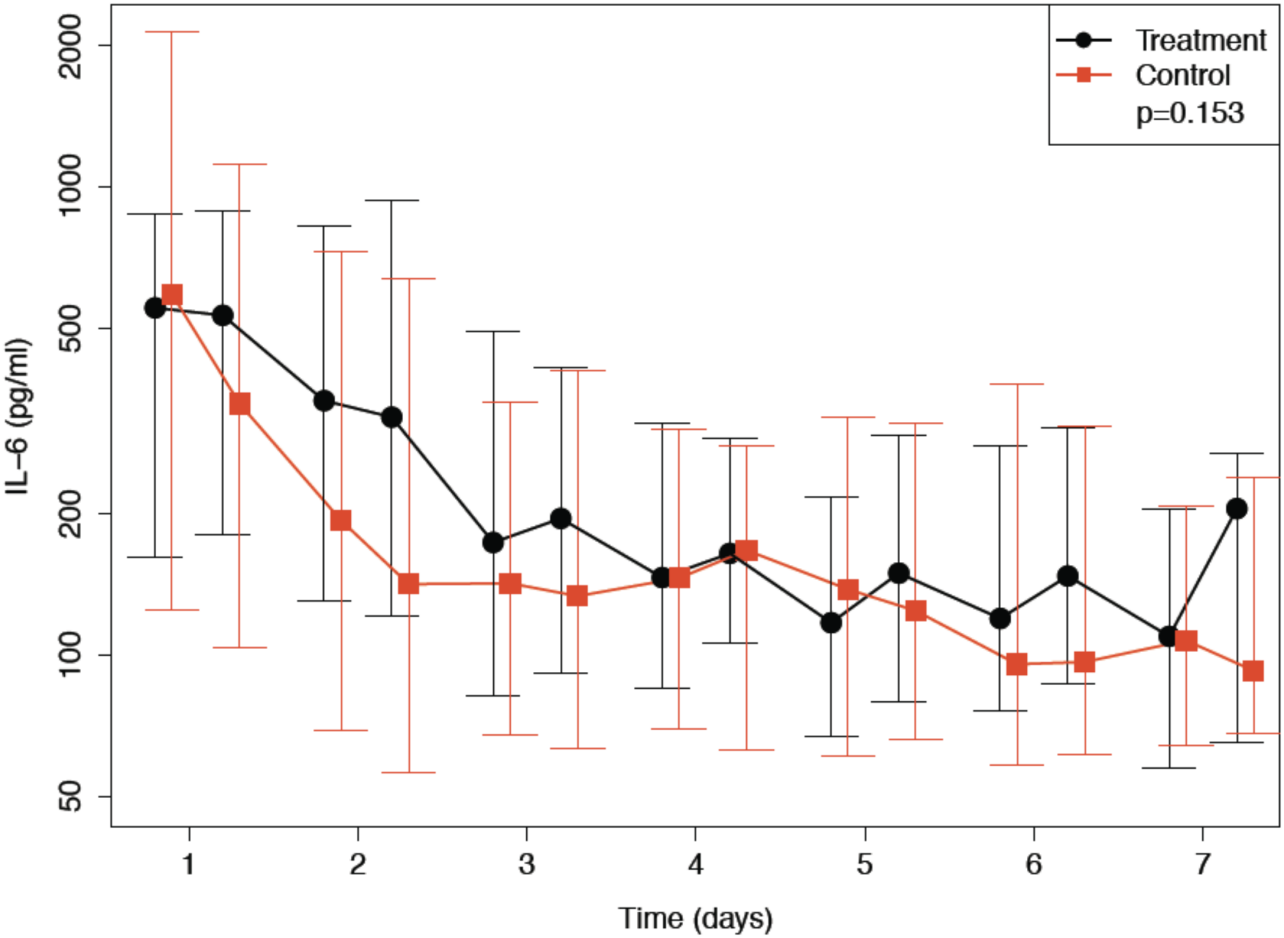
Median and interquartile range for Interleukin-6 (IL-6) plasma levels in the treatment and in the control group (n = 75).

Median IL-6 elimination rates by the CytoSorb device ranging from 5 to 18% per pass of blood through the device throughout the entire Day 2 treatment period were observed and are shown in Fig 2. There was no statistically significant effect of hemoperfusion on the log-transformed IL-6 levels (p = 0.153) (Fig 3).

**Fig 4:**
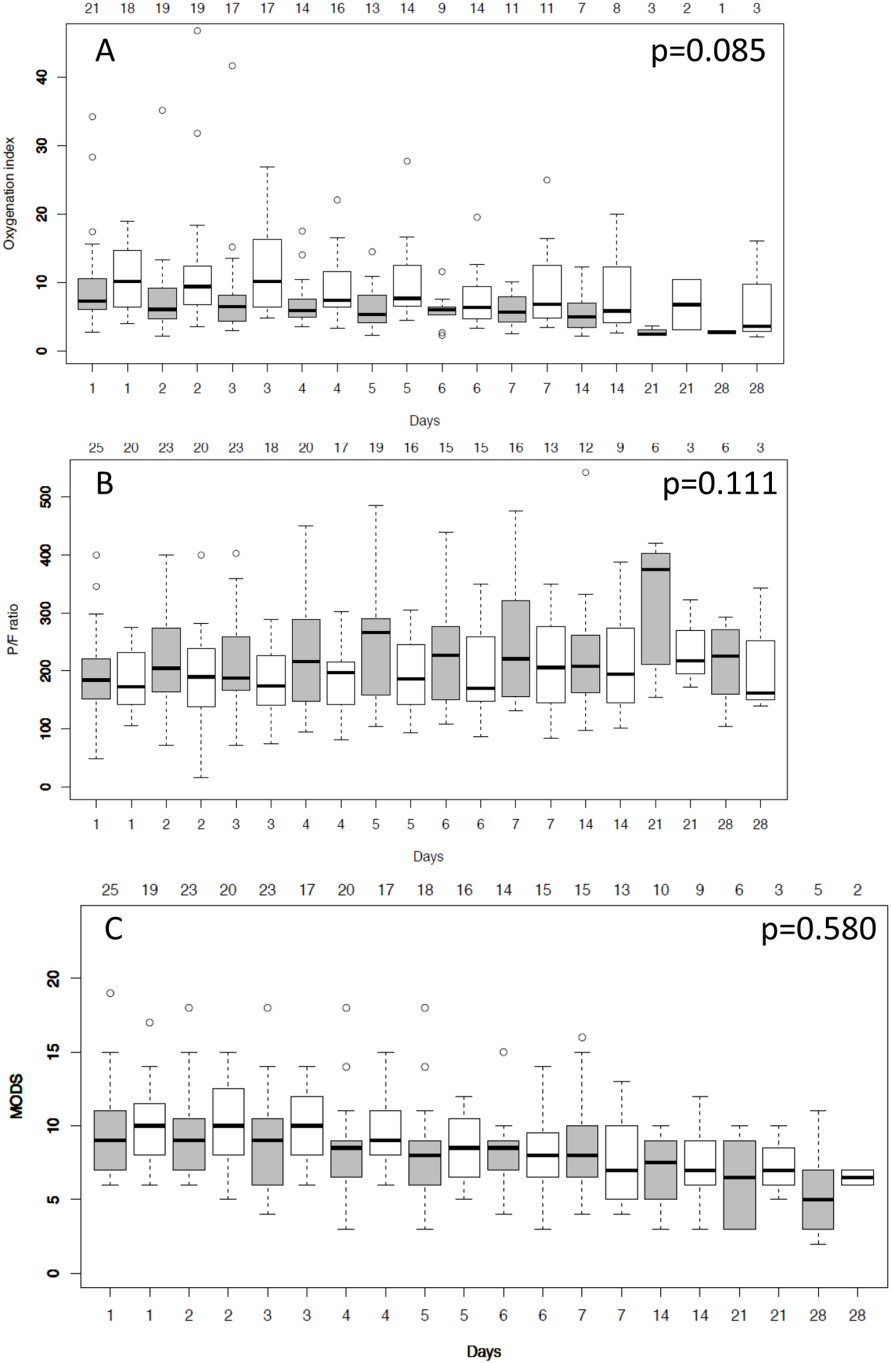
Time course for log-transformed oxygenation index (Panel A), the ratio of arterial partial pressure of oxygen and inspired fraction of oxygen (P/F ratio) (Panel B) and multiple organ dysfunction score (MODS) (Panel C) in the control group (grey boxes) and in the treatment group (white boxes). The number of patients available are displayed in the header of each panel.

**Fig 5:**
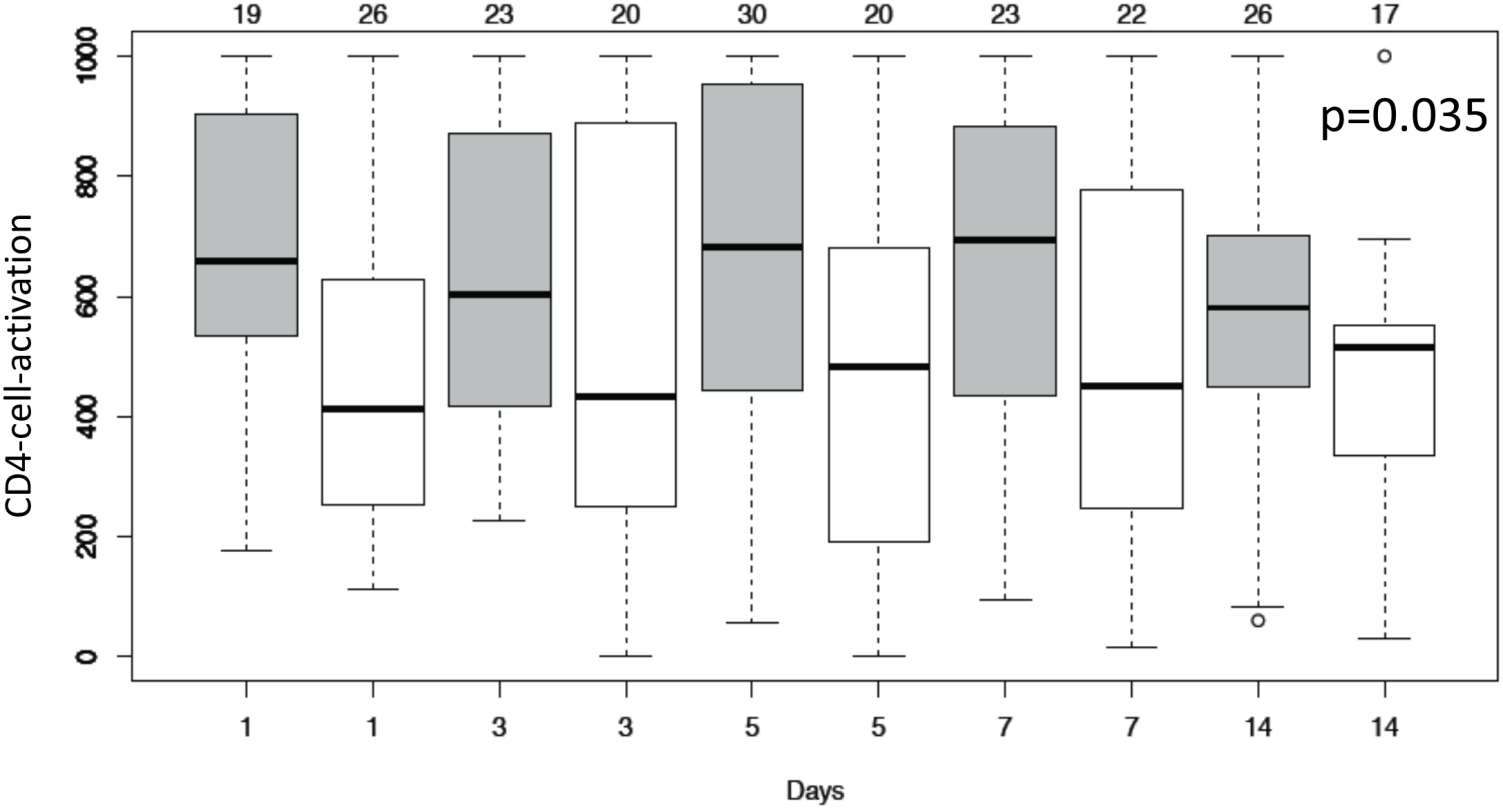
CD4-cell-activation for control (grey boxes) and treatment group (white boxes) on different study days. Values > 1000 are evaluated as 1000, values <1 as 1. The number of patients available are displayed in the header of each panel.

**Fig 6:**
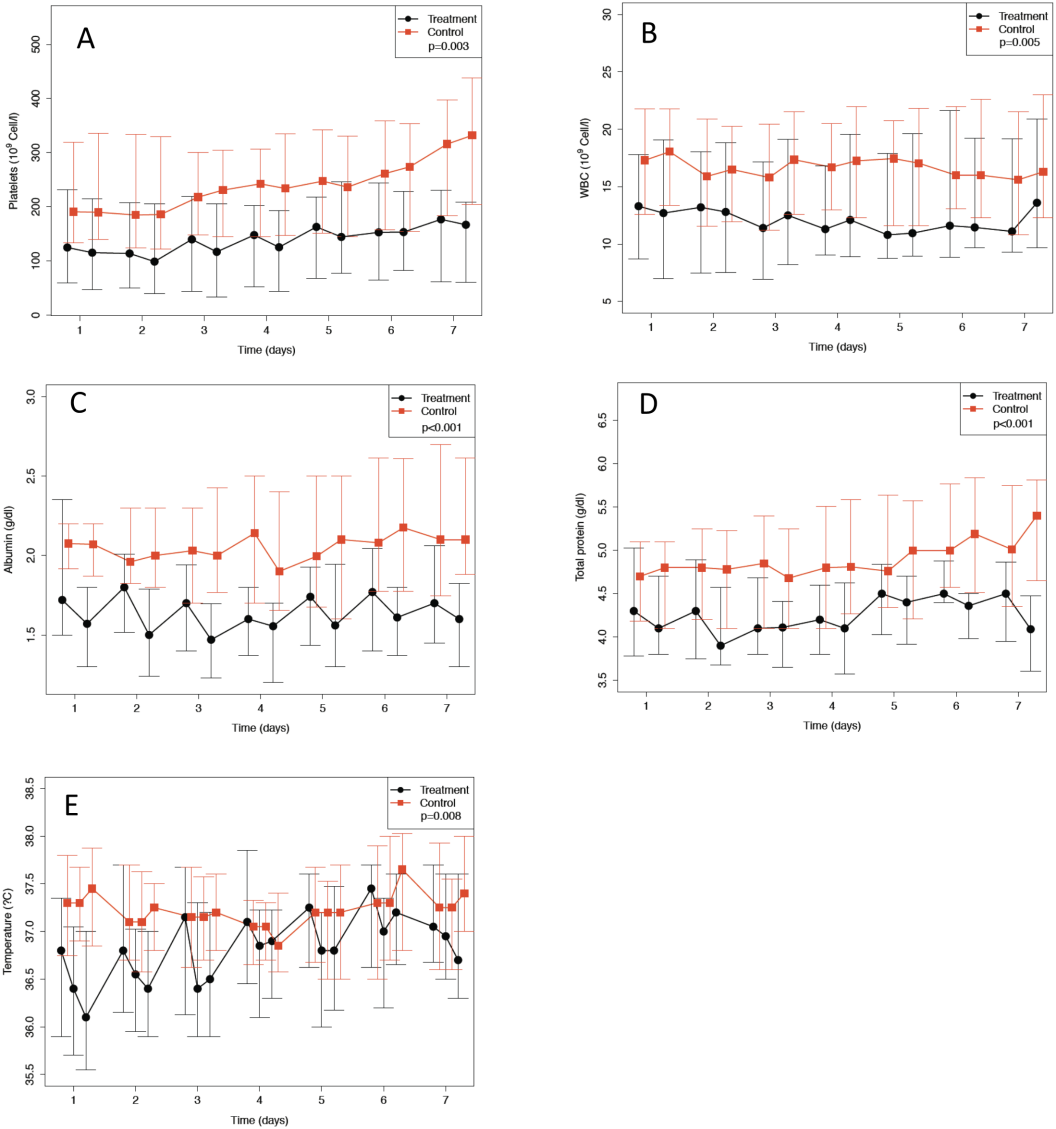
Median and interquartile ranges of platelets (Panel A), white blood cells (WBC) (Panel B), albumin (Panel C), total protein (Panel D) and body temperature (Panel E) of treatment and control group for study day 1 to 7 before and after the treatment if applicable.

Secondary endpoints including log-transformed Il-10, CCL2 (MCP-1), CCL3 (MIP-1α), Il-1ra, Il-8 and VEGF levels were not statistically different when compared with the control group (Table 5). Median ventilation time did not differ between the treatment (17.5, interquartile range (IQR): 8.6–25.9 days) and the control group (12.1 IQR: 7.0–23.1 days; p = 0.306). The time course of oxygenation index, P/F-ratio and MODS did not differ between groups (Fig 4). CD4-cell-activation was significantly lower in the treatment group when compared to controls (p = 0.035, Fig 5). Due to the small number of patients with detectable plasma levels for TNF-α, Il-1β, IFN-γ and Il-4, these endpoints were not evaluated. No interruption during the treatment period was recorded due to technical failure of the hemoperfusion device. Multivariate analysis identified study day, study day^2^ and study day^3^ but not the study intervention, study time-point T360, APACHE II score and renal replacement therapy as independent risk factors for interleukin-6 values (Table 6). We further analyzed the time course of vital parameters and several laboratory values (S4 Table). Statistically significant differences between groups were found for platelet count, white blood cell count, albumin, total protein and body temperature. The time courses for these variables are shown in Fig 6.

**Table 5:** Assessment of the treatment effect (treatment group) on cytokines in patients with available data for the primary endpoint (n = 75).

	**IL-6**		**IL-10**	
	Coefficient	P value	Coefficient	P value
Treatment group	0.3690	0.153	-0.0019	0.903
Timepoint T360	0.0275	0.533	-0.0004	0.985
Day	-0.9939	**<0.001**	0.0116	0.892
Day^2^	0.1958	**<0.001**	-0.0164	0.467
Day^3^	-0.0129	**0.001**	0.0017	0.346
	**CCL2**		**CCL3**	
	Coefficient	P value	Coefficient	P value
Treatment group	-0.1896	0.243	0.1103	0.302
Timepoint T360	-0.0551	0.079	0.0157	0.288
Day	0.0063	0.963	-0.0089	0.888
Day^2^	-0.0434	0.222	-0.0069	0.680
Day^3^	0.0043	0.139	0.0006	0.636
	**IL-1ra**		**IL-8**	
	Coefficient	P value	Coefficient	P value
Treatment group	-0.0222	0.885	-0.0094	0.947
Timepoint T360	0.1116	**0.002**	0.1091	**<0.001**
Day	-0.0797	0.603	0.0745	0.562
Day^2^	-0.0070	0.864	-0.0268	0.436
Day^3^	0.0010	0.767	0.0021	0.442
	**VEGF**			
	Coefficient	P value		
Treatment group	0.0813	0.565		
Timepoint T360	-0.1039	**0.004**		
Day	-0.2829	0.060		
Day^2^	0.0619	0.126		
Day^3^	-0.0042	0.203		

All values were log-transferred. Definition of abbreviations: CCL, chemokine ligand; IL, Interleukin; ra, receptor antagonist; VEGF, vascular endothelial growth factor.

**Table 6:** Multivariate analysis with endpoint normalized Interleukin 6 values of patients with available data for the primary endpoint.

	Coefficient	P Value
Treatment group	0.2739	0.281
Timepoint 360	0.0207	0.656
Day	-0.8167	**<0.001**
Day^2^	0.1530	**0.004**
Day^3^	-0.0098	**0.021**
APACHE II Score	-0.0417	0.181
Renal replacement therapy	-0.2108	0.480
Age	0.0076	0.496
Male sex	-0.0827	0.772

Definition of abbreviation: APACHE, Acute Physiologic and Chronic Health Evaluation

## Discussion

This is the first multicenter randomized controlled study investigating the effect of CytoSorb, a novel sorbent hemoadsorption device in a cohort of septic patients with ALI or ARDS. The CytoSorb device appeared to be safe. IL-6 elimination rates ranging from 5–18% throughout the entire 6-hour period were observed. However, hemoadsorption plus standard of care did not decrease IL-6 levels plasma levels compared to standard of care alone in this critically-ill patient population with primarily septic shock, ARDS, and multi-organ failure. Lower platelet counts, lower total protein levels, lower albumin levels and lower body temperature were observed in the treated group. However, there was no difference in the change from baseline in these values indicating the device had no impact on these values. After adjusting for comorbidities, renal replacement therapy, age and gender, treatment with the hemoperfusion device showed no association with mortality.

In the present study, systemic IL-6 levels did not differ between groups. The current weight of literature regarding the effect of different devices on systemic IL-6-levels is indifferent. Some studies found a decrease in systemic IL-6 levels [4, 17, 19, 24–26], others found no difference. In the light of the cytokinetic theory described by Honore et al. [27], this is not an unusual finding. According to this theory, blood levels for cytokines may remain stable while being extracted from the blood caused by a cytokine shift from the interstitium into the blood compartment. This is exacerbated by continuous production of cytokines during the 6-hour treatment period as well as during the 18 hours off treatment each day. which is in line with our finding of a substantial Il-6-removal of the investigated device. One may speculate that longer periods hemoperfusion during several days may be more effective in decreasing systemic IL-6 plasma levels. As cytokine release during sepsis is continuous, a continuously application of hemoperfusion may also be a further therapeutic option which has to be investigated further.

A further possible explanation for our result may be the fact, that this study was not specifically focused on patients being in a phase with high cytokine levels. In the MONARCS trial, the application of anti-TNF-α did only improve outcome in the group of patients with IL-6 levels greater than 1000 pg/ml [28]. In our study, the median IL-6-level was only 565 pg/ml (all patients) at the time point of study inclusion. It has been shown that in patients with higher IL-6 levels (IL-6 > 1000 pg/ml) hemoperfusion as well as high-adsorption CVVH leads to a significant decrease in systemic IL-6-Levels [29–32]. Given that blood purification uses concentration dependent clearance, a further study with the CytoSorb device may focus on patients with higher IL-6 plasma levels.

The CytoSorb device has been investigated *in vitro* and *in vivo* [15, 17]. These studies showed that the device is highly effective in removing cytokines from the blood. In our study, the device also successfully eliminated IL-6 but elimination slowed down during the 6-hour-treatment period (Fig 2), potentially due to limited capacity of the device, or more likely, related to decreased circulating concentrations over time. Historically, 6 hours of hemoperfusion is long compared to other studies where hemoperfusion was performed for one period of 2 hours [33], for two periods of 2 hours [13, 14], or for 4–6 hours [34]. In recently published studies, CytoSorb was applied during a 4 hours lasting treatment period. We therefore decided to apply CytoSorb during 6 hours per day suggesting that extraction rates will remain constant throughout the study period. The efficacy of longer treatment in septic patients with high proinflammatory cytokine concentrations should be evaluated.”

We used normalized IL-6 serum concentration as primary endpoint because we expected to receive detectable IL-6 serum concentrations in all patients during the whole study period. Serum concentrations for TNF-α and IL-1 were not detectable in 38 to 40% of blood samples in animal experiments [17]. IL-6 is consistently detected in septic patients and increased plasma concentrations are associated with higher mortality [35].

IL-6 elimination rates were determined on study day 2. One may hypothesize that IL-6 elimination rates would have been higher on study day 1 as serum IL-6 levels were much higher (Fig 3). The basic idea of selecting study day 2 for assessing IL-6 elimination rate was, that an elimination rate should generally be independent of pre-device IL-6 serum levels. Sensitivity of the IL-6 assay is probably the only factor influencing the data in cases where at the time of measurement pre-device IL-6 levels are already low and post-device levels are reduced to levels below detection level of the assay. In this case the elimination rate may be calculated falsely low. Study day 2 was selected for assessment of IL-6 elimination rate as a mid-term treatment day. Any other day could have been chosen in general. We do not think that selecting another day for assessment of IL-6 elimination rate would have affected the results in a relevant manner.

Our study was neither designed nor powered to detect differences in mortality between the two studied groups. Furthermore, there was an imbalance between the two studied groups with a clinically significant larger number of patients needing renal replacement therapy in the treatment group (31.9% treatment group vs. 16.3% control group). Crude 60-day mortality rates differed significantly between groups. However, after adjusting for co-morbidities such as acute kidney injury requiring renal replacement therapy prior to study enrollment, hemoperfusion was not associated with mortality. We therefore cannot rule out a potential harmful effect of the studied device and recommend the application of this device to be limited to urgently needed further randomized controlled studies.

We cannot exclude that failure to detect prognostic factors for mortality is due to the relatively small sample size. However, mortality was not the primary endpoint of the study. Regarding sample size calculation, a formal a-priori sample size and power calculation was not performed because no sufficient prior information (effect sizes, variance estimates) was available when the study was planned. The present study rather aimed to generate such information to allow power calculations for future larger trials.

Clotting of polymyxin B hemoperfusion devices has been reported to occur in 6 to 11% of the performed treatments [13, 14]. In our study, no clotting of the CytoSorb device was reported. However, albumin levels and platelet counts were clinically relevant lower in the hemoperfusion group. As the differences were already present during study inclusion, we suppose that they were not caused by the studied device. While decreased levels of platelets were also found in the treatment groups of other studies involving polymyxin B hemoperfusion [14, 34], a decrease of albumin levels was not noted or not reported. The CytoSorb hemoperfusion device adsorbs molecules between 10–55 kDa. Its ability to remove albumin (65–70 kDa) and other needed or beneficial proteins from the body should be investigated in future studies.

### Study limitations

Our study has several important limitations that have to be mentioned. First, we were not able to correctly measure IL-6-plasma levels in 22 patients. Second, blinding was not feasible due to the nature of the study. Third, extended hemodynamic monitoring variables like cardiac output, cardiac index and systemic vascular resistance were not noted. Fourth, we recruited patients from April 2008 until April 2011. Due to changes in the management of sepsis since 2008, the results may have been different if the study had been performed more recently.

## Conclusions

In this multicenter randomized study treatment with CytoSorb, a novel extracorporeal cytokine adsorber, lead to a substantial removal of Il-6 in a severely ill patient population with primarily septic shock, ARDS, and multi-organ failure. However, this did not result in decreased normalized IL-6-plasma levels compared to control. Future studies should investigate safety of the device and be powered to evaluate clinical endpoints such as mortality.

## Supporting information

S1 File Study protocol. (PDF)

S2 File CONSORT checklist. (DOCX)

S3 File Inclusion and exclusion criteria. (DOCX)

S4 File Study sites. (DOCX)

S1 Table Demographic data and baseline characteristics of the study patients with available data for the primary endpoint. Values are given as median and interquartile ranges or as mean ± standard deviation unless otherwise noted. * Missing subscores on the Acute Physiologic and Chronic Health Evaluation (APACHE II Score) were counted as 0. Definition of abbreviations: MODS, multiple organ dysfunction score; P/F, arterial partial pressure of oxygen divided by inspired fraction of oxygen.
(DOCX)

S2 Table Baseline hemodynamic variables, blood gas values and laboratory values of patients with available data for the primary endpoint. Values are given as median and interquartile ranges. Definition of abbreviations: GOT, glutamic oxaloacetic transferase; GPT, glutamate pyruvate transferase; Gamma-GT, gamma glutamyl transpeptidase; LDH, lactate dehydrogenase.
(DOCX)

S3 Table Baseline mechanical ventilation therapy and blood gas values of patients with available data for the primary endpoint. Values are given as median and interquartile ranges or as mean ± standard deviation unless otherwise noted. Definition of abbreviations: APRV, airway pressure release ventilation, HCO3, sodium bicarbonate; HFOV, high frequency oscillation ventilation; PaCO2, arterial partial pressure of carbon dioxide; PaO2, arterial partial pressure of oxygen; pbw, predicted body weight; PCV, pressure controlled ventilation; P-F-ration, arterial partial pressure of oxygen divided by inspired fraction of oxygen; PSV, pressure support ventilation.
(DOCX)

S4 Table Analysis of the time course of vital parameters and laboratory values of patients with available data for the primary endpoint. The effect of treatment for each variable was analyzed by a mixed model which was adjusted for day or day and timepoint if available. ^#^ values were log-transformed. Definition of abbreviations: GOT, glutamic oxaloacetic transferase; GPT, glutamate pyruvate transferase; Gamma-GT, gamma glutamyl transpeptidase; HCO3, sodium bicarbonate; LDH, lactate dehydrogenase; paCO2, arterial partial pressure of carbon dioxide; PaO2, arterial partial pressure of oxygen.
(DOCX)
