# Supplementary material for: The effect of a novel extracorporeal cytokine hemoadsorption device on IL-6 elimination in septic patients: A randomized controlled trial
Source: PLoS One. 2017 Oct 30;12(10):e0187015. doi: 10.1371/journal.pone.0187015 (PMC5662220; doi:10.1371/journal.pone.0187015)
Supplement: S1 File — (PDF) [file pone.0187015.s001.pdf]

PROPOSED MULTI-CENTER TRIAL

Title: **Multi-Center, Efficacy Study of the MedaSorb CytoSorb™ Hemoperfusion Device as an Adjunctive Therapy in Subjects with Acute Respiratory Distress Syndrome (ARDS) or Acute Lung Injury (ALI) in the Setting of Sepsis.**

Protocol Number 2007-01
Version 4.1: June 04, 2009

Product: **MedaSorb CytoSorb 300 ml Device**

Study Sponsor<br><br><br><br><br><br>**Sponsor Contact:**	MedaSorb Technologies, Inc.<br>7 Deer Park Drive, Suite K,<br>Monmouth Junction, NJ 08852<br>Tel : 732-329-8885<br>Fax: 732-329-8650<br><br>Robert Bartlett, M.D.<br>Tel: 734-998-0173
Principal Investigator	Prof. Dr. med. Martin K. Kuhlmann<br>Klinikdirektor Innere Medizin – Nephrologie<br>Vivantes Klinikum im Friedrichshain<br>Landsberger Allee 49<br>D-10249 Berlin<br><br>Tel: 030-4221-1004<br>Fax: 030-4221-2046
Study Monitor/Data Management	MedPass International<br>95 bis Boulevard. Pereire<br>75017 Paris, France<br>Tel : 33 01 42 12 83 30<br>Fax : 33 01 40 53 81 11

APPROVAL SIGNATURE SPONSOR

Robert Bartlett, M.D.
Chief Medical Officer
MedaSorb Technologies, Inc.

Signature ____ Date ________________

Confidentiality Statement
The confidential information in this document is provided to you as an investigator or consultant for review by you, your staff, and the applicable Institutional Review Board/Independent Ethics Committee. Your acceptance of this document constitutes agreement that you will not disclose the information contained herein to others without written authorization from MedaSorb Technologies, Inc

SIGNATURE(S) OF INVESTIGATOR(S)

Prof. Dr. med. Martin K. Kuhlmann – Principal Investigator
Klinikdirektor Innere Medizin – Nephrologie
Vivantes Klinikum im Friedrichshain
Landsberger Allee 49
D-10249 Berlin

I have read the protocol and agree that it contains all necessary details for me to carry out the study. I agree to conduct the study in accordance with the protocol, and to fulfill my responsibilities as outlined in the Declaration of Helsinki.

____

Investigator's Name (Print Name)

____

Investigator's Signature

____

Date

Table of Contents

PROPOSED MULTI-CENTER TRIAL **1**
APPROVAL SIGNATURE SPONSOR **1**
SIGNATURE(S) OF INVESTIGATOR(S) **2**
PROTOCOL SYNOPSIS **6**
LIST OF ABBREVIATIONS **11**
LIST OF ABBREVIATIONS **11**
TERMINOLOGY **12**
PROTOCOL **16**
1 STUDY OBJECTIVES **16**
1.1 OBJECTIVES **16**
1.2 BACKGROUND CYTOSORB DEVICE **16**
1.3 PRIOR CLINICAL EXPERIENCE IN SEPTIC APPLICATION **17**
2 INVESTIGATIONAL PLAN **21**
2.1 STUDY DESIGN: **21**
2.2 RISK ANALYSIS **21**
2.2.1 Risks of Study Procedures *21*
2.2.2 Potential Benefits to Subject *21*
2.2.3 Minimization of Risks *22*
2.2.4 Justification for the Study *22*
2.2.5 Description of the Subject Population *22*
2.3 SELECTION OF STUDY POPULATION **23**
2.3.1 Inclusion Criteria *23*
2.3.2 Exclusion Criteria *23*
2.4 ENDPOINT ANALYSIS **24**
2.4.1 Analysis *24*
2.4.2 Sample Size *25*
2.4.3 Study Population *25*
3 ADMINISTRATIVE AND TREATMENT PROCEDURES **26**
3.1 ADMINISTRATIVE PROCEDURES **26**
3.1.1 Informed Consent Document Form *26*
3.1.2 Source Document Binder Contents *26*
3.1.3 Equipment Calibration *26*
3.2 TREATMENT PROCEDURES **26**
3.2.1 Screening Evaluation (Day -1, -2, -3) *26*
3.2.2 Hemoperfusion Treatment (Day 1-7) *27*
3.2.2.1 Blood Sampling 28
3.2.3 Combined Treatment and Dialysis Alone (Control Subject) *28*
3.2.4 Post Treatment Drug Dosing *30*
3.2.5 Ventilation and Weaning *30*
3.2.5.1 Ventilation Modes 31
3.2.5.2 Definition and Method of PaO_2/FiO_2 (P/F) Ratio Measurement 31
3.2.6 Glasgow Coma Scale Scoring *31*
3.3 EFFICACY EVALUATION **31**
3.3.1 Intra-Session Reduction Studies *31*

3.3.2 Cytokine/Drug/Hormone Kinetic Analysis *31*
3.3.3 Heparinization and Regional Citrate Anticoagulation *32*
3.3.3 .1 Heparinization 32
3.3.3.2 Regional Citrate Anticoagulation 32
3.3.4 Subjects Using Catheters for Vascular Access *32*
3.3.5 Archive Samples *34*
3.3.6 Complement Activation Studies (Sites as specified) *34*
3.3.7 Arterial Blood Gases *34*
3.3.8 Thrombus Formation *34*
3.3.9 HIT Antibody/D Dimer (Sites as specified) *34*
3.3.10 Immune System CD4 Cell Activation *35*
4 SUBJECT TRIAL INTERRUPTION, DISCONTINUATION OR EARLY DEPARTURE FROM HOSPITAL (< 28 DAYS) **36**
4.1 MISSED SCHEDULED TREATMENT **36**
4.2 SUBJECT DISCONTINUATION **36**
4.2.1 Severe Adverse Events (SAEs) *37*
4.3 TRIAL DISCONTINUATION **37**
4.4 EARLY DEPARTURE FROM HOSPITAL **37**
5 DATA MANAGEMENT **38**
5.1 DATA COLLECTION **38**
5.2 ELECTRONIC CASE REPORT FORMS (ECRFS) **38**
6 ANALYSIS PLAN **39**
6.1 ENDPOINTS **39**
6.2 SAMPLE SIZE **39**
6.3 STUDY POPULATION **40**
6.3.1 Standard Statistical Methods *40*
6.3.2 Demographic and Baseline Screening Characterization *40*
6.3.3 Analysis of Study Endpoints *40*
6.3.3.1 Prospective Comparison 40
6.3.4 Safety Analysis *41*
6.4 STUDY DURATION **42**
6.5 DATA SAFETY MONITORING BOARD **42**
7 ETHICS/ ETHICAL REQUIREMENTS **43**
7.1 ETHICS COMMITTEE **43**
7.2 ETHICAL CONDUCT OF THE STUDY **43**
8 CLINICAL MONITORING **45**
8.1 CLINICAL MONITORING PROCEDURES **45**
8.1.1 DATA QUALITY ASSURANCE **46**
8.1.2 Anticipated Adverse Events *46*
9 INVESTIGATOR RESPONSIBILITIES **47**
9.1 INVESTIGATOR RECORDS **47**
9.2 INVESTIGATOR REPORTS **47**
9.2.1 Severe Adverse Events (SAEs) *47*
9.2.2 Withdrawal of Ethics Committee Approval *47*
9.2.3 Deviation from the Investigational Plan *48*
9.2.4 Use of the CytoSorb Device Without Informed Consent *48*
9.2.6 Final Report *48*
9.2.7 Other Reports *48*

9.2.8 Publishing of Data *48*
9.2.9 Investigator Materials Accountability *48*
10 SPONSOR RESPONSIBILITIES 49
10.1 Beginning the Study *49*
10.2 Selecting Investigators *49*
10.3 Control of Investigational Devices *49*
10.4 Obtaining Agreements *49*
10.5 Securing Compliance *49*
10.6 Severe Adverse Events (SAEs) *49*
10.7 Withdrawal of EC/CA approval *50*
10.8 Current Investigator List *50*
10.9 Progress Reports *50*
10.10 Other Reports *50*
11 REFERENCES 51
12 APPPENDIX 1: SAMPLE INFORMED CONSENT 52
13 APPENDIX II - HELSINKI DECLARATION (LATEST VERSION) 63
14 APPENDIX III – LIST OF POTENTIAL INVESTIGATORS 68
15 APPENDIX IV – ARDSNET VENTILATION AND WEANING GUIDELINES 71
16 APPENDIX V – DATA SAFETY MONITORING BOARD MEMBERSHIP .. 72

PROTOCOL SYNOPSIS

**Title**:	**Multi-Center, Efficacy Study of the MedaSorb CytoSorb™ Hemoperfusion Device as an Adjunctive Therapy in Subjects with Acute Respiratory Distress Syndrome (ARDS) or Acute Lung Injury (ALI) in the Setting of Sepsis.**
**Protocol Number:**	2007-01
**Principal Investigators:**	Site 01 - Martin Kuhlmann, M.D.
**Phase:**	Efficacy Trial
**Indication**:	For use as adjunctive treatment of subjects with acute respiratory distress syndrome (ARDS) or Acute Lung injury (ALI) to improve lung function.
**Study Design:**	Subjects will be randomized to treatment or control arms of the study. Up to a total of 100 subjects will be enrolled in order to collect complete data sets on 30 subjects that receive CytoSorb™ (CytoSorb) treatment and 30 control subjects that do not receive CytoSorb treatment. The treatment group will receive a 6 hour period of hemoperfusion on a daily basis with CytoSorb up to 7 days or successful weaning (48 hours unassisted breathing) from the ventilator which ever comes first. If hemodialysis is required during the study period, CytoSorb will be placed in-line proximal to the dialysis device.

The CytoSorb clinical trial will comply with current International Conference on Harmonization (ICH) of Good Clinical Practices (GCP) Guidelines

A Schematic of the Study Design for Treated Subjects: (Up to 40 subjects)

A Schematic of the Study Design: Treatment Group

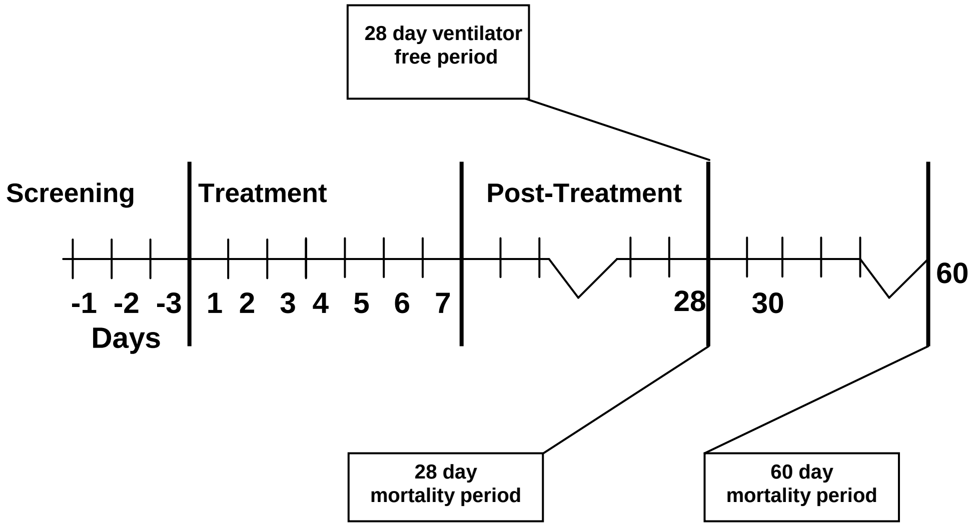

A Schematic of the Study Design for Concurrent Control Subjects: (Up to 40 subjects)

A Schematic of the Study Design: Control Group

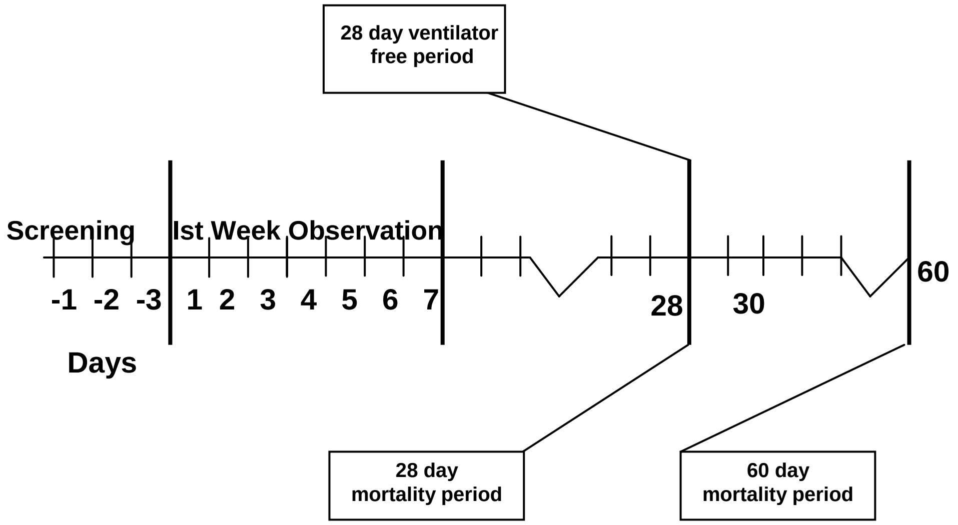

Population: Subjects with diagnosis of ALI or ARDS will be eligible for this study. Additionally, subjects must meet the following inclusion/exclusion criteria:

Inclusion criteria:

- Signed informed consent document (ICD)
- Male or female ≥ 18 and ≤ 80 years of age.
- Subjects must have diagnosis of ARDS or ALI, based on ARDSNet Definition, established within last 72 hours, confirmed by clinical, radiological, or physiologic findings [1]
- Subject must be intubated
- ≤ 3 days on a ventilator prior to enrollment
- Subjects must have confirmed diagnosis of sepsis [2]
- Subject must have had at least 24 hours of antibiotic therapy
- Pre-menopausal female subjects must have negative pregnancy test.
- Subject must be available for periodic blood sampling, study related assessments, and management at the treating institution for the duration of the study. Subject must have permanent home address to allow completion of 60 day follow-up.
- Subject or health care proxy has the ability to understand and willingness to sign the informed consent form.

Exclusion criteria:

- Currently participating in another clinical study involving investigational chemical compound, biologic, or device within the last 30 days prior to the start of this trial.
- ARDS Net Exclusion Criteria
  - Neuromuscular disease that impairs the ability to ventilate spontaneously, such as C_5 or higher spinal cord injury, amyotrophic lateral sclerosis, Guillain-Barré syndrome and myasthenia gravis.
  - Increased intracranial pressure, tricyclic antidepressant overdose, hemoglobin SS, hemoglobin SC or other conditions where hypercapnia would be contraindicated.
  - Severe chronic respiratory disease including hospitalization within last 6 months for respiratory failure.
  - Morbid obesity (Body Mass Index ≥40 kg/m^2).
  - Burns > 30% BSA, bone marrow transplant, lung transplant or end stage hepatic liver failure.
- Subject with mean arterial pressure ≤ 60 mmHg and is not responsive to vassopressors.
- Subject with active malignancy receiving chemotherapy or radiation treatment within last 60 days.

---

[1] ARDS Net Definition: **Bilateral abnormalities** on chest X-Ray, No evidence of congestive heart failure, pulmonary capillary wedge pressure ≤ 18 mm Hg (if available), P/F ratio ≤ 300 mm Hg, Mechanical ventilation required. **Bilateral abnormality: for purposes of the sepsis trial the chest Xray reading of the treating physician or PI at the time of enrollment is considered the definitive reading. "Bilateral abnormality" of the lungs is the criterion for inclusion. For example, a patient who has lobar pneumonia or unilateral chest trauma AND abnormality in the contralateral lung can meet the ALI_ARDS definition**

[2] **Sepsis:** SIRS plus known or suspected infection source, blood cultures need not be positive, bacteremia need not be present. **Systemic Inflammatory Response Syndrome (SIRS):** Subject presents with two or more of the following criteria - heart rate > 90 beats per minute, body temperature < 36 or > 38°C, respiratory rate > 20 breaths per minute or, P_aCO_2 < 32 mm Hg, white blood count < 4 x 10^9 or > 12 x 10^9 cells/L), or the presence of greater than 10% immature neutrophils.
The diagnosis of infection may be made with cultures and Gram stains of biological fluids, pathological and surgical specimens, or radiology procedures.

- Subjects with AIDS, CD4 count of < 200 or 14%, or the presence of an AIDS defining illness (HIV+ subjects may be enrolled)
- Subject with acute coronary syndrome.
- Subjects with decompensated heart failure with New York Heart Association (NYHA) classification IV
- Subjects with Chronic Kidney Disease (CKD) stage 5 will be excluded
- Subjects with end stage hepatic liver failure
- Subjects on immunosuppressive agents, excluding corticosteroids
- Platelets ≤ 20,000/mm^3
- Subjects on anti-TNF therapy
- Subjects about to receive or receiving drotrecogin alpha (Xigris) therapy
- Subject is pregnant or breastfeeding.
- Subject has a known allergy to any component of the CytoSorb hemoperfusion device
- Subject has any active disease condition that could limit compliance with the study procedure, including but not limited to the following: acute coronary syndrome, life-threatening cardiac arrhythmia, or psychiatric or social conditions, considered by investigator(s) to preclude successful completion of the study.

Sample Size[1]:

Up to a total of 100 subjects will be enrolled in order to collect complete data sets on 30 subjects that receive CytoSorb treatment and 30 control subjects that do not receive CytoSorb treatment.

Duration of Study:

The study will involve a maximum of 60 days per subject, including up to 7 consecutive days of CytoSorb treatment followed by scheduled blood draws with observation for 28 days and follow-up on patient condition at 60 days. MedaSorb believes it will take approximately one year to complete this study in up to 15 investigational sites.

Effectiveness Criteria for Evaluation:

- Subjects treated with CytoSorb will have lower relative IL-6 levels than control subjects receiving only the standard of care.
- Ventilation support and safety measures will be recorded and evaluated throughout the study.
- Multiple organ dysfunction score (MODS score), cytokines, Oxygen Index, PaO_2/FiO_2 ratios, adverse events (AE) and 28 day mortality will be measured. As exploratory information, Acute Physiology and Chronic Health Evaluation score (APACHE II), SOFA score, pressor use and 60 day mortality will be collected.
- Refer to List of Abbreviations
- Refer to the Terminology section for definition of Treatment Period.

Safety Criteria for Evaluation:

- The safety of CytoSorb will be evaluated on both clinical and device-based parameters. Clinical parameters will include the occurrence of adverse events, the monitoring of vital signs, and

[1] To date there have been 22 patients enrolled in the trial. Eleven (11) subjects have been enrolled in the treatment arm and eleven (11) in the control arm. Cytokine samples were derived from Serum samples using Serum clot tubes requiring 1 hour sit time prior to serum collection. We have found information in the literature and in our own laboratory testing that shows sample tube in-vitro cytokine generation is possible. The cytokine data collected from the first 22 patients is suspect due to possible in-vitro generation of cytokines resulting from the 1 hour sit time to establish a clot. MedaSorb has changed to Plasma blood collection tubes (EDTA) which allows for immediate processing to mitigate the possibility of in-vitro cytokine generation; this was done after patient 22. The first 22 patients will be used for purposes of safety data analysis and not included in the final data set for cytokine analysis.

clinical laboratory changes. Device-specific parameters will include the incidence of any device related technical issues that may adversely affect treatment, e.g., critical changes in pressure profiles of the extracorporeal circuit, that may be caused by obstructions, leaks or breaks, or any other physical failures of the device.

Vital signs, physical examinations, adverse events, and laboratory safety tests, will be obtained or assessed prior to CytoSorb treatment and at designated intervals throughout the study.

- Any serious adverse experience, including death due to any cause, which occurs in any subject entered in this study whether or not related to the investigational product, must be reported within 24 hours to one of the individuals listed on the sponsor contact information page. Additionally, any serious adverse event (SAE) considered by an investigator to be possibly, probably, or definitely related to the investigational product must be brought to the attention of the sponsor. Any SAE that occurs at any time outside of the study period considered by an investigator to be possibly, probably, or definitely related to the investigational product must also be reported immediately to one of the individuals listed on the contact information page.

LIST OF ABBREVIATIONS

ACT	Activated Clotting Time
AE	Adverse Event (experience)
ALB	Albumin
ALT	Alanine Aminotransferase
ANOVA	Analysis of Variance
APACHE II	Acute Physiology and Chronic Health Evaluation score
AST	Aspartate Aminotransferase
Beta hCG	Beta Human Chorionic Gonadotropin
CRP	C-Reactive Protein
CBC	Complete Blood Count
CA	Competent Authority
EC	Ethics Committee
ESRD	End Stage Renal Disease
cGCP	Current Good Clinical Practices
Hct	Hematocrit
HFD	High-Flux Dialyzer
HIPAA	Health Insurance Portability and Accountability Act
HIT	Heparin Induced Thrombocytopenia
IC	Informed Consent
ICD	Informed Consent Document
IFU	Instructions for Use
IDE	Investigational Device Exemption
IL	Interleukin
MODS	Multiple Organ Dysfunction Score
PHI	Protected Health Information
PI	Principal Investigator
Plt	Platelet(s)
aPTT	Partial Thromboplastin Time
Rx	Prescription
Qb	Blood Flow Rate
Qd	Dialysate Flow Rate
RBC	Red Blood Cell
SAE	Serious Adverse Event
SIRS	Systemic Inflammatory Response Syndrome
SOFA	Sequential Organ Failure Assessment
TP	Total Protein
US	United States
WBC	White Blood Cells
ARDSNet	Network of Hospitals in USA specializing in Acute Respiratory Distress Syndrome Treatment

TERMINOLOGY

1. The following terms are used to define the times for collection of blood samples:

$T_{baseline}$	before the initial loading dose of heparin into the venous line, before the start of treatment to establish the baseline aPTT
T_0	blood samples collected 5 minutes after at the start of treatment (time 0) to assure saline is cleared from lines, drawn by venous puncture for control patients or catheter/bloodline at start of treatment
T_{15}	blood samples collected at 15 minutes after the start of treatment
T_{30}	blood samples collected at 30 minutes after the start of treatment
T_{60}	blood samples collected at 60 minutes after the start of treatment
T_{180}	blood samples collected at 180 minutes after the start of treatment
T_{360}	blood samples collected at 360 minutes after the start of treatment
T_{End}	blood samples collected from the arterial line at the end of treatment, prior to returning the blood in the extracorporeal circuit to the subject

2. Throughout this protocol, the term “treatment session” will refer to any CytoSorb-only session. The term “combined treatment session” will refer to any treatment session in which CytoSorb is placed in series with a dialyzer, should dialysis become necessary in the course of the subject’s illness. Each session is referred to by the day number.

A Schematic of the Study Design for Treated Subjects: (Up to 40 subjects)

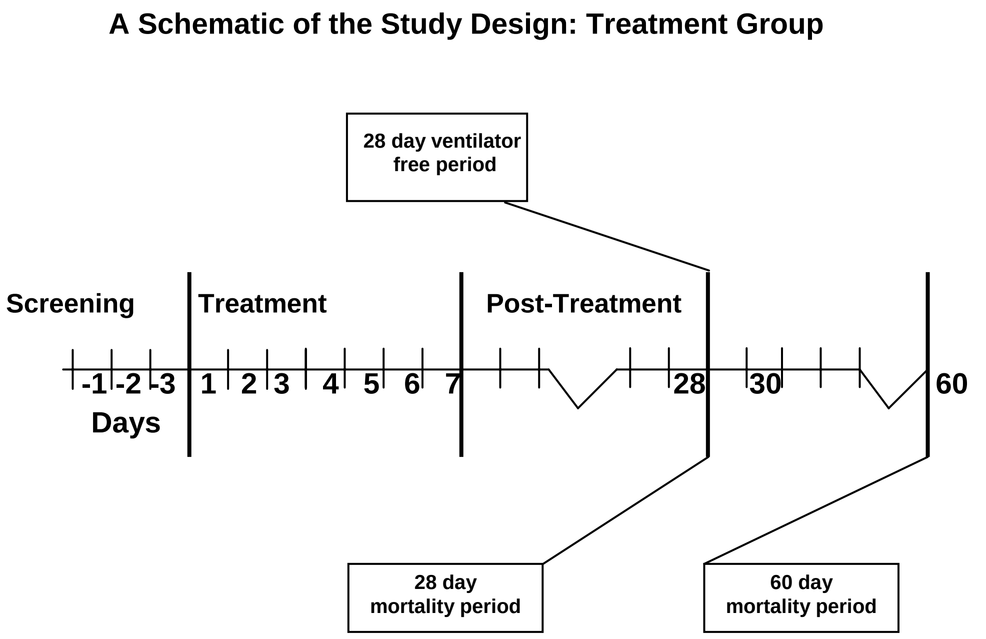

A Schematic of the Study Design for Concurrent Control Subjects: (40 subjects)

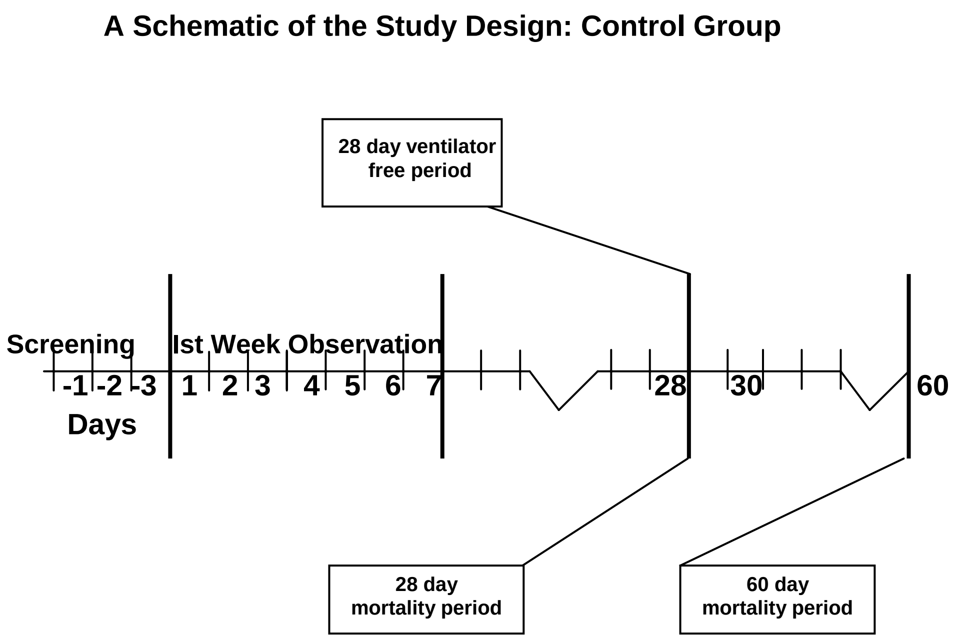

Table 1: Schedule of Events for **Treated Subjects**

Schedule	Screening[ŦŦ]	Day 1-7	Day 8-28	Day 29 - 60
Informed consent	X			
Demography & medical history	X			
Prior/concomitant medications	X	Daily	Every 7 days	
Inclusion/Exclusion criteria	X			
Weight[Ŧ]	X	ADI	ADI	ADI
Physical exam[Ŧ]	X	ADI	ADI	ADI
Vital signs[Ŧ]	X	Daily	Every 7 days	
Chest X-ray[Ŧ]	X	ADI	ADI	ADI
MODS assessment		Daily[3]	Every 7 days	
APACHE II assessment		Daily[3]	Every 7 days	
SOFA assessment		Daily[3]	Every 7 days	
Cytokine/Archive Samples		Daily	Every 7 days	
Cytokine/Drug/Hormone Kinetics[∂]		Day 2		
Blood Gases		Daily @ 0800 ± 1h	Every 7 days@ 0800 ± 1h	
Mechanical ventilation	X[1]	Daily @ 0800 ± 1h	Every 7 days@ 0800 ± 1h	
PEEP/FiO_2		Daily - Patient Record	Daily - Patient Record	
CytoSorb hemoperfusion		Daily 6 hours		
Heparinization as determined and adjusted by hourly aPTT		Daily		
Fluid Balance[2Ŧ]		Daily	Days 14, 21, 28	
Adverse Event (AE) assessment		Daily	ADI [Ŧ]	
CBC	X	Daily (2x)	Every 7 days	
Blood chemistries (Chem 20)	X	Daily (2x)	Every 7 days	
Immune System CD4 Cell Activation		Days 1, 3, 5, 7	14 and Discharge	
Urine ß-hCG in pre-menopausal females	X			
Pressor agent use[Ŧ]	Day -1	Daily	Every 7 days	
Complement (sites as specified)		Day 3		
HIT/D Dimer (sites as specified)		Day 1 and 7	NA	
Mortality		Daily	Daily	X
Ventilation days following initiation of protocol [Ŧ]			X	

ADI: As determined by intensivist

[1]Ventilation: Subject must be intubated and PaO_2/FiO_2 <300mm Hg for ARDS/ALI. Ventilation and weaning from the ventilator guidelines from ARDS Net supplied, but your hospital ventilation guidelines may differ and should be followed

[2 Ŧ]Fluid Balance = total intake – total output

Ŧ From hospital records

ŦŦ From hospital records except for β-hCG

&& From medication list for which an assay exists

∂ As described in section 3.3.2

[3]Calculated post study

Table 2: Schedule of Events for **Control Subjects**

Schedule	Screening[ꞘꞘ]	Day 1-7	Day 8-28	Day 29 - 60
Informed consent	X			
Demography & medical history	X			
Prior/concomitant medications	X	Daily	Every 7 days	
Inclusion/Exclusion criteria	X			
Weight[Ꞙ]	X	ADI	ADI	ADI
Physical exam[Ꞙ]	X	ADI	ADI	ADI
Vital signs[Ꞙ]	X	Daily	Every 7 days	
Chest X-ray[Ꞙ]	X	ADI	ADI	ADI
MODS assessment		Daily[3]	Every 7 days	
APACHE II assessment		Daily[3]	Every 7 days	
SOFA assessment		Daily[3]	Every 7 days	
Cytokine/Archive Samples		Daily	Every 7 days	
Cytokine Kinetics[∂]		NA	NA	
Blood Gases		Daily @ 0800 ± 1h	Every 7 days@ 0800 ± 1h	
Mechanical ventilation	X[1]	Daily @ 0800 ± 1h	Every 7 days@ 0800 ± 1h	
PEEP/FiO_2		Daily - Patient Record	Daily - Patient Record	
Fluid Balance[2,Ꞙ]		Daily	Days 14, 21, 28	
Adverse Event (AE) assessment		Daily	ADI [Ꞙ]	
CBC	X	Daily (2x)	Every 7 days	
Blood chemistries (Chem 20)	X	Daily (2x)	Every 7 days	
Immune System CD4 Cell Activation		Days 1, 3, 5, 7	14 and Discharge	
Urine ß-hCG in pre-menopausal females	X			
Pressor agent use[Ꞙ]	Day -1	Daily	Every 7 days	
Drug/Hormone Removal[&&]		Day 2	NA	
HIT/D Dimer (sites as specified)		Day 1 and 7	NA	
Mortality		Daily	Daily	X
Ventilation days following selection as control [Ꞙ]			X	

ADI: As determined by intensivist

[1]Ventilation: Subject must be intubated and PaO_2/FiO_2 <300mm Hg for ARDS/ALI. Ventilation and weaning from the ventilator guidelines from ARDS Net supplied, but your hospital ventilation guidelines may differ and should be followed

[2 Ꞙ]Fluid Balance = total intake – total output

&& From medication list for which an assay exists for those subjects on dialysis only – archive samples will be utilized

Ꞙ From hospital records

ꞘꞘ From hospital records except for β-hCG

∂ As described in section 3.3.2

[3]Calculated post study

PROTOCOL

1 STUDY OBJECTIVES

1.1 Objectives

The study objectives are to confirm the safety and effectiveness of CytoSorb. Specifically:

Primary Objective: Subjects receiving hemoperfusion with the CytoSorb device in combination with the standard of care for the treatment of acute respiratory distress syndrome or acute lung injury in the setting of sepsis will have lower relative IL-6 levels than control subjects receiving only the standard of care.

Secondary Objective: Measure the effect of treatment on key physiologic indicators of health

1.2 Background CytoSorb Device

MedaSorb Technologies, Inc. has developed a novel polymeric bead capable of removing cytokines from blood that is both highly adsorptive and biocompatible. The polymer is packed into a cartridge which constitutes the CytoSorb hemoperfusion device. The CytoSorb hemoperfusion device is intended for use as an adjunct to the standard of care in subjects with acute respiratory distress syndrome (ARDS) or acute lung injury (ALI), in the setting of sepsis. The device is designed to be used in a stand alone configuration or in combination with hemodialysis. The device is designed for use in a standard, commercially available hemodialysis circuit with a blood pump or hemodialysis machine.

MedaSorb polymer was originally developed for the removal of Beta-2 microglobulin (β2M) and labeled BetaSorb. The BetaSorb device was intended to supplement hemodialysis which is ineffective in removal of β2M. In long-standing cases of DRA, amyloid deposits develop within visceral organs and can cause heart failure, gastrointestinal bleeding, bowel perforation, infarction, and obstruction and chronic diarrhea. Amyloid deposits can significantly impair the function of these vital organs and complications can be fatal.

During the development of the BetaSorb product, MedaSorb discovered the potential use of the hemoperfusion device in the removal of cytokines indicated in the systemic inflammatory response syndrome (SIRS) associated with sepsis. Given the unmet medical need of therapies to address sepsis, MedaSorb engaged University of Pennsylvania to conduct experimentation using a septic rat model to test the concept of using the MedaSorb polymer in a hemoperfusion device to remove cytokines. Those original studies showed a statistically significant improvement in survival of treated animals versus untreated control animals.[1] Based on these data, MedaSorb Technologies decided to pursue the new indication in the removal of cytokines in the treatment of sepsis and renamed the product CytoSorb to reflect the new indication.

[1] Kellum JA, Song M, Venkataraman R. Hemoadsorption Removes TNF, IL-6 and IL-10, Reduces NFB DNA binding and Improves Short-Term Survival in Lethal Endotoxemia. Crit Care Med 2004;32, 801-805

Much of the original test data, such as ISO 10993 testing, was on the product when its' trade name was BetaSorb. BetaSorb and CytoSorb are **exactly** the same device only the label and indication for use differ. For purposes of traceability and clarity, testing data developed on the product when it was called BetaSorb (i.e. ISO 10993 data) will continue to be referred to as BetaSorb.

For Further Detail Concerning Prior Clinical Experience and Clinical Safety Data See Investigators Brochure

1.3 Prior Clinical Experience in Septic Application

Human Experience – Emergency and Compassionate Use Treatment of a Septic Subject

Case Report – BetaSorb in Septic Shock Subject (Investigator: Kenneth Kleinman, MD, Encino, CA - USA)

The subject was a 74 year-old male with a greater than 20 year history of polycystic kidney disease. He had begun renal replacement therapy approximately 17 years before the current illness, with two unsuccessful short-lived kidney transplants and a period of peritoneal dialysis intervening in his long course of hemodialysis. He was also known to have severe polycystic liver disease.

He was admitted to the hospital complaining of right-sided abdominal pain associated with nausea for four days. The differential diagnosis included cholecystitis versus polycystic liver with an infected cyst/abscess. Magnetic resonance imaging revealed large hepatic cystic masses. Cholescintigraphy was negative. Initial treatment included empiric antibiotic coverage (piperacillin/tazobactam). By day 5 after admission he developed hypotension, encephalopathy and multiorgan failure, with lethargy and decreased breath sounds. A nasogastric continuous suction tube was placed, and hemodialysis with intradialytic hyperalimentation was performed on nearly a daily basis (see fig 1). Multiple cultures from each of the first 4 days of admission were negative. On day 5 a blood culture grew Pneumatosis intestinalis and was treated by adding metronidazole to piperacillin/tazobactam. The possibility of Clostridium difficile associated diarrhea was raised. He was begun on intravenous norepinephrine at 2-3 μg/min for hypotension. By the 6th hospital day his white count had increased to 22,000/mm^3, his hematocrit had dropped to 32%, and platelets were 70,000/ mm^3. On day 7 he had evidence of free air in what was felt to be right-sided Pneumatosis intestinalis. Laparotomy and subsequent pathology confirmed the diagnosis, and revealed an associated right paracolic phlegmon mass with free air but without leakage of intestinal contents. He underwent a diverting ileostomy. Postoperatively he was continued on antibiotics, intensive pulmonary care with intubation and ventilation. On day 8 vancomycin was added to the other antibiotics (with therapeutic levels). On day 9 his white blood cell count had come down to 13,900/ mm^3. Additional medications included erythropoietin with dialysis, digoxin, and norepinephrine for blood pressure support and ondansetron as needed. On clinical examination the right paracolic area was found to be full.

At this point his condition was so critical that it was decided to use an experimental sorbent device shown in preclinical studies to remove inflammatory cytokines implicated in sepsis/SIRS. FDA was contacted by the manufacturer and gave permission for compassionate use of the device. Institutional Review Board permission and the family's informed consent were obtained, after which he was treated for 3 1/2 hours with a 300 ml sorbent device (BetaSorb, MedaSorb Technologies, Inc, Monmouth Junction, NJ) placed in series with a high flux hemodialyzer. Additional heparin dosage was monitored by activated clotting times. Blood pressure support was discontinued after the first treatment.

He was treated 5 times over the next 8 days and then intermittently 7 times over the ensuing 13 days for a total of 12 times. Cytokines including CRP were measured during several combined treatments.

In the first 13 days of hospitalization he had intermittent fever, leukocytosis, mild thrombocytopenia, and mild hyperbilirubinemia. Blood cultures from day 4 revealed Staphylococcus hominis bacteremia, and metronidazole was discontinued. Vancomycin levels were maintained in the therapeutic range.

Sputum culture continued to be positive for Candida albicans, but all drains were negative for aerobic and anaerobic organisms. He was extubated on day 14 in stable condition, and a beta blocker was added because of intermittent arrhythmias.

Chest x-ray on day 14 was suggestive of basilar atelectasis/infiltrate or congestive heart failure, when his white count had fallen to 15,300/mm3, the platelet count was 65, 000/mm3, the sedimentation rate was 120mm/hr, LDH was 218 u, bilirubin 3.8 mg/dl, alkaline phosphatase 119 units, and D-dimer was negative for thrombosis.

Antibiotics were changed to Levaquin and Flagyl. Vancomycin and fluconazole were continued. Because of persistent fever, abnormal liver function, thrombocytopenia, leukocytosis, continued S. hominis positive blood cultures, and encephalopathy, and hypotension on day 18, he had surgical re-exploration on hospital day 19.

A 400 to 500 ml right flank retroperitoneal abscess and necrotic ileostomy were found, and the abscess was drained via three Jackson-Pratt drains. Mitomycin and gentamicin were added after Gram negative organisms were cultured from the Jackson-Pratt drains. He required ventilator support, pressor support and because of worsening arrhythmias was started on amiodarone. Antibiotics were changed; levaquin was discontinued, vancomycin, Flagyl, amikacin were continued and he was started on Cancidas for potential fungal infection.

Over the next 11 days he had high fevers to 104 F, leukocytosis, thrombocytopenia, positive blood cultures for E Coli, enterococcus fecalis, Candida albicans, deteriorating liver function, and encephalopathy. A positive white blood cell scan revealed a collection in the right upper quadrant.

At this point he was in extremis with poor nutrition, worsening liver function, ongoing arrhythmias, hypotension, respiratory failure as well as increased encephalopathy.

On day 30 surgical exploration revealed a retroperitoneal with a perforated right ascending colon, requiring right colostomy. He continued to do poorly although he was treated intensively with dialysis, combined

BetaSorb/dialysis, ventilation, pressor support and antibiotics. Over the next 10 days he continued to have intermittent bacteremia (E.coli, streptococcal species, enterococcus fecalis, staphylococcus), fever, leukocytosis, hyperbilirubinemia (total 24.2 mg/dl), thrombocytopenia, despite continued antibiotics. He developed a right perihilar infiltrate, abdominal distension with skin breakdown around the colostomy, hypotension and arrhythmias all consistent with sepsis/systemic inflammatory response syndrome.

The case was discussed at length with the patient's family, further surgical procedures were considered futile and treatment was withdrawn. He died on the 40th hospital day.

Summary of Treatments Administered and Data Collected

A total of 12 BetaSorb (CytoSorb) treatments combined with hemodialysis were performed. Cytokine changes associated with BetaSorb (CytoSorb) treatment are shown graphically below Figure CSP. We have also included Table CSP gives selected data for chemistries, WBC, platelets, and cytokines measured in the septic subject; full clinical chemistries etc. can be found in Appendix H.

Figure CSP. California Septic Patient changes in Inflammatory Markers Measured Under an Emergency Use Approval

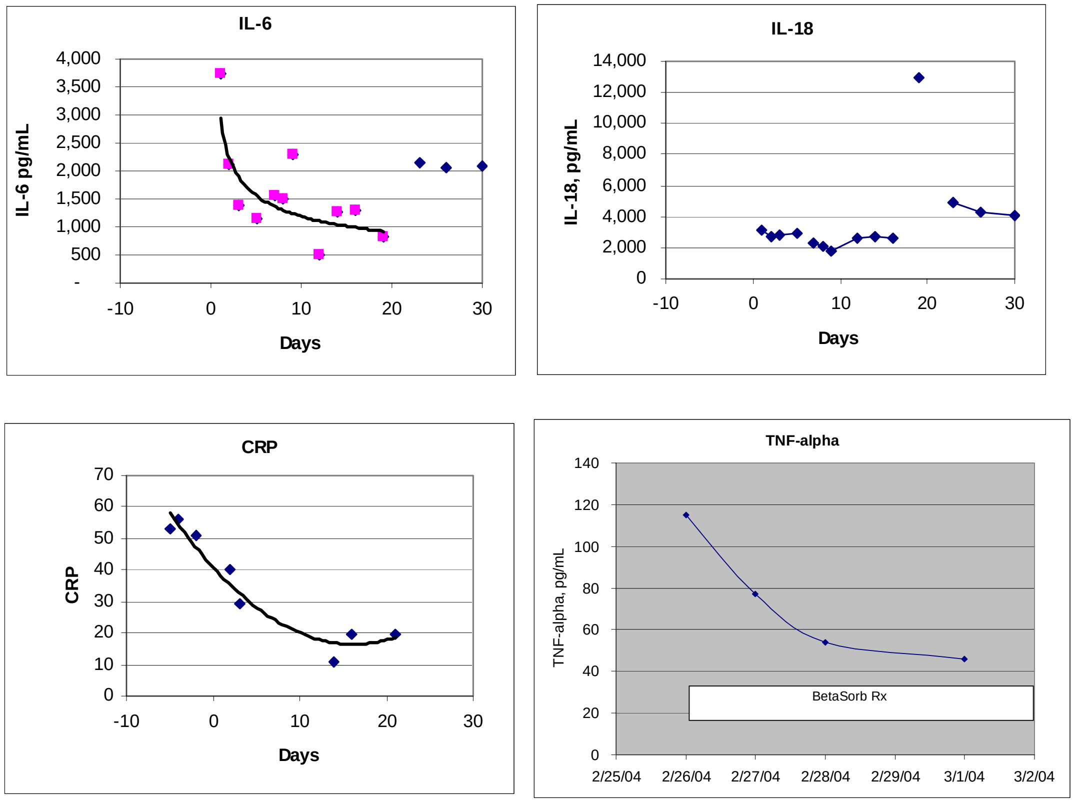

Table CSP. Select Data from California Septic Patient

Day	1	2	3	4	5	8	9	12	14	16	19	21
WBC count	14	14	15	11	18		12		15	19		17
Pretreatment platelet count	73	68	72	60	66		56		102	72		41
IL-6	3731	2120	1379		1157	1505	2288	504	1279	1281	830	
IL-18	3183	2743	2815		2881	2053	1768	2589	2732	2573	13003	
CRP		40	29						11	20		20
TNF	122	64	54	42								

Treatment days: 2/26//04 – 3/1/04, 3/4/04, 3/5/04, 3/8/04, 3/10/04, 3/12/04, 3/15/04, 3/17/04

WBC= white blood cell count (normal 4.3-10.8 x $10^3/mm^3$), platelets=platelet count(normal 150-400 x $10^3/mm^3$), IL=interleukin (IL-6 normal up to 4.3 pg/mL, IL-18 normal 50-565 pg/mL), CRP= C-reactive protein (normal < 10 mg/L), TNF = Tumor Necrosis Factor (normal <5 pg/ml)

Conclusion

Treatment with BetaSorb (CytoSorb) was initially associated with a recovery of blood pressure and improvement in ventilator status. The improvement was the impetus for the trial proposed in this IDE supplement. MedaSorb makes no claims that BetaSorb (CytoSorb) was of proven efficacy in the treated subject, but did suggest that an investigation into efficacy directed to improvement in lung function should be carried out.

2 INVESTIGATIONAL PLAN

2.1 Study Design:

Once Informed Consent (IC) and authorization to use the Protected Health Information (PHI) is obtained, subjects will be screened, randomized to control or treatment groups and enrolled into the clinical study. Subjects in the treatment group will be treated with a 6 hour hemoperfusion alone, daily for up to 7 days or successful weaning from the ventilator which ever comes first. Should subjects require hemodialysis CytoSorb will be placed in line in the dialysis circuit for a duration of 6 hours. This is referred to as “Combined Treatment”. Each treatment session will be referred to as a “combined treatment session”. CytoSorb will be placed in series with the dialyzer in the dialysis circuit, upstream of the dialyzer during Combined Treatment. A given subject will be treated with the same membrane type of dialyzer throughout the study. The dialyzers will be available commercially.

2.2 Risk Analysis

2.2.1 Risks of Study Procedures

The risk profile associated with the CytoSorb is expected to be minimal and consistent with other treatments currently in clinical use such as dialysis. These risks include:

- Chills
- Bleeding in relation to anticoagulation
- Hypotension
- Removal of drugs and hormones
- Allergic response to device materials
- Reduction in platelet count
- Reduction in white blood cell count
- Reduction in proteins (albumin, total protein)
- Coagulation within device
- Heparin induced thrombocytopenia (HIT)
- Drug Removal (i.e. antibiotics, pressor agents, etc)

2.2.2 Potential Benefits to Subject

Potential benefits of using the investigational device are not known at this time. It is anticipated that the use of the CytoSorb device would result in potential benefits that include a decrease in the use of a ventilator and the following;

- Potentially increased ventilation free days (decrease in days on ventilation), and observation of minimal ventilator support ($FiO_2 \leq 40\%$ and PEEP ≤ 5 cm H_20)
- Possible decrease in hospital stay, including ICU
- Possible improvement in 28 and 60 day mortality
- Potential decrease in progression of organ failure (MODS score)
- Potentially fewer ancillary treatments such as dialysis
- Possible reduction in pressor use
- Potential decrease in SOFA and APACHE II scores

2.2.3 Minimization of Risks

To evaluate device related risk, a risk analysis was performed in accordance with the harmonized European standard ISO 14971, "Medical Devices – Risk Analysis". The results of the analysis indicate that all potential device-related hazards have been reduced to an acceptable level. Mechanical and performance concerns have been reduced by extensive animal and bench testing. The device materials have been shown to be biocompatible and acceptable for human use through ISO10993 testing and prior clinical trial of the device known as BetaSorb for removal of beta-2 microglobulin in end stage renal disease subjects. The MedaSorb CytoSorb device is believed to pose little or no additional risks above other commercialized blood filter and dialysis devices.

All attempts will be made to minimize the clinical risks associated with the procedure. Inclusion and exclusion criteria have been written to eliminate subjects thought to be a medical "high risk" for this therapy. Adherence to protocol will be stressed by MedaSorb personnel or their representative in attendance at the procedures. As with hemodialysis, subjects are dosed with their medications following treatment to assure that a given drug is sufficiently retained. All subjects will be monitored for blood levels of albumin, platelets and white blood cells, and treatment will be discontinued for a given subject if either the investigator or MedaSorb believe that the change in blood values places the subject at risk.

Post procedure management with proper anticoagulation and drug and assessment of subject's status will be emphasized in the physician training. Selecting investigators with experience in use of extracorporeal therapy, good clinical practices and training them to properly use the CytoSorb device will mitigate risk associated with use of the device and procedure related adverse events. Subject follow up will be comprehensive and consistent to avoid unnoticed delayed adverse events.

The CytoSorb device may be capable of removing drugs, i.e. antibiotics, pressor agents, etc. similar to dialysis. The physician is advised to measure concomitant drug concentrations, where a test exists, post CytoSorb treatment and adjust drug doses accordingly.

2.2.4 Justification for the Study

The investigation is justified by the potential for the device to remove significant amounts of cytokines which are not addressed through the current standard of care for ARDS or ALI. Effective removal of these toxins may contribute to decreased ventilation time through modulation of the systemic inflammatory response. This modulation of the systemic inflammatory response may provide the standards of care for ARDS or ALI an opportunity for greater impact on subject health and ultimately a better outcome.

2.2.5 Description of the Subject Population

This trial will be an open study. Up to a total of 100 subjects will be enrolled in order to collect complete data sets on 30 subjects that receive CytoSorb treatment and 30 control subjects that do not receive CytoSorb treatment. Subjects who meet all of the inclusion and none of the exclusion criteria in the study protocol will be eligible.

2.3 Selection of Study Population

2.3.1 Inclusion Criteria

- Signed informed consent document (ICD)
- Male or female ≥ 18 and ≤ 80 years of age.
- Subjects must have diagnosis of ARDS or ALI, based on ARDSNet Definition, established within last 72 hours, confirmed by clinical, radiological, or physiologic findings [1]
- Subject must be intubated
- ≤ 3 days on a ventilator prior to enrollment
- Subjects must have confirmed diagnosis of sepsis [2]
- Subject must have had at least 24 hours of antibiotic therapy
- Pre-menopausal female subjects must have negative pregnancy test.
- Subject must be available for periodic blood sampling, study related assessments, and management at the treating institution for the duration of the study. Subject must have permanent home address to allow completion of 60 day follow-up
- Subject or health care proxy has the ability to understand and willingness to sign the informed consent form.

2.3.2 Exclusion Criteria

- Currently participating in another clinical study involving investigational chemical compound, biologic, or device within the last 30 days prior to the start of this trial.
- ARDS Net Exclusion Criteria
    - Neuromuscular disease that impairs the ability to ventilate spontaneously, such as C_5 or higher spinal cord injury, amyotrophic lateral sclerosis, Guillain-Barré syndrome and myasthenia gravis.
    - Increased intracranial pressure, tricyclic antidepressant overdose, hemoglobin SS, hemoglobin SC or other conditions where hypercapnia would be contraindicated.
    - Severe chronic respiratory disease including hospitalization within last 6 months for respiratory failure.
    - Morbid obesity (Body Mass Index ≥40 kg/m^2).
    - Burns > 30% BSA, bone marrow transplant, lung transplant or end stage hepatic liver failure.
- Subject with mean arterial pressure ≤ 60 mmHg regardless of use of pressor agents.

---

[1] ARDS Net Definition: **Bilateral abnormalities** on chest X-Ray, No evidence of congestive heart failure, pulmonary capillary wedge pressure ≤ 18 mm Hg (if available), P/F ratio ≤ 300 mm Hg, Mechanical ventilation required. **Bilateral abnormality: for purposes of the sepsis trial the chest Xray reading of the treating physician or PI at the time of enrollment is considered the definitive reading. "Bilateral abnormality" of the lungs is the criterion for inclusion. For example, a patient who has lobar pneumonia or unilateral chest trauma AND abnormality in the contralateral lung can meet the ALI_ARDS definition**

[2] **Sepsis:** SIRS plus known or suspected infection source, blood cultures need not be positive, bacteremia need not be present. **Systemic Inflammatory Response Syndrome (SIRS):** Subject presents with two or more of the following criteria - heart rate > 90 beats per minute, body temperature < 36 or > 38°C, respiratory rate > 20 breaths per minute or, P_aCO_2 < 32 mm Hg, white blood count < 4 x 10^9 or > 12 x 10^9 cells/L), or the presence of greater than 10% immature neutrophils.
The diagnosis of infection may be made with cultures and Gram stains of biological fluids, pathological and surgical specimens, or radiology procedures.

- Subject with active malignancy receiving chemotherapy or radiation treatment within last 60 days.
- Subjects with AIDS, CD4 count of < 200 or 14%, or the presence of an AIDS defining illness (HIV+ subjects may be enrolled)
- Subject with acute coronary syndrome.
- Subjects with decompensated heart failure with New York Heart Association (NYHA) classification IV
- Subjects with Chronic Kidney Disease (CKD) stage 5 or greater will be excluded
- Subjects with end stage hepatic liver failure
- Subjects on immunosuppressive agents, excluding corticosteroids
- Platelets $\leq 20{,}000/mm^3$
- Subjects on anti-TNF therapy
- Subjects about to receive or receiving drotrecogin alpha (Xigris) therapy
- Subject is pregnant or breastfeeding.
- Subject has a known allergy to any component of the CytoSorb hemoperfusion device
- Subject has any active disease condition that could limit compliance with the study procedure, including but not limited to the following: acute coronary syndrome, life-threatening cardiac arrhythmia, or psychiatric or social conditions, considered by investigator(s) to preclude successful completion of the study.

2.4 Endpoint Analysis

2.4.1 Analysis

A large number of endpoints are planned for collection to give a broad assessment of the clinical utility of CytoSorb. Endpoints are separated into three levels (primary, secondary, exploratory) based upon their perceived relevance for an eventual pivotal trial for the demonstration of device safety and effectiveness.

Primary Endpoint:

Relative IL-6 levels as a percent (%) of baseline will be lower in subjects receiving CytoSorb treatment in conjunction with the standard of care as compared to control subjects receiving only the standard of care for ARDS/ALI in the setting of sepsis.

Secondary Endpoints:

- Ventilation Support: This is defined as the number of days, between day 1 and day 28 where the subject was:
  - off ventilator (ventilator free days) or
  - $FiO_2 \leq 40\%$ and PEEP ≤ 5 cm H_2O
- Reduction cytokines TNF-α, IL-1b, IL-10, CRP
- 28-day all cause mortality
- Oxygen Index (OI)
- P/F ratios
- MODS scores

Exploratory Endpoints:

- SOFA Score
- APACHE II score
- 60 day all cause mortality
- Vasopressor use
- Immune system CD4 cell activation

2.4.2 Sample Size

This is a registration study to gather data on the impact of CytoSorb treatment on cytokine clearance. Result from this trial will be used to support a submission for CE mark. Up to a total of 100 subjects will be enrolled in order to collect complete data sets on 30 subjects that receive CytoSorb treatment and 30 control subjects that do not receive CytoSorb treatment.

2.4.3 Study Population

This is an open label, two-arm, prospective randomized study. Subjects will be randomized in a near 1:1 fashion until either of the following are met: either 30 treatment and 30 control subjects have completed their 28-day follow-up, or 100 subjects have been enrolled. Falling short of the 30/30 target due to subject deaths or other loss to follow-up will not be considered a protocol deviation.

3 ADMINISTRATIVE AND TREATMENT PROCEDURES

3.1 Administrative Procedures

3.1.1 Informed Consent Document Form

The Patient Informed Consent Document (ICD) must be signed by all study subjects prior to study participation. In case the patient is not capable to give his written consent, the consent form must be dated and signed by the subject's legally authorized representative (the legal healthcare proxy). For more information on the consenting process, please refer to page **43** , paragraph **7.3** of the protocol.
The original signed ICD will be retained in the subject's study records and a copy of each document will be provided to the subject. A sample of Inform Consent document (Patient Inform Consent, Legal health proxy as well as Independent Physician Consent Form) is located in Appendix 1 of this protocol.

3.1.2 Source Document Binder Contents

The following documents are to be retained in the Source Document Binder:

- a copy of the screening history and physical examination
- a copy of the treatment flow sheet for each treatment session
- adverse event information
- lab work at specified intervals during the study, and
- any other documentation used to complete the CRFs.

3.1.3 Equipment Calibration

To ensure the accuracy and the consistency of measurements, all sites are required to carry out routine preventative maintenance and calibration of the equipment used in the study, within 30 days of the start of the study, and to complete all on-going preventative maintenance, as recommended by the machine manufacturer. A copy of the user manual for all machines used in the study and copies of the maintenance records for those machines must kept in the Sites' Study Regulatory Binder.

3.2 Treatment Procedures

3.2.1 Screening Evaluation (Day -1, -2, -3)

- Informed Consent
- Inclusion/Exclusion criteria
- Medical history, and physical examination
- Demographic data, including subject age, gender, measured height and weight
- Etiology of ARDS/ALI
- Co-morbid conditions and concomitant medications
- Complete Blood Count
- WBC (including differential)
- Hematocrit
- Platelets
- Beta hCG (human chorionic gonadotropin) for premenopausal females
- Chest X-ray
- Chemistry panel
- Vassopressor data, Day – 1

• Albumin	• Chloride
• Alkaline phosphatase	• Cholesterol
• ALT	• Gamma-GT
• AST	• Glucose
• Bilirubin, Total	• Lactate Dehydrogenase
• Bilirubin, Direct	• Potassium
• Urea	• Phosphate
• Calcium (total)	• Sodium
• CO_2	• Total Protein
• Creatinine	• Uric Acid

3.2.2 Hemoperfusion Treatment (Day 1-7)

Treatment subjects will undergo hemoperfusion daily with a single use CytoSorb device for 6 hours, using temporary vascular access (catheters). The patient will receive CytoSorb treatment for up to seven (7) consecutive days or successful weaning off the ventilator (48 hours of unassisted breathing), which ever comes first. Targeted flow rate for therapy is 250 - 400 ml/min.`

Prior to starting the hemoperfusion session, the investigator(s) or their representatives will perform the following:

Record and verify the Lot # and the Reference # of CytoSorb that is being used (identification numbers located on the device). Prior to opening the CytoSorb device package, the protective pouch and the device will be inspected for damage. If damage or leaks (e.g. presence of moisture on the exterior surface of the device) are observed, the device will not be used, but will be returned to MedaSorb for inspection.

The following will be recorded on the CRFs

- Blood flow rate from blood pump
- Total blood volume processed
- Duration of the treatment session
- Start, end-hemoperfusion vital signs
- Pulse rate
- Blood pressure (Systolic/Diastolic and Mean Arterial Pressure)
- Aural temperature
- Arterial and venous line pressures
- Heparin prescription and dosing or ionized calcium
- Concomitant medications – including intra- & extra-hemoperfusion

Within the first 5 minutes of starting treatment, a “start” blood pressure and pulse will be measured and recorded. Vital signs will be recorded at specified time points on the CRFs. If additional vital signs are taken during the treatment, they must also be recorded on the CRFs. The "end" of the treatment is defined as the end of the prescribed treatment time, before the blood in the extracorporeal circuit is returned. The “end” blood pressure and pulse are taken at that point. The “post” blood pressure and pulse are taken once the blood has been returned.

Should treatment combined with dialysis be necessary in the course of the illness then the following will be recorded. The dialyzers will be available commercially.

- Dialyzer manufacturer and model, as well as machine manufacturer and model
- Blood flow rate
- Dialysate flow rate
- Duration of the treatment session (start and stop time)
- Net ultrafiltration volume
- The total blood volume processed
- Pre-, start, during, end- and post-treatment vital signs
- Pulse rate
- Blood pressure
- Body weight (measured pre- and post-treatment if available)
- Arterial and venous line pressures
- Heparin prescription and dosing or serum ionized calcium
- Concomitant medications
- Adverse events

The pre-combined treatment vital signs and a physical assessment will be recorded prior to the start of the treatment. Within the first 5 minutes of starting treatment, a “start” blood pressure and pulse will be measured and recorded. Vital signs will be recorded at specified time points on the CRFs. If additional vital signs are taken during the treatment, they must also be recorded on the CRFs. The "end" of the treatment is defined as the end of the prescribed combined treatment time, before the blood in the extracorporeal circuit is returned. The “end” combined treatment blood pressure and pulse is taken at that point. The “post” combined treatment blood pressure and pulse are taken once the blood has been returned.

Subjects must be systemically heparinized or regionally anticoagulated with citrate prior to all treatment sessions. Clotting times will be monitored using Partial Thromboplastin Time (aPTT), Activated Clotting Time or serum ionized calcium as discussed in section 3.3.3. To establish the heparin regimen and clotting times for each subject, aPTT measurements.

3.2.2.1 Blood Sampling

T_0 blood sample should be taken 5 minutes after treatment starts to assure saline is cleared from lines. T_{360} sample is taken at the end of treatment.

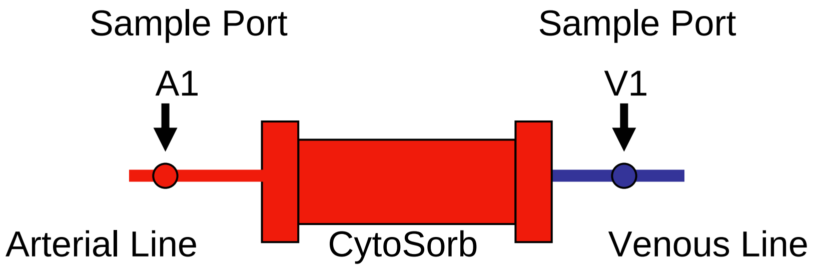

Figure 1. Locations of blood sampling during hemoperfusion treatment.

3.2.3 Combined Treatment and Dialysis Alone (Control Subject)

Prior to starting the combined treatment session, the investigator(s) or their representatives will perform the following:

Combined Treatment

Record and verify the Lot # and the Reference # of CytoSorb that is being used (identification numbers located on the device). Prior to opening the CytoSorb device package, the protective pouch and the device will be inspected for damage. If damage or leaks (e.g. presence of moisture on the exterior surface of the device) are observed, the device will not be used, but will be returned

to MedaSorb for inspection.

In all combined treatment sessions, CytoSorb will be placed upstream of (before) the HFD as shown in Figure 2. Attach CytoSorb to the dialysis circuit in the manner described in the CytoSorb Instructions for Use (IFU). The entire circuit will be primed and rinsed with no less than 2.0 L of normal saline, following the dialyzer and CytoSorb manufacturer IFU. During treatment with CytoSorb, subjects may report a feeling of coldness or chills associated with the additional extracorporeal blood volume added by the hemoadsorption device. The investigator(s) may increase the subject's routine dialysate temperature. This adjustment historically has relieved feelings of coldness or chills.

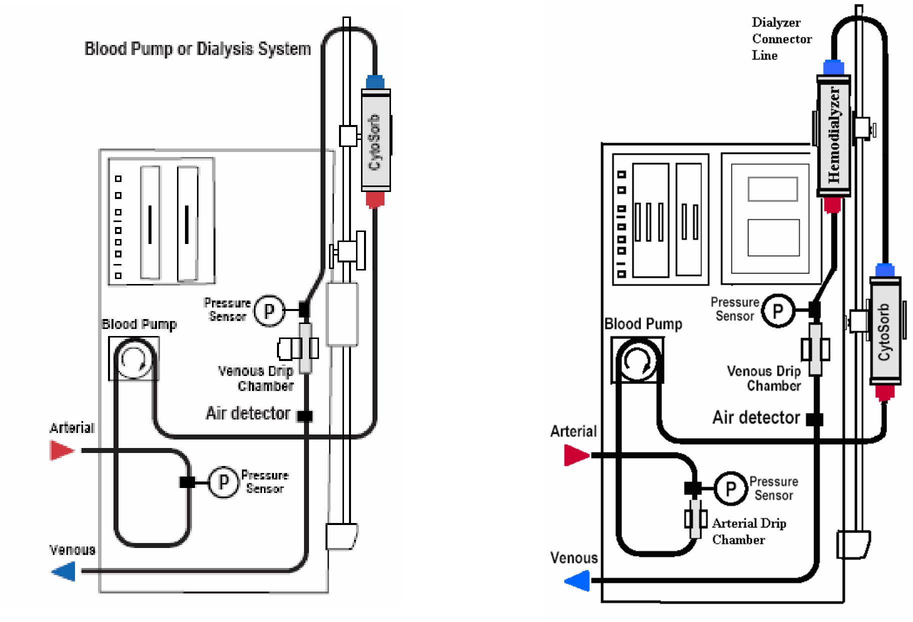

Figure 2. Schematic diagrams showing CytoSorb alone on blood pump and stand with attached blood lines and with blood lines set up to record arterial and venous line pressure measurements (left panel), or CytoSorb upstream of (before) the high flux hemodialyzer with blood lines set up to record arterial and venous line pressure measurements (right panel). Heparin will be administered into the venous line.

Pre-treatment vital signs and physical assessment will be recorded prior to the start of each treatment. Within the first 5 minutes of initiating the treatment, a "start" blood pressure and pulse will be measured. Vital signs also will be recorded at separate specified time points on the CRFs. Additional vital signs, if taken, will be recorded on the CRFs. During each treatment session, any changes made to the ultrafiltration rate and the dialysate composition will be documented on the appropriate CRF. The "end" of the treatment is defined as the end of the prescribed dialysis time, before the blood in the extracorporeal circuit is returned. The "end" treatment blood pressure and pulse are taken at that point. The "post"-treatment blood pressure and pulse will be taken after the extracorporeal blood volume has been returned to the subject.

The same subject prescription parameters will be used for each combined treatment session for a given subject unless otherwise directed by the PI to protect the safety and welfare of the subject. The following parameters will be collected at each combined treatment session:

- Dialyzer Manufacturer and Model, and machine manufacturer and model
- Device inspection
- Blood flow rate
- Dialysate flow rate

- Duration of the dialysis session (start and stop time of dialysis) and actual dialysis treatment time (machine derived)
- Dialysate temperature throughout the entire treatment session
- Amount of net ultrafiltration
- Total blood volume processed
- Pre-, start, during, end- and post- dialysis vital signs
- Pulse
- Blood pressure
- Oral temperature (pre- and post-dialysis)
- Body weight (measured; pre- and post-treatment if available)
- Arterial and venous line pressures
- Heparin prescription and dosing or serum ionized calcium
- Concomitant medications – including intra- & extra-treatment medications
- Adverse events (SAEs)

Throughout the treatment session, monitoring the CytoSorb device for blood leaks or clots is essential. Any adverse device events must be recorded on the appropriate CRFs, by indicating the location of leaks or by recording the size and the visual appearance of clots present in the device on the schematic diagrams provided on the appropriate CRF.

BloodSampling

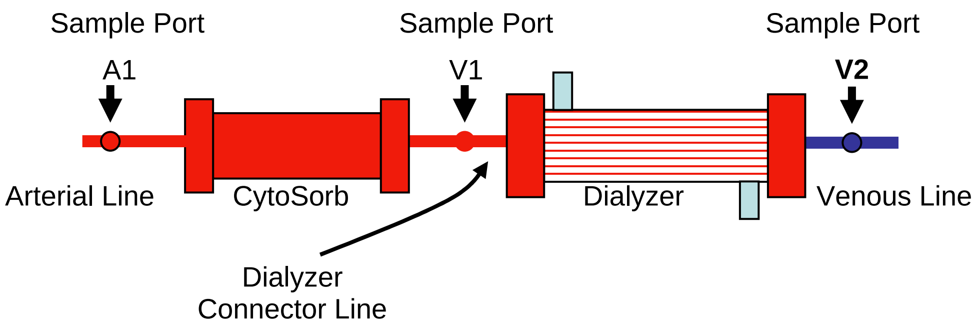

Figure 3. Locations of blood sampling for the Combined Treatment

Dialysis Alone (Control Subjects)
Some subjects that are part of the control group may require dialysis. Those subjects requiring dialysis will have the same prescription parameters recorded as the combined treatment session.

3.2.4 Post Treatment Drug Dosing

The CytoSorb device may be capable of removing drugs, i.e. antibiotics, pressor agents, etc. similar to dialysis. The physician is advised to measure concomitant drug concentrations, where a test exists, after CytoSorb treatment and adjust drug doses accordingly. In addition, when nutritional supplementation is indicated, the physician is encouraged to administer gastric or other enteral tube feeding rather than total parenteral intravenous nutrition (TPN) and lipids. Lipid or fat emulsions may negatively affect the CytoSorb device. If lipids (eg. Lipovena), fat emulsions or TPN containing lipids are required or clinically indicated, however, then the physician is advised to administer these after CytoSorb treatment is completed or discontinue administration two (2) hours prior to the next CytoSorb treatment.

3.2.5 Ventilation and Weaning

The investigators participating in this trial are encouraged to follow the ARDSNet Ventilation and Weaning guidelines as outlined in Appendix 4. However, patient condition or hospital policy may require different ventilation guidelines than those outlined.

3.2.5.1 Ventilation Modes

Subjects are required to be intubated and on mechanical ventilation when enrolled in the study. Typical methods of ventilation are pressure or volume controlled ventilation. Patients on Non Invasive Positive Pressure Ventilation (NIPPV) will be excluded from enrollment. For purposes of clarification, if a subject is extubated and successfully weaned but then relapses and is placed on NIPPV, this is considered mechanical ventilation and would be recorded accordingly.

3.2.5.2 Definition and Method of PaO_2/FiO_2 (P/F) Ratio Measurement

For the purposes of this trial we are defining the PaO_2/FiO_2 ratio as PaO_2 and FiO_2 at the time of measurement. The FiO_2 **should not** be adjusted to 100%.

3.2.6 Glasgow Coma Scale Scoring

The Glasgow Coma Score (GCS) is used in the determination of MODS, APACHE II and SOFA scoring. The scoring systems assess eye, verbal and motor responses. There are special considerations when assessing GCS for the patient on a ventilator; verbal response is not possible under normal ventilation conditions. The patient should be on a "drug holiday from sedation" during assessment. GCS is typically reported as Eyes (usually 4), Verbal (T) since in cannot be measured in an intubated patient and Motor (usually 6) for a score of 10T in a patient who is intubated but alert.

3.3 Efficacy Evaluation

3.3.1 Intra-Session Reduction Studies

Intra-session percent reductions of cytokines will be assessed by measuring the concentration differences between a pre-treatment sample drawn from the catheter access before administration of saline or heparin (T_0), and a sample drawn from the arterial (A1) line at the end of treatment (T_{360}).

3.3.2 Cytokine/Drug/Hormone Kinetic Analysis

Clearance and total quantity of solute will be assessed on day 2 of the treatment period for all treated subjects in the following manner:

Clearance for Cytokine/Drug/Hormone and quantity of solute removed at the specific treatment sessions will be calculated from blood samples. Blood pump speed will serve as the nominal blood flow rate (Qb). There will be five sample times for a given session: Start of session (T_0) Cytokine/Drug/Hormone, 15 minutes into start of session (T_{15}) Cytokine/Drug/Hormone, 60 minutes after start of the treatment session (T_{60}) cytokine only, the fourth sample 180 minutes after the start of the treatment session (T_{180}) cytokine only, and the fifth at the end of the treatment session (T_{360}) Cytokine/Drug/Hormone. The measured amount of solute removed at these different time points will be used to calculate the total amount of solute removed during the treatment session.

Blood sampling for measurement of cytokines for CytoSorb and CytoSorb/dialysis combined treatments will be taken from the extracorporeal circuit. During the CytoSorb hemoperfusion session, arterial (A1) and venous (V1) samples will be drawn (Figure 1). During any Combined Treatment Session, samples from the arterial (A1) and venous (V1) lines will be the only samples taken, to calculate cytokine clearance across the device.

Control subjects will provide a T_0 and T_{360} sample to measure natural clearance of drugs and hormones on day 2.

In most cases Control Subjects will not have an access for blood sampling. Control subjects will provide blood samples via needle stick..

3.3.3 Heparinization and Regional Citrate Anticoagulation

3.3.3 .1 Heparinization

If the patient has a history of testing HIT antibodies, do not use heparin as the anticoagulation method, use citrate regional anticoagulation in combination with continuous veno-venous hemofiltration (CVVH) or dialysis; see section 3.3.3.2. If heparin is chosen as the anti-coagulant of choice, subjects must be systemically and adequately heparinized BEFORE starting all treatment sessions to prevent fouling of the CytoSorb device. Patients should reach a documented aPTT of 60-80 seconds or an ACT of ~ 160 seconds to 210 seconds before beginning blood perfusion through the CytoSorb device. Please collect this data and record in the space provided in the CytoSorb Treatment CRF.

	T_0	T_{30}	T_{180}	T_{360}	As required
aPTT or ACT					

Clotting times will be monitored using aPTT or ACT. The same aPTT measurement tool will be used throughout the study for a given subject. The heparin dose will be adjusted as necessary to maintain an adequate amount of systemic anti-coagulation, with an aPTT target of 60-80 or an ACT of ~ 160 seconds to 210 seconds. Heparin will be administered through the venous line.

Using heparin data derived from a study of an identical device in end-stage renal disease subjects during hemodialysis alone and during combined hemodialysis/hemoperfusion a very small increase in heparin was required to maintain optimal activated clotting times (ACT) of ~ 160 seconds to 210 seconds during combined treatment. Because of the variability in heparin requirements, heparin bolus will be 30 – 50 units/Kg bolus and 10 units/Kg/Hr infusion rate, and adjusted as directed below.

Table 3. Heparin Total Dose (units) in ESRD subjects on hemodialysis or combined hemodialysis hemoperfusion.

	After 2 weeks optimized heparinization on hemodialysis alone	After 2 weeks optimized heparinization on hemodialysis plus hemoperfusion	After 5 weeks optimized heparinization on hemodialysis plus hemoperfusion
Mean Total heparin for 4 hour session (n=6)	7145.83	7629.17	8062.50
SD	3626.00	2802.21	3113.95
Units/Kg	100.53	107.33	113.42

Table 4. Heparin Initial Bolus Dose (units) and Hourly Infusion Rates in same ESRD subjects in Table (above) on hemodialysis.

	Heparin schedule after 2 weeks

	optimized heparinization on hemodialysis alone	
	Bolus Dose(units)	Infusion rate/Hr(units)
Subject 1	1000	300
Subject 2	5000	2200
Subject 3	4500	700
Subject 4	3000	1500
Subject 5	4000	1200
Subject 6	5000	2000
Mean	3750	1316.7
SD	1541.1	732.8
Average Wt (Kg)	71.00	
Units/Kg	52.8	10.3

A $T_{initial}$ blood sample will be collected from the arterial blood line to establish the aPTT baseline level. The loading dose of heparin (30 – 50 U/kg) will be administered into the venous line and the needle flushed with up to 10 ml of normal saline to ensure that the entire loading dose enters the circulation. The heparin will be allowed to circulate for 3-5 minutes. Anticoagulation with an aPTT of 60-80 or an ACT of ~ 160 seconds to 210 seconds of baseline must be established and documented before starting CytoSorb treatment. CytoSorb or CytoSorb//Dialysis combined treatment will then be initiated along with the constant infusion of heparin at the prescribed rate. After treatment has begun, blood samples will be taken from the arterial line (from port A1 in the CytoSorb only sessions (Figure 1) and from port A1 in the Combined Treatment Sessions (Figure 3) for the aPTT measurement. The heparin infusion rate (U/hr) must be recorded on the appropriate CRFs. During the first two hours of treatment, the heparin infusion rate will be adjusted to maintain aPTT measurements of 60-80 seconds or ACT of ~ 160 seconds to 210 seconds of the baseline measurement.
Heparin infusion will be discontinued one hour before the end of treatment, unless otherwise directed by the investigator. An aPTT measurement must also be carried out 15 minutes prior to the end of the treatment to determine the subject's anticoagulation status (ensure that aPTT is returning to baseline).

3.3.3.2 Regional Citrate Anticoagulation

Regional Citrate Anticoagulation (RCA) varies from institution to institution. Please follow your own hospital procedures. An ionized serum calcium level taken between the point of citrate administration the inlet of the device should be established (<0.4mmol/l suggested) and documented before starting CytoSorb Treatment

3.3.4 Subjects Using Catheters for Vascular Access

In subjects using catheters for vascular access, where either urokinase or heparin is used as the "locking solution" to keep the catheter patent, the following steps should be performed prior to blood sampling and heparinization:

Using an aseptic procedure:

Step 1: Connect a syringe to the arterial port and aspirate up to 5 ml of blood and locking solution, clamp the line, remove the syringe and discard the blood and the syringe.

Step 2: Replace the syringe with a syringe to draw the lab specimens, open the clamp and aspirate the desired amount of blood for the laboratory tests. Close the clamp.

Step 3: Remove the syringe and attach the arterial bloodline. Fill the appropriate blood tubes with the blood that was drawn.

Step 4: Follow Step 1 for the venous port.

Step 5: Connect the syringe with the prescribed heparin to the venous port. Open the clamp. Inject the heparin. Clamp.

Step 6: Flush the venous catheter port with up to 10 ml of saline to insure that the entire heparin bolus has been injected into the subject

Step 7: Wait 3 to 5 minutes prior to initiating treatment, to allow the heparin to circulate and thus have the subject's blood anticoagulated as it reaches the extracorporeal circuit.

Step 8: Proceed with the initiation of CytoSorb or combined treatment according to the unit protocol.

3.3.5 Archive Samples

Archive samples will be collected at day 1-7 treatment sessions whether CytoSorb is used alone or combined with hemodialysis and every 7 days out to day 28 post treatment . Archive samples will be drawn from the A1 port into the appropriate collection tubes, centrifuged immediately, after which the serum/plasma will be separated into multiple serum/plasma storage tubes. Once aliquoted, the tubes may be maintained at -20 ºC (standard freezer for up to 5 days) before transfer to a -70 ºC freezer. Archive samples will be collected from all subjects at all study sites. These samples will be the subject of future testing for cytokines, drugs and hormones. Samples may be archived up to one year.

3.3.6 Complement Activation Studies (Sites as specified)

Changes in complement (C3a and sC5b-9) levels will be assessed for specified sites. Complement concentrations will be measured at two time points in a given session (T_0 & T_{30}), and will be assessed during hemoperfusion session day 3 only. T_0 blood samples will be drawn from the catheter access before administration of saline or heparin. T_{30} samples can be drawn from the arterial line of the blood set. Upon collection, samples will be immediately submitted for analysis or centrifuged immediately and stored at -70 ºC, and batch analyzed.

3.3.7 Arterial Blood Gases

Arterial blood gases should be collected daily for the first 7 days then every four days as defined by the CRF's. These samples should be collected at 0800 ± 1 hr via arterial stick.

3.3.8 Thrombus Formation

Throughout the treatment session, the clinical staff must monitor the CytoSorb device for blood leaks and clots. Any such adverse device events must be recorded on the appropriate CRFs.

3.3.9 HIT Antibody/D Dimer (Sites as specified)

Changes in HIT antibody and D Dimer levels will be assessed. HIT antibody and D Dimer concentrations will be assessed at least during hemoperfusion on days 1 and 7. If the subject

completes therapy prior to 7 days, the day treatment stops an additional sample for HIT antibody and D Dimer levels will be drawn. Blood samples will be drawn from the catheter access before administration of saline or heparin. Upon collection, samples will be immediately submitted for analysis or centrifuged immediately, stored at –70 ºC, and batch analyzed.

3.3.10 Immune System CD4 Cell Activation

Cell-mediated immunity and the responsiveness of the immune system will be assessed in both treated and control patients using an assay that examines the ability of CD-4 cells to become activated. This data will be correlated with cytokine reduction. Blood samples will be collected at T0 on days 1,3,5, 7,14 and on the day of discharge and sent for processing to the centralized laboratory at University of Goettingen. Samples must be assayed within 30 hours of collection and can be kept at room temperature.

4 SUBJECT TRIAL INTERRUPTION, DISCONTINUATION OR EARLY DEPARTURE FROM HOSPITAL (< 28 DAYS)

4.1 Missed Scheduled Treatment

Investigator(s) must make every effort to ensure that all treatments are given. The investigator(s) or the study coordinator(s) must immediately notify the MedaSorb clinical monitor to discuss possible options when subjects miss a scheduled treatment.

A missed treatment session can be rescheduled.
Within treatment period, subjects will be disqualified and removed from the study if there is:

• More than one treatment session is missed

4.2 Subject Discontinuation

Participation in the study may be discontinued for any of the following reasons:

- Non-compliance with prescribed treatment
- Development of Severe adverse events
- Any condition which, in the investigator's medical judgment, should disqualify the subject
- Voluntary withdrawal
- Death

Any infection not related to the treatment but occurring during the study should be treated in the standard fashion, i.e. with antibiotics or other medications administered at the end of the combined treatment sessions (once the extracorporeal blood has been returned from the circuit). The investigator(s) or the study coordinator(s) must notify MedaSorb through their study assigned clinical monitor when a subject is discontinued from the trial. Subjects discontinued from the trial will not be allowed to re-enter the study.

Any subject not completing the entire study [the 7 day consecutive treatment period] will be replaced so that a minimum of 30 subjects will have complete data sets for their assigned group beyond the first 11 patients assigned to safety analysis. Data will be analyzed by "intention-to-treat" for subjects who failed to complete the study.

Subjects that do not complete the full 6 hour therapy can be treated with a second device. It will be up to the physician to determine if it is acceptable risk for the patient to receive additional therapy. The following represents a guideline for the clinician to consider:

Total Time of First Dose Therapy	Recommendation
0 - 4 hours	Use second device
4 - 6 hours	Discontinue Therapy

4.2.1 Severe Adverse Events (SAEs)

Investigators are required to comply with reporting requirements as required by local country law. (See investigator's Regulatory Binder). In this regard, Investigators shall submit to the MedaSorb's representative and to the reviewing Ethics Committee (EC) a report of any SAE occurring during this study as soon as possible, but in no event later than 10 working days after the investigator first learns of the effect. A thorough evaluation of all circumstances associated with any SAE effect or a subject's death will be made to determine if the device effect or death is related to the clinical trial. In the event of a subject's death, an autopsy should be conducted whenever possible. Investigators or designated representatives are asked to notify MedaSorb within 24 hours of learning of any SAE in order to facilitate a timely investigation of the incident. It will be the responsibility of MedPass to notify the appropriate agencies as required by German law.

4.3 Trial Discontinuation

This trial may be discontinued early for any one of the following reasons:

- Determination by the physician that the risk to subjects is unacceptable
- Failure of the investigator or staff to maintain an adequate number of subjects
- Failure of the investigator or staff or subjects to comply with the protocol
- Termination of the trial device's development by the Sponsor
- Unacceptable changes in personnel or facilities at the investigator's site
- Determination that no statistically significant result can be obtained
- Withdrawal of EC or Competent Authority approval

It is the investigator's responsibility to prepare the subjects for the trial's discontinuation when such a decision has been made. The investigator must inform the subjects in some manner about the results, arrange for continuation of their medical care, and collect the final data to be incorporated into the final study report. The investigator(s) must report any withdrawal of EC approval to MedaSorb within 5 working days of receiving such notice.

4.4 Early Departure from Hospital

In some cases the subjects may depart the hospital before the 28 day assessment. Under these circumstances the patient may have had adequate recovery and further hospital care is not warranted leading to discharge from the hospital prior to day 28. Alternatively, the patient may be discharged to hospice, etc. Under these circumstances the next available Post Treatment period CRF's should be completed including scheduled blood samples. For example, if the patient is scheduled to discharges on day 11 complete Post Treatment period day 14, if the patient is scheduled to discharges on day 16 complete Post Treatment period day 21, etc. Once the patient is discharged no further blood sample are required and only Day 60 follow-up concerning survival is required per the protocol.

5 DATA MANAGEMENT

5.1 Data Collection

All data to be reported and analyzed for the clinical study will be recorded using an electronic data capture (EDC) system for whom an IC Form is obtained, and no study treatment will be administered without a completed IC Form. Data will be directly entered into a Web-based electronic case report form (eCRF).

Data will be recorded on the appropriate CRFs for all study subjects for whom an IC Form is obtained, and no study treatment will be administered without a completed IC Form.

Data will be monitored to identify inconsistent or missing data and adverse device effects. Data problems will be addressed via data queries sent to the investigational sites and during site visits. All hard copy forms and data files will be secured to ensure confidentiality. Quality assurance procedures are designed to ensure that complete, accurate and timely data are submitted, that protocol requirements are followed, and that adverse device effects are reported as required.

Investigational sites will maintain all source documents for data as required by the protocol, e.g. laboratory results, treatment session flow sheets, supporting medical records, IC Form. The source documents will be used at the regular monitoring visits to verify information submitted on the case report forms.

5.2 Electronic Case Report Forms (eCRFs)

Appropriate training on eCRFs will be provided to all investigational sites by MedPass. Investigators and study site coordinators will be responsible for completion of the eCRFs in a timely manner. MedaSorb staff or representatives will monitor the eCRFs. Data will be recorded on the appropriate eCRFs and will include the following information.

At Screening

- Informed consent Form
- Inclusion / exclusion criteria
- Vital signs
- Medical history/co-morbid conditions/demographics
- Concomitant medications
- Laboratory results
- Chest X-Rays
- Weight (if available)

Treatment Sessions and Combined Treatment Sessions

- Vital signs
- Concomitant medications
- Line pressures – arterial & venous
- Laboratory results
- Ventilation parameters
- Fluid balance
- Concomitant medications
- Adverse event(s)
- Completion / discontinuation information

6 ANALYSIS PLAN

The proposed study is an open-label, two-arm, prospective randomized controlled trial with CytoSorb treatment versus standard of care in the treatment of ARDS/ALI. Up to a total of 100 subjects will be enrolled in order to collect complete data sets on 30 subjects that receive CytoSorb treatment and 30 control subjects that do not receive CytoSorb treatment, the control arm.

6.1 Endpoints

A large number of endpoints are planned for collection to give a broad assessment of the clinical utility of CytoSorb. Endpoints are separated into three levels (primary, secondary, exploratory) based upon their perceived relevance for an eventual pivotal trial for the demonstration of device safety and effectiveness. Data from subjects who receive Xigris after any treatment with CytoSorb will not be censored and will be included in data analysis.

Primary Endpoint:

Relative IL-6 levels as a percent (%) of baseline will be lower in subjects receiving CytoSorb treatment in conjunction with the standard of care as compared to control subjects receiving only the standard of care for ARDS/ALI in the setting of sepsis

Secondary Endpoints:

- Ventilation Support: This is defined as the number of days, between day 1 and day 28 where the subject was:
  - off ventilator (ventilator free days) or
  - $FiO_2 \leq 40\%$ and PEEP ≤ 5 cm H_20
- TNF-α, IL-1b, IL-10, CRP
- 28-day all cause mortality
- Oxygen Index (OI)
- P/F ratios
- MODS scores

Exploratory Endpoints:

- SOFA Score
- APACHE II score
- 60 day all cause mortality
- Vasopressor use
- Immune system CD4 cell activation

6.2 Sample Size

This is an efficacy study to gather data on the impact of CytoSorb treatment on cytokine removal. MedaSorb will enroll up to 100 subjects in order to accrue complete data sets on 30 subjects. All subjects will be screened and selected based on the study inclusion and exclusion criteria

6.3 Study Population

This is an open label, two-arm, prospective randomized study. Subjects will be randomized in a 1:1 ratio in blocks of 4 until either of the following are met: either 30 treatment and 30 control subjects have completed their 28-day follow-up, or 100 subjects have been enrolled. Falling short of the 30/30 target due to subject deaths or other loss to follow-up will not be considered a protocol deviation.

To preserve random assignment of subjects to treatment arms, each subject will be assigned a double-blinded treatment code at randomization time that will be unblinded by the investigator after subject randomization.

6.3.1 Standard Statistical Methods

The analysis of this study will take place in three distinct steps:

First, comparisons between the prospective treatment groups will be performed (~30 treatment vs. ~30 control); on an intention to treat basis. This will be referred to as the "prospective comparison". Unless otherwise stated, all p-values will be considered significant at a two-sided significance level of 0.05. Two-sided p-values less than 0.10 will be considered evidence of a trend.

Second, subgroup analysis will be performed, in order to evaluate the impact on clinical results of potential predictive factors; patient age, patients receiving renal replacement therapy (RRT) and Vasopressor use.

Third, as per protocol, analysis will be performed according to the recommendations of the DSMB. All patients who present at least one major deviation will be dropped for the second analysis.

Summary statistics (mean, median, standard deviation, range), including histograms, will be generated for all relevant variables according to their characteristics (continuous or categorical). Chi-Square testing will be used to allow comparisons of categorical variables. In the comparison of continuous variables, if standard parametric techniques are found to be inadequate (Student's test), an appropriate non-parametric technique (i.e. Rank-Sum Test) will be used. All analyses will be conducted using SAS® version 9.1 or higher. All matched comparison testing will take the paired nature of the data into account.

6.3.2 Demographic and Baseline Screening Characterization

Demographic variables and baseline screening data will be summarized.

6.3.3 Analysis of Study Endpoints[1]

6.3.3.1 Prospective Comparison

Sufficient number of treated subjects such that at least 30 (expected) complete the 28 days of follow-up, parametric methods will be available from which to draw rough inference about the performance of the treatment relative to the 30 controls. Statistical methods listed in 6.3.1 will be used to facilitate all of the following comparisons:

[1] Ventilator-free days (VFD) will include the worst-case scenario (VFD=0) as one of several sensitivity analyses that will be performed. LVCF for mortality analysis will not be used. Every effort will be made to record the mortality status of each enrolled subject at the 28 and 60-day time point.

- Statistically significant clearance of cytokine and intra-session reduction in CytoSorb treated subjects vs untreated control subjects

- All secondary and exploratory endpoints will be assessed between study groups via appropriate statistical methodology.

- Correlation analyses of Cytokine concentrations vs. secondary and exploratory endpoints will be presented as appropriate.

6.3.4 Safety Analysis

The safety of CytoSorb will be evaluated on both clinical and device based parameters. Clinical parameters will include:

- Incidence of adverse events
- Clinical status
- Vital signs
- Clinical laboratory changes
- aPTT
- Hematologic parameters
- Line pressures
- Concomitant medications
- Additional laboratory tests

Device specific safety parameters will include:

- Incidence of any technical or device related issues that may adversely affect the subject or the hemoperfusion procedure

All subjects who receive treatment will be included in the safety evaluation. All adverse events will be listed and the frequencies, severity, and relationship to the treatment procedure will be tabulated.

6.4 Study Duration

The study is expected to last approximately 22 months in total duration. First subject treatment is projected to be April 2009 with last subject treatment December 2009 (22 months of enrollment duration period). Each subject will receive up to 7 days of therapy in hospital. The subject will be monitored in the hospital for 28 days or discharge which ever comes first, with a determination of patient survival at 60 days via questionnaire form the initial hospitalization date of enrollment into the study". The following table outlines the Study Timelines:

	First Visit	Last Visit
First Subject	April, 2008	June, 2008
Last Subject	December, 2009	January, 2010
Final Report	⟶	April , 2010

6.5 Data Safety Monitoring Board

An independent Data Safety Monitoring Board (DSMB) will, at regular intervals, assess the results with respect to patient safety.

Composition: The Data Safety Monitoring Board (DSMB) is composed of at least four members (two physicians from the fields of Nephrology, one physician experienced with ARDS and one biostatistician), who are independent of the conduct of the trial. The DSMB will review the study at least every 6 months from the date of first enrollment or the first 10 patients (10 treatment and 10 control).

The DSMB can meet as it feels appropriate, but it is envisaged that it will review safety tables and conduct an interim analysis as soon as 10 patients have reached their 28-days follow-up. Access to this documentation is not to be granted to any non-member while the study is ongoing.

Responsibilities: The DSMB will be informed of all Serious Adverse Events. They will review safety data for the trial based on interim analysis of adverse events, protocol deviations and device malfunction. They will also be responsible for recommending any changes to the trial or stop the trial due to safety concerns. The DSMB may call a meeting **at any time** if there is reason to suspect that safety is an issue.

7 ETHICS/ ETHICAL REQUIREMENTS

Declaration of Helsinki (Appendix 3)

The study will be performed in accordance with ISO/EN 14155, part 1 and 2, recommendations guiding physicians in biomedical research involving human s adopted by the 18th World Medical Assembly, Helsinki, Finland (1964 and later revisions), ICH and US FDA GCP guidelines.

It is the responsibility of the investigator to obtain approval of the study protocol from the competent Ethics Committee and to keep the Ethics Committee informed of any serious adverse events, serious adverse device effects, and amendments to the protocol. All correspondence with the Ethics Committee should be filed by the investigator and copies sent to the CRO and the Sponsor.

7.1 Ethics Committee

The respective Ethics Committee (EC) shall review, approve, require modifications or disapprove the investigational plan for any medical device investigation.

7.2 Ethical Conduct of the Study

The study will be conducted in accordance with the International Conference of Harmonization (ICH) Good Clinical Practices (GCP) Guidelines as well as local laws.

7.3 Privacy Policy and getting Informed Consent, process

It is the responsibility of the investigator to give each subject full and adequate verbal and written information regarding the objective and procedure of the study and the possible risks involved and to obtain signed informed consent from all subjects prior to inclusion in the study unless the subject's health condition does not allow informed consent, in which case the national procedures will be applied.

Septic patients with ALI/ARDS may benefit most from Hemofiltration therapy, if the therapy is initiated early in the course of sepsis and ALI/ARDS. There is a relatively small time-window of opportunity for patients to be included in this study, since subjects are only eligible for participation in the study, if ALI/ ARDS was diagnosed within the last 72 hrs and if subjects were on ventilation for < 3 days prior to enrollment (see inclusion criteria on pg. 8). The consenting process has to take this situation into account.

Informed consent will be obtained by either one of three modes:

a) Through the patient directly

Patients who are capable of giving their consent are briefed orally and in writing before the study. Patients will give their consent in writing on the "Patient informed consent form" (See, Appendix 1).

b) Through a legal healthcare proxy

Due to the design of the study most of the patients eligible will not be capable of giving their consent because they are on ventilation. Thus, it will not be possible to obtain the patient's oral or written declaration of consent before the start of the study. In cases where a legal healthcare proxy has been appointed by the national procedures (a German court) prior to enrollment into the study, written informed consent of the patient’s legal healthcare proxy will be obtained.

c) Through approval from a third party physician and the *designated* legal healthcare proxy

It is expected that in most cases neither the patient will be capable of giving his informed consent, nor a legal healthcare proxy has been appointed prior to study enrollment. In these cases the national procedures for appointing a healthcare proxy (family member, relative or near friend) will be immediately initiated. Because this process may take longer than the time-window for study enrollment and the patient thus may loose the opportunity to benefit from the additional hemofiltration therapy, it seems justified, in view of the

urgency of the therapy, to install a process which will allow to start the study before the designated healthcare proxy has been officially appointed by a German court.

In these cases the study will be started after obtaining

i) written informed consent from the designated legal healthcare proxy for whom the appropriate national procedures have been initiated

AND

ii) written approval from an independent third party physician.

The third party approval process requires a physician who is not involved in the study and who does not belong to the medical unit conducting the study or to the team caring for the patient. The third party physician will be required to make a determination that the proposed therapy is in the best interest of the patient. The Independent Physician must justify his decision professionally in writing, using the Independent Physician Consent Form (See appendix I, last part)

The original signed consent form(s) is/are filed with the subject study records, and a copy is provided to the subject. Attempts will be made to inform those patients who are not initially capable of giving their consent about the clinical trial and obtain their consent at a later date, provided this is permitted by the patient's health condition during the hospital stay. If the patient should decide, at a later date, that he does not wish to take part in the study, he may, up until the end of the study (closure of the database), arrange for his data to be deleted and his blood samples to be destroyed via the investigator.

7.4 Subject Data Protection

The subjects will be identified in the CRFs with unique subject number and initials.

The subject must be informed that the data will be stored and analyzed by computer, that national regulations for handling of computerized data will be followed, and that only the investigator and the Sponsor or representatives of the Sponsor will have access to individual subject data. Furthermore, the subjects should be informed about the possibility of inspection of relevant parts of the hospital records by the Sponsor or Health Authorities.

Prior to screening for the study, all subjects will be informed in detail about the nature of the clinical investigation, as well as its risks, potential benefits, and any anticipated discomforts.

8 CLINICAL MONITORING

8.1 Clinical Monitoring Procedures

Clinical monitors will be assigned to this study based upon the geographical location of the sites. The clinical monitors are qualified by their training and experience to oversee the conduct of the study. The clinical monitors' responsibilities include maintaining regular contact with each investigational site, through telephone contacts, correspondence and on-site visits to ensure that:

- Investigational sites are qualified to participate in the study
- The protocol is followed
- Timely and accurate data are submitted
- Problems with inconsistent and incomplete data are resolved
- Severe adverse device effects are reported to the clinical monitor in a timely manner
- The site facilities continue to be adequate
- Investigational device records are maintained, and
- All required reports are submitted to the EC and MedaSorb in a timely manner.

Clinical monitoring questions should be addressed to the designated clinical monitor. The clinical monitor in charge of this clinical trial is:

MedPass International
95 bis Boulevard. Pereire
75017 Paris, France
Tel : 33 01 42 12 83 30
Fax : 33 01 40 53 81 11

A clinical monitor will conduct periodic clinical monitoring visits to each investigational site. Prior to the start of data collection for a given site, the site and personnel to be involved in the study will be visited. The objectives of this site initiation visit are to:

- Confirm that the investigator and study personnel fully understand the protocol, the data collection procedures and the requirements to be met before starting the study

- Confirm that the investigator and study personnel fully understand the procedures related to the selection of subjects for this study

- Confirm that the investigator and study personnel have appropriate knowledge, experience and equipment to comply with the study requirements.

The site initiation visit will be documented in a report. To ensure that investigators and their staff understand and accept their defined responsibilities, the clinical monitors will maintain regular communication and perform periodic site monitoring visits during the course of the study to verify the continued acceptability of the facilities, compliance with the investigational plan and relevant CA regulations, and maintenance of complete records. Clinical monitoring will include review and resolution of missing, erroneous, or inconsistent results and source document checks (i.e., comparison of submitted study results to original reports) to assure the accuracy of the reported data.

The clinical monitor will evaluate and summarize the results of each site visit in written reports, identifying and reporting data problems with any investigator and specifying recommendations for resolution of noted deficiencies.

8.1.1 Data Quality Assurance

Standard Case Report Forms (CRFs) will be provided for use at all investigational sites. Investigators are responsible for completion and timely submission of the forms for data processing.

Data processing will be done by MedaSorb or assigned CRO.

Quality assurance procedures are designed to ensure that complete, accurate and timely data are submitted, that protocol requirements are followed, and that complications, adverse events and adverse device effects are correctly reported.

Incoming data are reviewed to identify inconsistent or missing data and adverse events. Data problems will be addressed with the principal investigator. All hard copy forms and data files will be secured to ensure confidentiality.

At the conclusion of the study, the clinical monitor will ensure that:

- All CRFs are accurate and complete, and have been submitted to data management
- All laboratory reports are on file
- All information contained in the Regulatory Binder is current
- All information contained in the Source Documents Binder is current
- All adverse events have been reported and if appropriate, resolved
- A final accountability of unused devices will be conducted and any remaining devices returned to the Sponsor

8.1.2 Anticipated Adverse Events

Adverse events that are generally associated with hemodialysis and hemoadsorption may include but are not limited to:

- Events that may be associated with hemoperfusion, including: complement activation and slight reduction in formed elements of blood, hypervolemia, including hypertension, hypotension, headache, nausea/vomiting, muscle cramping, chills, fever, bleeding, heparin induced thrombocytopenia (HIT), arrhythmia/palpitations, dyspnea, agitation, itching, and a feeling of coldness.

- Clinical events that may be associated with blood pump equipment (blood pump, dialyzer, bloodlines, and CytoSorb) malfunction or defect, or procedural error may include: blood loss, blood clotting, hemolysis, and air embolism.

9 INVESTIGATOR RESPONSIBILITIES

9.1 Investigator Records

The investigator will maintain the following complete, accurate and current study records in the site Regulatory Binder:

- Correspondence with CA, MedaSorb or its representative and the EC
- Records of the receipt of the devices and CytoSorb device log sheets
- CytoSorb Instructions for Use (IFU)
- Forms and supporting documents, as appropriate
- Current study protocol and all amendments, as appropriate
- Adverse event reports
- EC approval for the protocol, the ICD, and HIPAA Authorization Form
- Signed Investigator Agreement with CVs of the investigators and all participating sub investigators
- Screening log sheets
- Telephone and e-mail log sheets
- Signature log sheets
- Monitoring log sheets

All study records shall be maintained for a period of 2 years after the latter of the following two dates: the date on which the investigation is terminated or completed.

9.2 Investigator Reports

9.2.1 Severe Adverse Events (SAEs)

Investigators are required to comply with reporting requirements as required by local country law. (See investigator's Regulatory Binder). In this regard, Investigators shall submit to the MedaSorb's representative and to the reviewing Ethics Committee (EC) a report of any SAE occurring during this study as soon as possible, but in no event later than 10 working days after the investigator first learns of the effect. A thorough evaluation of all circumstances associated with any SAE effect or a subject's death will be made to determine if the device effect or death is related to the clinical trial. In the event of a subject's death, an autopsy should be conducted whenever possible. Investigators or designated representatives are asked to notify MedaSorb within 24 hours of learning of any SAE in order to facilitate a timely investigation of the incident. It will the responsibility of MedPass to notify the appropriate agencies as required by German law.

9.2.2 Withdrawal of Ethics Committee Approval

The investigator(s) shall report to MedaSorb's representative within five (5) working days if, for any reason, the EC withdraws approval to conduct the investigation. The report will include a complete description of the reason(s) for which approval was withdrawn.

9.2.3 Deviation from the Investigational Plan

The investigator(s) shall notify MedaSorb's representative and the reviewing EC of any changes in, or deviations from, the protocol to protect the life or physical well being of the subject in an emergency. Such notice shall be given as soon as possible, but in no event later than five (5) working days after the emergency occurs. Except in such emergency, prior approval by the Sponsor is required for changes in or deviations from the study protocol. EC approval are required if these changes or deviations may affect the scientific soundness of the study, or the rights, safety or welfare of the subjects.

9.2.4 Use of the CytoSorb Device Without Informed Consent

No subject may be treated with CytoSorb prior to obtaining written informed consent (IC). If the investigator treats a subject with CytoSorb without prior IC, the investigator must report such use to MedaSorb's representative and the reviewing EC within five (5) working days after the use occurs.

9.2.6 Final Report

The investigator will submit a final report to MedaSorb's representative and to the EC and or CA as required by law within three (3) months of the termination of the study or termination of the investigator's participation in the study.

9.2.7 Other Reports

Upon request, the investigator shall provide accurate, complete and current information regarding any aspect of the study to MedaSorb, CA or EC. Beyond these regulatory requirements, full protocol compliance is vital to the success of the study, thus minimizing the number of cases later classified as incomplete, unusable, or not evaluable.

9.2.8 Publishing of Data

All information related to the study is confidential. No information shall be released to the public domain without prior authorization from MedaSorb. Since this study is multi-centered, all investigators are encouraged to consider joint publications. Publications (abstract or complete article) related to the study must be submitted for review by MedaSorb 60 days before planned submission to a journal or society, and fifteen (15) days for review before any poster presentation, abstract or other written or oral material which describes the results of the study, in accordance with the written investigator agreement.

9.2.9 Investigator Materials Accountability

The investigator will be provided with the investigational devices after a complete regulatory package has been submitted to MedaSorb's representative. This package will include the following:

- EC approval (Informed Consent Form)
- Signed study protocol
- Signed current CVs for the investigators and all sub investigators
- Signed Investigator Agreement
- Financial Disclosure Information
- Current investigator medical license

The investigator will be responsible for maintaining accurate CytoSorb device accountability records. These records will be reviewed during monitoring visits to ensure compliance. At the end of the study, any unused devices will be returned to MedaSorb's representative.

10 SPONSOR RESPONSIBILITIES

10.1 Beginning the Study

MedaSorb will not begin the clinical investigation or part of the investigation until EC approval and requirements of the country specific Competent Authority (CA) have been met .

10.2 Selecting Investigators

MedaSorb will select investigator(s) and staff who are qualified by training and experience to investigate the devices.

10.3 Control of Investigational Devices

MedaSorb's representative MedaSorb will ship investigational devices only to qualified clinical sites and investigator(s) participating in the study.

10.4 Obtaining Agreements

MedaSorb's representative will obtain a signed Investigator Agreement including:

- The investigator's relevant experience (e.g. including dates, location and type of experience, explanation of circumstances leading to termination of studies previously undertaken by the investigator (if applicable)
- Statement that the investigator is committed to conduct the investigation in accordance with the agreement, the study protocol, other applicable CA regulations and other conditions imposed by the EC/CA
- Agreement to supervise all testing of the device involving human subjects, and
- A statement to ensure that the requirements for obtaining informed consent are met.

10.5 Securing Compliance

MedaSorb's representative will ensure that the investigator continues to comply with the signed agreement, the investigational plan, other applicable CA regulations, and conditions imposed by the EC or CA. If the investigator demonstrates noncompliance, MedaSorb will discontinue shipments of the device to the investigator, terminate the investigator's participation in the investigation and require the investigator to return any unused devices to the Sponsor, unless this action would jeopardize the rights, safety or welfare of a subject.

10.6 Severe Adverse Events (SAEs)

MedaSorb will conduct an evaluation of any severe adverse device events and report the result of such evaluation to the Authorized Representative who will in turn notify the EC and CA as required by country law.

10.7 Withdrawal of EC/CA approval

MedaSorb’s reprehensive shall comply with the requirements of each country with regard to reporting to EC and CA requirements for dissemination of information relating to withdrawal of approval.

10.8 Current Investigator List

MedaSorb’s representative shall comply with local country laws as it relates to a current list of the names and addresses of all investigators participating in the study.

10.9 Progress Reports

MedaSorb will submit periodic progress reports to CA’s and EC’s as required by local country law.

10.10 Other Reports

MedaSorb’s representative will submit a copy of any report to required authorities from an investigator for use of the device without ICD within five (5) working days of such notice to MedaSorb Technologies and appropriate country authorities as required by law.

11 REFERENCES

Fisch, B.J. and Spiegel, D.M. Assessment of excess fluid distribution in chronic hemodialysis subjects using bioimpedance spectroscopy. Kidney Int; 49(4):1105-1109, 1996 (Abstract only).

Hoenich, N.A. and Levin, N.W. Can technology solve the clinical problem of “dry weight”? Nephrol Dial Transplant; 18:647-650, 2003.

Hoenich, N.A. and Stamp, S. Clinical Performance of a New High-Flux Synthetic Membrane. Am J Kidney Diseases; 36(2): 345-352, 2000.

Ward, R.A. Heparinization for Routine Hemodialysis. Advances in Renal Replacement Therapy; 2(4):362-370, 1995.

12 APPPENDIX 1: SAMPLE INFORMED CONSENT

SAMPLE INFORMED CONSENT DOCUMENT

German Translation
PATIENTENINFORMATION UND EINVERSTÄNDNISERKLÄRUNG FÜR STUDIENTEILNEHMER

Multizentrische und randomisierte Wirksamkeitsstudie zum Hämoperfusionsgerät CytoSorb™ der Firma MedaSorb zur adjunktiven Therapie bei Patienten mit akuter respiratorischer Insuffizienz *(Acute Lung Injury, ALI*; *Acute Respiratory Distress Syndrome,* ARDS) bei bestehender Sepsis

INSTITUTION NAME:
INSTITUTION ADRESSE:
PRÜFARZT/-ÄRZTIN:
VOR- UND ZUNAME
STUDIENTEILNEHMER/IN:

Die vorliegende Einverständniserklärung enthält eventuell Begriffe, die Sie nicht kennen. Bitten Sie Ihren Prüfarzt/Ihre Prüfärztin oder andere Mitarbeiter/innen der Studie, Ihnen alle Ausdrücke und Informationen, die Sie nicht vollständig verstehen, zu erklären. Auf diese Weise möchten wir sicherstellen, dass Sie die Studie und Ihre Rolle in der Studie ganz genau verstehen.

Bitte legen Sie eine Kopie dieser Patienteninformation zu Ihren Unterlagen.

EINLEITUNG:
Sie wurden eingeladen, an einer klinischen Studie zu einem in der Erforschung befindlichen Gerät teilzunehmen. Bevor Sie sich für die Teilnahme an der Studie entscheiden, müssen Sie die möglichen Risiken und den Nutzen der Teilnahme kennen. Diese Einverständniserklärung enthält Informationen über die Forschungsstudie. Eine Mitarbeiterin oder ein Mitarbeiter der Studie wird zur Verfügung stehen, um Ihre Fragen zu beantworten und Ihnen weitere Erklärungen zu geben. Wenn Sie sich mit der Teilnahme an der Studie einverstanden erklären möchten, müssen Sie diese Patienteninformation aufmerksam lesen und die Einverständniserklärung unterschreiben. Dieser Vorgang wird als „Patienteninformation und Einverständniserklärung" bezeichnet. Wenn Sie sich mit der Teilnahme einverstanden erklären, werden Sie nach dem Zufallsprinzip entweder einer Behandlungsgruppe, die mit CytoSorb behandelt wird, oder einer Kontrollgruppe, welche die Standardbehandlung erhalten wird, zugeordnet. Lesen Sie die folgenden Informationen sehr sorgfältig durch und stellen Sie den Mitarbeitern der Studie gerne alle Fragen, die Ihnen einfallen.

ZIEL DER STUDIE:
Sie wurden zur Teilnahme an dieser Studie eingeladen, da Sie an akuter respiratorischer Insuffizienz (ARDS/ALI) als Komplikation einer Sepsis leiden. Eine Sepsis ist eine schwerwiegende Krankheit, die durch eine Infektion des Blutes durch Bakterien ausgelöst wird. Aus verschiedenen Gründen (z. B. durch die Einnahme von Antibiotika) werden die Bakterien, welche die Infektion verursachen, in manchen Fällen nicht erkannt. Als Patientin oder Patient, die/der an ARDS/ALI leidet, können Sie nicht selbständig atmen und benötigen ein Beatmungsgerät, das Ihre Atmung unterstützt. Außerdem werden von Ihrem Arzt/Ihrer Ärztin für die Behandlung der Atemwegserkrankung eventuell Antibiotika und/oder andere Medikamente

verschrieben. Ihre Atembeschwerden sind zum Teil auf eine Entzündung der Lungen zurückzuführen. Diese Krankheit nennt sich systemisches inflammatorisches Response-Syndrom (SIRS), bei dem Ihr Körper Chemikalien (Zytokine) produziert, die Schockreaktionen und Organschäden verursachen können.

Prüfärzte am <NAME DES INSTITUTS EINFÜGEN> und MedaSorb Technologies Inc. (MedaSorb, 7 Deer Park Dr, Suite K, Monmouth Junction, NJ 08852, USA, Tel: #1 732-329-8885) führen eine Forschungsstudie zur Prüfung der Sicherheit und Wirksamkeit eines in der Erforschung befindlichen (experimentellen) Geräts mit dem Namen CytoSorb™ (im Folgenden CytoSorb) durch, das als ergänzende Behandlung zur Standardtherapie bei ARDS/ALI verwendet werden soll. CytoSorb ist ein Gerät, das Chemikalien wie z. B. Zytokine aus dem Blut entfernt. Eine Senkung der erhöhten Zytokinwerte im Blut könnte zu einer Verkürzung der Dauer der künstlichen Beatmung und einer Beschleunigung der Rekonvaleszenz führen. Derzeit verfügbare medikamentöse Therapien zielen nicht auf die Senkung von Zytokinen ab und bekämpfen somit nicht die Entzündung. Die Dauer der künstlichen Beatmung wird nicht verkürzt. Wenn Sie der Behandlungsgruppe zugeordnet werden, werden Sie bis zu sieben Tage lang bzw. bis zum Absetzen der künstlichen Beatmung (je nachdem, welches Ereignis zuerst eintritt) mit dem Gerät CytoSorb behandelt.

CytoSorb wurde zuvor bereits für eine andere Anwendung bei chronischen Nierenerkrankungen eingesetzt. Das Gerät wurde bei 21 Patienten im Rahmen von insgesamt 350 Behandlungen verwendet: Sechs Dialysepatienten erhielten über eine Zeitraum von fünfzehn Wochen drei Mal wöchentlich eine Behandlung mit dem CytoSorb.

Wenn Sie sich für die Teilnahme an dieser Studie entscheiden, werden Sie bis zu sieben Tage lang in Folge mit dem CytoSorb behandelt und anschließend 28 Tage lang im Krankenhaus nachbeobachtet. Nach Abschluss dieser Zeit endet auch Ihre Teilnahme an der Studie. Sechzig Tage nach der Behandlung findet eine Nachuntersuchung bei Ihrem Arzt/Ihrer Ärztin statt, um Ihre Fortschritte zu beurteilen. Die Aufnahme von bis zu Teilnehmern an bis zu fünfzehn Prüfzentren in Europa ist für die Studie geplant.

VERFAHREN:
Um an der Studie teilnehmen zu können, müssen Sie mindestens 18 und höchstens 80 Jahre alt sein.

Ihr Prüfarzt/Ihre Prüfärztin wird Ihre Krankengeschichte prüfen. Patienten mit instabilen Krankheiten (z. B. Krebs, einige Infektionskrankheiten oder ischämische Herzkrankheiten) dürfen nicht an der Studie teilnehmen. Ihr Prüfarzt/Ihre Prüfärztin werden Ihnen weitere Gründe, warum eine Teilnahme für Sie eventuell nicht in Frage kommt, erläutern.

Sie dürfen auch nicht an der Studie teilnehmen, wenn Sie schwanger sind. Aus diesem Grund wird vor der Aufnahme in die Studie bei allen Frauen im gebärfähigen Alter ein Schwangerschaftstest durchgeführt. Alle Teilnehmer an der Studie werden nach dem Zufallsprinzip entweder einer Kontroll- oder einer Behandlungsgruppe zugeordnet. Wenn Sie der Kontrollgruppe zugeordnet werden, erhalten Sie die Standardbehandlung, die Ihnen von Ihrem Arzt/Ihrer Ärztin verschrieben wurde. Außerdem werden während der ersten sieben Tage der Teilnahme an der Studie zusätzliche Blutproben entnommen. Wenn Sie der Behandlungsgruppe zugeordnet werden, erhalten Sie die von Ihrem Arzt/Ihrer Ärztin verordnete Standardbehandlung sowie zusätzlich eine Behandlung mit dem CytoSorb über einen Zeitraum von sieben Tagen. Bei der Behandlung mit dem CytoSorb wird Ihr Blut im Gerät gespült. Hierfür wird ein doppellumiger Dialysekatheter in eine Ader eingeführt. Die Behandlung mit dem CytoSorb wird

sieben Tage lang täglich über eine Zeitraum von sechs Stunden durchgeführt, bzw. bis die künstliche Beatmung abgesetzt wird, je nachdem, welches Ereignis zuerst eintritt. Für die Verwendung des CytoSorb ist außerdem Heparin, ein Medikament zur Blutverdünnung eine systemische, oder regionale extrakorporale Antikoagulation Verfahren erforderlich, , das verhindert, dass Ihr Blut im Gerät gerinnt. Die Blutproben werden gesammelt und ein Jahr lang von Ihrem Studienzentrum aufbewahrt. Diese Proben werden für die Analyse von Zytokinen und Hormonen weiterverwendet.

Sollten Sie für die Teilnahme an der Studie in Frage kommen werden Ihnen vielleicht schon vor Studienbeginn Blutproben entnommen um zu bestätigen, dass Sie auch wirklich alle erforderlichen Einschlusskriterien für die Teilnahme an der Studie erfüllen.
Vor, während und nach einigen Behandlungssitzungen der Studie werden Blutproben entnommen, um Ihre Zytokinwerte und andere Routineparameter in Ihrem Blut zu untersuchen.

Die Gesamtmenge Blut, die pro Tag entnommen wird, ist sehr gering (etwa vier Esslöffel voll) und dürfte keinen Einfluss auf Ihr Wohlergehen haben. Bei jeder Sitzung im Rahmen der Studie werden Informationen über Ihre Behandlung erhoben. Für die medizinischen Informationen wird Ihre Krankenakte geprüft. Sie werden zu anderen Medikamenten, die Sie eventuell einnehmen, sowie Ihrer allgemeinen Gesundheit befragt.

MÖGLICHE RISIKEN UND BEEINTRÄCHTIGUNGEN DURCH DIE BEHANDLUNG:
Die Anlage eines Katheters in eines Ihrer Blutgefäße geht mit einem leicht erhöhten Risiko der allgemeinen Behandlung bei ARDS/ALI einher. Die Risiken einer Hämoperfusion sind Blutungen oder Probleme durch Blutgerinnsel, niedriger Blutdruck, Kältegefühl und Infektionen. Eventuell bestehen durch die Teilnahme an dieser Studie zusätzliche Risiken im Vergleich zur Standardbehandlung. Hierzu gehören: allergische Reaktionen, die eventuell schwerwiegend oder lebensbedrohlich sein könnten, eine Verringerung der Anzahl an Thrombozyten, die zu einem erhöhten Blutungsrisiko führen kann, sowie eine eventuell erforderliche Anpassung Ihrer Medikation. Eventuell tritt auch ein vorübergehender Abfall von bestimmten Proteinen und Zellen in Ihrem Blut auf. Zu diesem Zeitpunkt liegen keine Anzeichen dafür vor, dass dies schädlich für Ihre Gesundheit sein könnte. Dennoch wird Ihr Blut täglich untersucht, um die genannten Risiken zu prüfen. Außerdem besteht die Möglichkeit, dass die Teilnahme an der Studie keinen medizinischen Nutzen für Sie bedeutet. Es werden alle Vorsichtsmaßnahmen ergriffen, um die Risiken für Sie zu minimieren.

ERWARTETER NUTZEN DURCH DIE BEHANDLUNG:

Wenn Sie sich für die Teilnahme an der Studie entscheiden, könnte Ihnen ein Nutzen dadurch entstehen, dass die Zytokinwerte in Ihrem Blut sinken, die Entzündung nachlässt und sich eventuell die Dauer der künstlichen Beatmung verkürzt. Die Studie wird weitere Informationen zur Sicherheit und Wirksamkeit von CytoSorb zur Verwendung bei Patienten in der Zukunft erbringen und das Verständnis der Behandlung von ARDS/ALI fördern.

KOSTEN:
Alle Verfahren und Behandlungen, die im Rahmen der Studie erforderlich werden und über die Standardbehandlung bei ARDS/ALI hinausgehen, werden Ihnen kostenlos zur Verfügung gestellt. Wenn Sie sich mit der Teilnahme an der Studie einverstanden erklären, erhalten Sie außerdem auch alle Tests und Materialien, die mit der Studie in Zusammenhang stehen, kostenlos.

.

BEZAHLUNG FÜR DIE TEILNAHME:
Für die Teilnahme an dieser Studie ist keine Bezahlung vorgesehen.

ALTERNATIVEN:
Die Studie wurde für die Prüfung der Sicherheit und Wirksamkeit von CytoSorb in Kombination mit der Standardtherapie bei ARDS/ALI durch Sepsis entwickelt. Wenn Sie sich gegen eine Teilnahme an der Studie entscheiden, erhalten Sie dennoch weiterhin die Standardbehandlung, die Ihnen von Ihrem Arzt/Ihrer Ärztin verschrieben wurde.

Falls während des Verlaufs der Forschungsstudie neue Erkenntnisse bekannt werden, wird die Behandlung eventuell geändert. In jedem Fall können Sie jedoch selbst entscheiden, ob Sie unter diesen Umständen weiter an der Studie teilnehmen möchten oder nicht.

VERSICHERUNG
Für alle Studienteilnehmer wird eine Versicherung abgeschlossen, die für jegliche Beeinträchtigung durch Gesundheitsschäden aufkommt, die auf die Teilnahme an der Studie zurückgeführt werden können. Während des Verlaufs der klinischen Studie dürfen die Studienteilnehmer nur nach Einverständnis des jeweiligen Prüfarztes/der jeweiligen Prüfärztin eine andere Behandlung in Anspruch nehmen, es sei denn, es handelt sich um eine Notfallsituation. Die klinische Prüfärztin/der klinische Prüfarzt muss über jegliche Notfallbehandlung unverzüglich Bericht erstatten. Wenn Sie glauben, dass Ihre Gesundheit durch die Teilnahme an der Studie beeinträchtigt wurde, müssen Sie dies umgehend Ihrer Versicherung mitteilen. Andernfalls verlieren Sie eventuell Ihren Versicherungsanspruch. Sie müssen Ihre Versicherung entweder persönlich benachrichtigen oder der Einfachheit halber Ihrem Prüfarzt/Ihrer Prüfärztin Bescheid sagen, die/der anschließend in Ihrem Namen Ihre Versicherung benachrichtigt.
Die Versicherung wurde mit Allianz (Allianz Global Corporate & Speciality, Königinstrasse 28, 80802 München) abgeschlossen. Die Versicherungspolice mit der Nummer: **IHA 70/0445/0107591/509** wird Ihnen zusammen mit diesem Schreiben ausgehändigt.
.

FREIWILLIGE TEILNAHME/VORZEITIGER ABBRUCH:
Ihre Teilnahme an dieser Studie ist freiwillig. Sie können die Teilnahme an der Studie jederzeit ablehnen oder die Studie abbrechen, ohne dass Sie hierfür auf Vorteile verzichten müssten, auf die Sie ansonsten einen Anspruch hätten. Ihre Entscheidung hat auch keinerlei Einfluss auf Ihre Behandlung im jeweiligen Klinikum in der Zukunft.

Der Prüfarzt/die Prüfärztin oder der Sponsor der Studie kann Ihre Teilnahme an der Studie außerdem jederzeit aus folgenden Gründen ohne Ihr Einverständnis beenden:

- Wenn das für die Studie verwendete Gerät schädlich für Ihre Gesundheit sein könnte
- Wenn Sie die Kriterien für die Studie nicht erfüllen
- Wenn Sie mehr als eine Behandlungssitzung verpassen
- Wenn die Studie insgesamt abgebrochen wird

Wenn Ihre Teilnahme an der Studie vorzeitig beendet wird, werden Sie gebeten, eine abschließende Blutprobe für einige Tests abzugeben. Sie müssen Ihren Prüfarzt/Ihre Prüfärztin benachrichtigen, falls Sie sich entscheiden, die Studie abzubrechen.
Sie werden über alle neuen Informationen im Zusammenhang mit der klinischen

Forschungsstudie informiert, die dazu führen könnten, dass Sie Ihre Meinung über die Teilnahme ändern könnten.

FRAGEN:
Sie können jederzeit Fragen zu den Verfahren, zur Forschungsstudie selbst, zum Verfahren der Patienteninformation und Einverständniserklärung sowie zu Ihren Rechten als Studienteilnehmer stellen. Wenn Sie der Meinung sind, dass bei Ihnen eine Nebenwirkung oder andere ungewöhnliche Symptome auftreten oder wenn Sie sich während der Studie nicht wohl fühlen, wenden Sie sich bitte an <ANSPRECHPARTNER EINTRAGEN> oder andere Mitarbeiter der Studie unter:

<NAME, ADRESSE UND TELEFONNUMMER DER INSTITUTION>

Abends und an Wochenenden wenden Sie sich bitte an <ENTSPRECHENDE KONTAKTINFORMATIONEN EINTRAGEN>.

Im Fall aufkommender Probleme oder Fragen, die die Studie an sich betreffen, oder bei Fragen bezüglich Ihrer Rechte als Teilnehmer an einer klinischen Forschungsstudie können Sie sich an die Ethikkommission der medizinischen Einrichtung Freiburger Ethikkommission International wenden, Telefon: **0761-32007**. Bei der Ethikkommission handelt es sich um eine Organisation, die sämtliche Forschungsstudien einer Prüfung unterzieht, bevor die Aufnahme von Patienten beginnen kann.

VERTRAULICHKEIT UND GENEHMIGUNG
Ihre Teilnahme an dieser Studie wird so vertraulich gehandhabt wie im Rahmen der gesetzlichen Vorschriften möglich. Durch Ihre Teilnahme an dieser Studie erlauben Sie dem Prüfarzt (**<investigator/Prüfarzt, incl. „**Dr.**“**>), Ihre persönlichen, Ihre Gesundheit betreffenden Daten an die für die Studie zuständige Ethikkommission und an MedaSorb Technologies Inc. weiterzugeben, einschließlich Personen, die die Studie im Auftrag von MedaSorb Technologies Inc. durchführen. Ihre Daten können auch von Gesundheitsbehörden in Ihrem Land und in anderen Ländern sowie von der US-amerikanischen Lebens- und Arzneimittelbehörde FDA (Food and Drug Administration) eingesehen werden.

Die Angaben über Sie enthalten Informationen, anhand derer Sie identifiziert werden könnten – Name, Anschrift, Telefon, Fotos, Geburtsdatum, neue und bereits vorhandene Krankenakten sowie die Art verschiedener Tests und Verfahren, deren Datum und Ergebnisse. Die Angaben können Daten aus Ihrer Krankenakte und Daten enthalten, die im Rahmen der Studie erstellt oder erfasst wurden, und zwar entweder elektronisch oder in gedruckter Form.

Durch Ihre Teilnahme an dieser Studie genehmigen Sie die Freigabe von Daten, die nicht vom Prüfarzt, sondern von anderen medizinischen Versorgungsstellen erbrachte Nachsorgeuntersuchungen betreffen. Der Zugriff auf Ihre persönlichen gesundheitsbezüglichen Daten beschränkt sich auf die Erfassung und Verarbeitung von Daten, die zum Abschluss dieser Studie erforderlich sind. Wenn Ergebnisse von Studien wie dieser in medizinischen Fachzeitschriften oder auf Fachtagungen veröffentlicht werden, werden teilnehmende Patienten nicht namentlich identifiziert. Diese Studie wird unter Beachtung aller den Schutz der Privatsphäre betreffenden Gesetze des jeweiligen Landes durchgeführt.

MedaSorb Technologies Inc. kann Ihre Daten auch in Forschungsdatenbanken einfügen, um bessere Sicherheits- und Leistungsmaßnahmen und andere Therapieansätze für Patienten zu

erforschen, Krankheitserkenntnisse zu vertiefen oder die Effizienz künftiger Studien zu verbessern.

Sie haben das Recht, Ihre diese Studie betreffenden Daten einzusehen und zu kopieren; wenden Sie sich zu diesem Zweck an die medizinische Einrichtung (**<name of institution/Name der med. Einrichtung**>), solange die Daten vom Prüfarzt (**<investigator/Prüfarzt, incl.** „Dr.“>) bzw. von der medizinischen Einrichtung aufbewahrt werden.

Multizentrische und randomisierte Wirksamkeitsstudie zum Hämoperfusionsgerät CytoSorb™ der Firma MedaSorb zur adjunktiven Therapie bei Patienten mit akuter respiratorischer Insuffizienz (*Acute Lung Injury, ALI; Acute Respiratory Distress Syndrome, ARDS)* bei bestehender Sepsis

Einverständniserklärung für gesetzlichen Betreuer

Name des Patienten/der Patientin: ________________________________
Adresse: ____

Ich habe die Informationen in dieser Einverständniserklärung gelesen und verstanden. Ich hatte die Gelegenheit, Fragen zu stellen. Die alternativen Behandlungsmethoden wurden mir erklärt und ich habe alle Antworten zur Studie erhalten, die ich brauchte. Ich habe verstanden, dass meine Teilnahme an der Studie freiwillig ist und dass ich die Forschungsstudie jederzeit abbrechen kann, ohne dass meine zukünftige Behandlung an dieser Klinik davon beeinflusst würde.

Ich erkläre mich freiwillig mit der Teilnahme an der Studie einverstanden.
Ich wurde über den bestehenden Versicherungsschutz für die Studie informiert. Ich habe eine Kopie der Versicherungsbedingungen erhalten.

Ich habe verstanden, dass ich eine Kopie der unterzeichneten und datierten Einverständniserklärung erhalten werde.
Ich erkläre zugleich, dass ich mit der im Rahmen der klinischen Prüfung erfolgenden Aufzeichnung von Gesundheitsdaten und mit der Einsichtnahme zu Prüfungszwecken durch Beauftragte des Auftraggebers oder der zuständigen Behörde einverstanden bin

Durch meine Unterschrift unter diese Einverständniserklärung verzichte ich auf keinerlei Rechte, auf die ich ansonsten als Teilnehmer an einer Forschungsstudie Anspruch hätte.

____
Name Teilnehmer/in (in Druckbuchstaben)

____ ______________
Unterschrift Studienteilnehmer/in Datum

____
Name der durch die Einverständniserklärung führenden Person
(in Druckbuchstaben)

____ ______________
Unterschrift der durch die Einverständniserklärung führenden Person Datum

____
Name Prüfarzt/-ärztin (in Druckbuchstaben, falls abweichend von oben)

____

____ ___________________

Unterschrift Prüfarzt/-ärztin Datum

Multizentrische und randomisierte Wirksamkeitsstudie zum Hämoperfusionsgerät CytoSorb™ der Firma MedaSorb zur adjunktiven Therapie bei Patienten mit akuter respiratorischer Insuffizienz (*Acute Lung Injury, ALI; Acute Respiratory Distress Syndrome, ARDS)* bei bestehender Sepsis

Einverständniserklärung für Patienten

Name, Vorname, Geburtsdatum **des Patienten/der Patientin:**

____

Ich,
Name, Vorname, Geburtsdatum des gesetzlichen **Betreuers des Patienten/der Patientin:**

____

bin per Beschluss des Amtgerichts
________________________vom___________________
(Sitz des Amtsgerichts) (Datum)

zum gesetzlichen Betreuer des oben genannten Patienten/Patientin bestellt worden.

Im Namen des Patienten stimme ich folgendem zu:
Ich habe die Informationen in dieser Einverständniserklärung gelesen und verstanden. Ich hatte die Gelegenheit, Fragen zu stellen. Die alternativen Behandlungsmethoden wurden mir erklärt und ich habe alle Antworten zur Studie erhalten, die ich brauchte. Ich habe verstanden, dass meine Teilnahme an der Studie freiwillig ist und dass ich die Forschungsstudie jederzeit abbrechen kann, ohne dass meine zukünftige Behandlung an dieser Klinik davon beeinflusst würde.

Ich erkläre mich freiwillig mit der Teilnahme an der Studie einverstanden.
Ich wurde über den bestehenden Versicherungsschutz für die Studie informiert. Ich habe eine Kopie der Versicherungsbedingungen erhalten.

Ich habe verstanden, dass ich eine Kopie der unterzeichneten und datierten Einverständniserklärung erhalten werde.
Ich erkläre zugleich, dass ich mit der im Rahmen der klinischen Prüfung erfolgenden Aufzeichnung von Gesundheitsdaten und mit der Einsichtnahme zu Prüfungszwecken durch Beauftragte des Auftraggebers oder der zuständigen Behörde einverstanden bin

Durch meine Unterschrift unter diese Einverständniserklärung verzichte ich auf keinerlei Rechte, auf die ich ansonsten als Teilnehmer an einer Forschungsstudie Anspruch hätte.

____

Name des Betreuers (in Druckbuchstaben)

Unterschrift Betreuer in Gesundheitsfragen Datum

Name Prüfarzt/-ärztin (in Druckbuchstaben)

Unterschrift Prüfarzt/-ärztin Datum

EINVERSTÄNDNISERKLÄRUNG EINES UNABHÄNGIGEN ARZTES ÜBER DIE STUDIENTEILNAHME DES PATIENTEN/IN

Multizentrische und randomisierte Wirksamkeitsstudie zum Hämoperfusionsgerät CytoSorb™ der Firma MedaSorb zur adjunktiven Therapie bei Patienten mit akuter respiratorischer Insuffizienz *(Acute Lung Injury ALI*; *Acute Respiratory Distress Syndrome,* ARDS) bei bestehender Sepsis

INSTITUTION NAME**:**

INSTITUTION ADRESSE:

PRÜFARZT/-ÄRZTIN:

Name, Vorname, Geburtsdatum **des Patienten/der Patientin:**

____
_

Adresse: ____

Ich bin damit einverstanden den Patienten/in in die Studie einzuschließen, weil ich glaube, damit in seinem Sinne zu handeln.

Um das Leben des Kranken zu retten, seine Gesundheit wiederherzustellen oder sein Leiden zu erleichtern, ist eine Behandlung ohne Aufschub erforderlich.

Der Patient wurde von mir darauf untersucht, ob er in der Lage ist, Wesen, Bedeutung und Tragweite seines Handelns sowie auch der klinischen Prüfung zu erkennen und seinen Willen hiernach zu bestimmen. Die Untersuchung hat ergeben, dass der/die Patient/in nicht einwilligungsfähig ist.

Die Einbeziehung in die Studie stellt dem/der Patient/in in seiner/ihrer akuten Situation einen möglichen persönlichen Behandlungsvorteil in Aussicht.

Ich erkläre, dass

- es aufgrund des engen therapeutischen Zeitraumes vor Beginn der Studie nicht möglich war, vor Gericht einen Betreuer als gesetzlichen Vertreter einsetzen zu lassen;

- ich den Inhalt der vorliegenden Patienteninformation (Version 3.1-20 Mar, 08) verstanden habe und der Teilnahme des Patienten an dem Forschungsprojekt und den Maßnahmen zum Datenschutz zustimme.
- mir kein mutmaßlicher Wille des Patienten bekannt ist, der der Teilnahme an der Studie widersprechen würde.

- ich weder an der Studie beteiligt bin, nicht zum Betreuungsteam des Patienten gehöre noch der studiendurchführenden Abteilung angehöre.

____

Name des unabhängigen Arztes
(in Druckbuchstaben)

____ _______________

Unterschrift des unabhängigen Arztes Ort,Datum

____

Name Prüfarzt/-ärztin (in Druckbuchstaben, falls abweichend von oben)

____ _______________

Unterschrift Prüfarzt/-ärztin Datum

13 APPENDIX II - HELSINKI DECLARATION (LATEST VERSION)

WORLD MEDICAL ASSOCIATION DECLARATION OF HELSINKI
Ethical Principles for Medical Research Involving Human Subjects

Adopted by the 18th WMA General Assembly, Helsinki, Finland, June 1964, and amended by the
29th WMA General Assembly, Tokyo, Japan, October 1975
35th WMA General Assembly, Venice, Italy, October 1983
41st WMA General Assembly, Hong Kong, September 1989
48th WMA General Assembly, Somerset West, Republic of South Africa, October 1996
and the 52nd WMA General Assembly, Edinburgh, Scotland, October 2000
Note of Clarification on Paragraph 29 added by the WMA General Assembly, Washington 2002
Note of Clarification on Paragraph 30 added by the WMA General Assembly, Tokyo 2004

A. INTRODUCTION

1. The World Medical Association has developed the Declaration of Helsinki as a statement of ethical principles to provide guidance to physicians and other participants in medical research involving human subjects. Medical research involving human subjects includes research on identifiable human material or identifiable data.

2. It is the duty of the physician to promote and safeguard the health of the people. The physician's knowledge and conscience are dedicated to the fulfillment of this duty.

3. The Declaration of Geneva of the World Medical Association binds the physician with the words, "The health of my patient will be my first consideration," and the International Code of Medical Ethics declares that, "A physician shall act only in the patient's interest when providing medical care which might have the effect of weakening the physical and mental condition of the patient."

4. Medical progress is based on research which ultimately must rest in part on experimentation involving human subjects.

5. In medical research on human subjects, considerations related to the well-being of the human subject should take precedence over the interests of science and society.

6. The primary purpose of medical research involving human subjects is to improve prophylactic, diagnostic and therapeutic procedures and the understanding of the etiology and pathogenesis of disease. Even the best proven prophylactic, diagnostic, and therapeutic methods must continuously be challenged through research for their effectiveness, efficiency, accessibility and quality.

7. In current medical practice and in medical research, most prophylactic, diagnostic and therapeutic procedures involve risks and burdens.

8. Medical research is subject to ethical standards that promote respect for all human beings and protect their health and rights. Some research populations are vulnerable and need special protection. The particular needs of the economically and medically disadvantaged must be

recognized. Special attention is also required for those who cannot give or refuse consent for themselves, for those who may be subject to giving consent under duress, for those who will not benefit personally from the research and for those for whom the research is combined with care.

9. Research Investigators should be aware of the ethical, legal and regulatory requirements for research on human subjects in their own countries as well as applicable international requirements. No national ethical, legal or regulatory requirement should be allowed to reduce or eliminate any of the protections for human subjects set forth in this Declaration.

B. BASIC PRINCIPLES FOR ALL MEDICAL RESEARCH

10. It is the duty of the physician in medical research to protect the life, health, privacy, and dignity of the human subject.

11. Medical research involving human subjects must conform to generally accepted scientific principles, be based on a thorough knowledge of the scientific literature, other relevant sources of information, and on adequate laboratory and, where appropriate, animal experimentation.

12. Appropriate caution must be exercised in the conduct of research which may affect the environment, and the welfare of animals used for research must be respected.

13. The design and performance of each experimental procedure involving human subjects should be clearly formulated in an experimental protocol. This protocol should be submitted for consideration, comment, guidance, and where appropriate, approval to a specially appointed ethical review committee, which must be independent of the investigator, the sponsor or any other kind of undue influence. This independent committee should be in conformity with the laws and regulations of the country in which the research experiment is performed. The committee has the right to monitor ongoing trials. The researcher has the obligation to provide monitoring information to the committee, especially any serious adverse events. The researcher should also submit to the committee, for review, information regarding funding, sponsors, institutional affiliations, other potential conflicts of interest and incentives for subjects.

14. The research protocol should always contain a statement of the ethical considerations involved and should indicate that there is compliance with the principles enunciated in this Declaration.

15. Medical research involving human subjects should be conducted only by scientifically qualified persons and under the supervision of a clinically competent medical person. The responsibility for the human subject must always rest with a medically qualified person and never rest on the subject of the research, even though the subject has given consent.

16. Every medical research project involving human subjects should be preceded by careful assessment of predictable risks and burdens in comparison with foreseeable benefits to the subject or to others. This does not preclude the participation of healthy volunteers in medical research. The design of all studies should be publicly available.

17. Physicians should abstain from engaging in research projects involving human subjects unless they are confident that the risks involved have been adequately assessed and can be satisfactorily managed. Physicians should cease any investigation if the risks are found to

outweigh the potential benefits or if there is conclusive proof of positive and beneficial results.

18. Medical research involving human subjects should only be conducted if the importance of the objective outweighs the inherent risks and burdens to the subject. This is especially important when the human subjects are healthy volunteers.

19. Medical research is only justified if there is a reasonable likelihood that the populations in which the research is carried out stand to benefit from the results of the research.

20. The subjects must be volunteers and informed participants in the research project.

21. The right of research subjects to safeguard their integrity must always be respected. Every precaution should be taken to respect the privacy of the subject, the confidentiality of the patient's information and to minimize the impact of the study on the subject's physical and mental integrity and on the personality of the subject.

22. In any research on human beings, each potential subject must be adequately informed of the aims, methods, sources of funding, any possible conflicts of interest, institutional affiliations of the researcher, the anticipated benefits and potential risks of the study and the discomfort it may entail. The subject should be informed of the right to abstain from participation in the study or to withdraw consent to participate at any time without reprisal. After ensuring that the subject has understood the information, the physician should then obtain the subject's freely-given informed consent, preferably in writing. If the consent cannot be obtained in writing, the non-written consent must be formally documented and witnessed.

23. When obtaining informed consent for the research project the physician should be particularly cautious if the subject is in a dependent relationship with the physician or may consent under duress. In that case the informed consent should be obtained by a well-informed physician who is not engaged in the investigation and who is completely independent of this relationship.

24. For a research subject who is legally incompetent, physically or mentally incapable of giving consent or is a legally incompetent minor, the investigator must obtain informed consent from the legally authorized representative in accordance with applicable law. These groups should not be included in research unless the research is necessary to promote the health of the population represented and this research cannot instead be performed on legally competent persons.

25. When a subject deemed legally incompetent, such as a minor child, is able to give assent to decisions about participation in research, the investigator must obtain that assent in addition to the consent of the legally authorized representative.

26. Research on individuals from whom it is not possible to obtain consent, including proxy or advance consent, should be done only if the physical/mental condition that prevents obtaining informed consent is a necessary characteristic of the research population. The specific reasons for involving research subjects with a condition that renders them unable to give informed consent should be stated in the experimental protocol for consideration and approval of the review committee. The protocol should state that consent to remain in the research should be obtained as soon as possible from the individual or a legally authorized surrogate.

27. Both authors and publishers have ethical obligations. In publication of the results of research, the investigators are obliged to preserve the accuracy of the results. Negative as well as positive results should be published or otherwise publicly available. Sources of funding, institutional affiliations and any possible conflicts of interest should be declared in the publication. Reports of experimentation not in accordance with the principles laid down in this Declaration should not be accepted for publication.

C. ADDITIONAL PRINCIPLES FOR MEDICAL RESEARCH COMBINED WITH MEDICAL CARE

28. The physician may combine medical research with medical care, only to the extent that the research is justified by its potential prophylactic, diagnostic or therapeutic value. When medical research is combined with medical care, additional standards apply to protect the patients who are research subjects.

29. The benefits, risks, burdens and effectiveness of a new method should be tested against those of the best current prophylactic, diagnostic, and therapeutic methods. This does not exclude the use of placebo, or no treatment, in studies where no proven prophylactic, diagnostic or therapeutic method exists.

30. At the conclusion of the study, every patient entered into the study should be assured of access to the best proven prophylactic, diagnostic and therapeutic methods identified by the study

31. The physician should fully inform the patient which aspects of the care are related to the research. The refusal of a patient to participate in a study must never interfere with the patient-physician relationship.

32. In the treatment of a patient, where proven prophylactic, diagnostic and therapeutic methods do not exist or have been ineffective, the physician, with informed consent from the patient, must be free to use unproven or new prophylactic, diagnostic and therapeutic measures, if in the physician's judgment it offers hope of saving life, re-establishing health or alleviating suffering. Where possible, these measures should be made the object of research, designed to evaluate their safety and efficacy. In all cases, new information should be recorded and, where appropriate, published. The other relevant guidelines of this Declaration should be followed.

Note: **Note of clarification on paragraph 29 of the WMA Declaration of Helsinki**

The WMA hereby reaffirms its position that extreme care must be taken in making use of a placebo-controlled trial and that in general this methodology should only be used in the absence of existing proven therapy. However, a placebo-controlled trial may be ethically acceptable, even if proven therapy is available, under the following circumstances:

- Where for compelling and scientifically sound methodological reasons its use is necessary to determine the efficacy or safety of a prophylactic, diagnostic or therapeutic method; or

- Where a prophylactic, diagnostic or therapeutic method is being investigated for a minor condition and the patients who receive placebo will not be subject to any additional risk of serious or irreversible harm.

All other provisions of the Declaration of Helsinki must be adhered to, especially the need for appropriate ethical and scientific review.

Note: **Note of clarification on paragraph 30 of the WMA Declaration of Helsinki**

The WMA hereby reaffirms its position that it is necessary during the study planning process to identify post-trial access by study participants to prophylactic, diagnostic and therapeutic procedures identified as beneficial in the study or access to other appropriate care. Post-trial access arrangements or other care must be described in the study protocol so the ethical review committee may consider such arrangements during its review.

The Declaration of Helsinki (Document 17.C) is an official policy document of the World Medical Association, the global representative body for physicians. It was first adopted in 1964 (Helsinki, Finland) and revised in 1975 (Tokyo, Japan), 1983 (Venice, Italy), 1989 (Hong Kong), 1996 (Somerset-West, South Africa) and 2000 (Edinburgh, Scotland). Note of clarification on Paragraph 29 added by the WMA General Assembly, Washington 2002.

14 APPENDIX III – LIST OF POTENTIAL INVESTIGATORS

1. **Prof. Dr. med. Jan Stange, M.D.**
Universität Rostock
Klinik für Innere Medizin/ Nephrologie
Abteilung Extracorporale Detoxikation
Ernst-Heydemann-Str. 6
18055 Rostock, Germany

2. **Michael Quintel, MD, PhD**
Georg-August-Universität Göttingen
37075 Goettingen, , Germany

3. **Prof. Dr. Konrad Reinhart**
Professor of Anesthesiology
University Hospital, Friedrich-Schiller-University
07745 Jena, Germany

4. **Dr. Christian Berhold**
Vivantes Klinikum im Friedrichshain
Landsberger Allee 49
10249 Berlin, Germany

5. **Dr. Glen Zachow**
Vivantes Humboldt Klinikum
Am Nordgraben 2
13509 Berlin, Germany

6. **Dr. Siegfried Veit**
Vivantes Klinikum im Friedrichshain
Landsberger Allee 49
10249 Berlin, Germany

7. **Prof. Dr. Helmut Schühlen**
Vivantes Auguste Viktoria Klinikum
Rubensstraße 125
12157 Berlin, Germany

8. **Dr. Lorenz Reill**
Vivantes Klinikum Neukölln
Rudower Str. 48
12351 Berlin, Germany

9. **Prof. Dr. H. Gerlach**
Vivantes Klinikum Neukölln
Rudower Str. 48
12351 Berlin, German

10. **Dr. Claudia Spies**
Dep. of Anesthesiology and Intensive Care Medicine
Charité Campus Mitte
Berlin, Germany, 10117

11. **Dr. Martin Tepe**
Dep. of Medicine IV
Charité Campus Benjamin Franklin
Berlin, Germany, 12203

12. **Dr. Simone Rosseau**
Dep.of Infectiology and Pneumonology
Charité Campus Mitte
Berlin, Germany, 10117

13. **Dr. Hoffman**
Dep. of Nephrology
Charité Berlin - Campus Virchow
Berlin, Germany,13353

14. **Dr. Mochmann**
Dep. of Nephrology
Charité Berlin - Campus Virchow
Berlin, Germany,13353

15. **Dr. Compton**
Dep. of Nephrology
Charité Berlin - Campus Virchow
Berlin, Germany,13353

16. **Dr. Claudia Spies/Dr. Susanne Niederberger**
Klinik für Anästhesiologie, operative Intensivmedizin
CC7 Charité-Universitätsmedizin - Virchow
Berlin Augustenburger Platz 1
13353 Berlin

17. **Dr. J. Brederlau**
Klinik und Poliklinik für Anästhesiologie
Universitätsklinikum Würzburg
Zentrum Operative Medizin
Oberdürrbacher Straße 6
97080 Würzburg, Germany

18. **Dr. R. Muellenbach**
Klinik und Poliklinik für Anästhesiologie
Universitätsklinikum Würzburg
Zentrum Operative Medizin
Oberdürrbacher Straße 6
97080 Würzburg, Germany

19. **Dr. M. Kredel**
Klinik und Poliklinik für Anästhesiologie
Universitätsklinikum Würzburg
Zentrum Operative Medizin
Oberdürrbacher Straße 6
97080 Würzburg, Germany

20. **Dr. Ralf Kuhlen**
Universitaetsklinikum Aachen
Pauwelsstraße 30
52074 Aachen, Germany

21. **Dr. Noeldge-Schomburg**
Klinik und Poliklinik für Anaesthesiologie und Intensivtherapy
Schillingallee 35
D-10857 Rostock, Germany

22. **Dr. Jochen Selbach**
Caritas Hospital Bad Mergentheim
Uhlandstraße 7
97980 Bad Mergentheim, Germany

23. **Dr. Rolf Roissant**
University of Aachen
Dept. of Anesthesiology, University Hospital Aachen,
Templergraben 55
Aachen, Germany 52062

24. **Dr. Norbert Weiler**
Department of Anesthesiology and Intensive Care Medicine
University Medical Center Schleswig-Holstein, Campus Kiel
Arnold-Heller-Straße 3
Kiel, Germany 24105

25. **Dr. Christian Putensen**
Department of Anaesthesiology and Intensive Care Medicine
University of Bonn
Sigmund-Freud-Strasse 25
Bonn, Germany 53105

26. **Dr. Andreas Meier-Helmann**
Department of Anesthesiology, Intensive Care Medicine HELIOS
Nordhäuser Straße 74
Erfurt, Germany 99089

27. **Dr. Gernot Marx**
Department of Surgical Intensive Care, University of Aachen, Pauwelsstrasse
Pauwelsstraße 30
Aachen, Germany 52074

15 APPENDIX IV – ARDSNET VENTILATION AND WEANING GUIDELINES

ARDSNet

NIH NHLBI ARDS Clinical Network
Mechanical Ventilation Protocol Summary
www.ardsnet.org

INCLUSION CRITERIA: Acute onset of
1. $PaO_2/FiO_2 \leq 300$ (corrected for altitude)
2. Bilateral (patchy, diffuse, or homogeneous) infiltrates consistent with pulmonary edema
3. No clinical evidence of left atrial hypertension

PART I: VENTILATOR SETUP AND ADJUSTMENT
1. Calculate predicted body weight (PBW)
   Males = 50 + 2.3 [height (inches) - 60]
   Females = 45.5 + 2.3 [height (inches) -60]
2. Select Assist Control Mode
3. Set initial TV to 8 ml/kg PBW
4. Reduce TV by 1 ml/kg at intervals ≤ 2 hours until TV = 6ml/kg PBW.
5. Set initial rate to approximate baseline VE (not > 35 bpm).
6. Adjust TV and RR to achieve pH and plateau pressure goals below.
7. Set inspiratory flow rate above patient demand (usually > 80L/min)

OXYGENATION GOAL: PaO_2 55-80 mmHg or SpO_2 88-95%
Use incremental FiO_2/PEEP combinations below to achieve goal

**FiO_2**	0.3	0.4	0.4	0.5	0.5	0.6	0.7	0.7
**PEEP**	5	5	8	8	10	10	10	12

**FiO_2**	0.7	0.8	0.9	0.9	0.9	1.0	1.0	1.0
**PEEP**	14	14	14	16	18	20	22	24

PLATEAU PRESSURE GOAL: ≤ 30 cm H_2O
Check Pplat (0.5 second inspiratory pause), SpO_2, Total RR, TV and pH (if available) at least q 4h and after each change in PEEP or TV.

If Pplat > 30 cm H_2O: decrease TV by 1 ml/kg steps (minimum = 4 ml/kg).
If Pplat < 25 cm H_2O: TV < 6 ml/kg, increase TV by 1 ml/kg until Pplat > 25 cm H_2O or TV = 6 ml/kg.
If Pplat < 30 and breath stacking occurs: may increase TV in 1 ml/kg increments (maximum = 8 ml/kg).

pH GOAL: 7.30-7.45
Acidosis Management: (pH < 7.30)
If pH 7.15-7.30: Increase RR until pH > 7.30 or $PaCO_2$ < 25 (Maximum RR = 35).
If RR = 35 and $PaCO_2$ < 25, may give $NaHCO_3$.
If pH < 7.15: Increase RR to 35.
If pH remains < 7.15 and $NaHCO_3$ considered or infused, TV may be increased in 1 ml/kg steps until pH > 7.15 (Pplat target may be exceeded).
Alkalosis Management: (pH > 7.45) Decrease vent rate if possible.

I:E RATIO GOAL: 1:1.0 - 1:3 Adjust flow rate to achieve goal.
If FiO_2 = 1.0 and PEEP = 24 cm H_2O, may adjust I:E to 1:1.

PART II: WEANING

A. Conduct a CPAP Trial daily when:
1. $FiO_2 \leq 0.50$ and PEEP ≤ 8.
2. PEEP and FiO_2 ≤ values of previous day.
3. Patient has acceptable spontaneous breathing efforts. (May decrease vent rate by 50% for 5 minutes to detect effort.)
4. Systolic BP ≥ 90 mmHg without vasopressor support.
   CONDUCTING THE TRIAL:
   Set CPAP = 5 cm H_2O, FiO_2 = 0.50
   If RR ≤ 35 for 5 min.: advance to Pressure Support Weaning below:
   If RR > 35 in < 5 min.: may repeat trial after appropriate intervention (e.g., suctioning, analgesia, anxiolysis)
   If CPAP trial not tolerated: return to previous A/C settings

B. PRESSURE SUPPORT (PS) WEANING PROCEDURE
1. Set PEEP = 5, and FiO_2 = 0.50
2. Set initial PS based on RR during CPAP trial:
   a. **If CPAP RR < 25:** set PS = 5 cm H_2O and go to step 3d.
   b. **If CPAP RR = 25-35:** set PS =20 cm H_2O then reduce by 5 cm H_2O at ≤ 5 min. intervals until RR = 26-35 then go to step 3a.
   c. **If initial PS not tolerated:** return to previous A/C settings.
3. **REDUCING PS:** (No reductions made after 1700 hors)
   a. Reduce PS by 5 cm H_2O q1-3 hr.
   b. If PS ≥ 10 cm H_2O not tolerated, return to previous A/C settings (Reinitiate last tolerated PS level next AM and go to step 3a)
   c. If PS = 5 cm H_2O not tolerated, return to PS = 10 cm H_2O. If tolerated, 5 or 10 cm H_2O may be used overnight with further attempts at weaning the next morning
   d. If PS = 5 cm H_2O tolerated for ≥ 2 hours assess for ability to sustain unassisted breathing below.

C. UNASSISTED BREATHING TRIAL:
1. Place on T-piece, trach collar, or CPAP ≤ 5 cm H_2O
2. Assess for tolerance as below for two hours.
   a. $SpO_2 \geq 90$: and/or $PaO_2 \geq 60$ mmHg
   b. Spontaneous TV ≥ 4 ml/kg PBW
   c. RR ≤ 35/min
   d. pH ≥ 7.3
   e. No respiratory distress (distress= 2 or more)
      - HR > 120% of baseline
      - Marked accessory muscle use
      - Abdominal paradox
      - Diaphoresis
      - Marked dyspnea
3. If tolerated consider extubation.
4. If not tolerated resume PS 5 cm H_2O.

COMPLETE PROTOCOL ONLINE: *www.ardsnet.org* or from *National Auxiliary Puplications Service (NAPS)*. To order 15 pages of supplementary material, contact NAPS, c/o Microfiche Publications, 248 Hempstead Tpk., West Hempstead, NY 11552 Document # 05542

© ARDS Network 2001

16 APPENDIX V – DATA SAFETY MONITORING BOARD MEMBERSHIP

Prof. Dr. med. Andreas Kribben – **DSMB Chairman**
Direktor der Klinik für Nieren- und Hochdruckkrankheiten
Universitätsklinikum Essen
Hufelandstr. 55
45122 Essen, Germany

Prof. Dr. med. Jan Galle
Direktor der Klinik für
Nephrologie und Dialyseverfahren
Klinikum Lüdenscheid
Paulmannshöher Strasse 14
D-58515 Lüdenscheid, Germany

Dr. Robert Bartlett
University of Michigan (Member of ARDSNet U.S.A)
1327 Jones Drive – Suite 201
Ann Arbor, MI 48105
U.S.A

Intentionally Left Blank
