# Supplementary material for: The effect of a novel extracorporeal cytokine hemoadsorption device on IL-6 elimination in septic patients: A randomized controlled trial
Source: PLoS One. 2017 Oct 30;12(10):e0187015. doi: 10.1371/journal.pone.0187015 (PMC5662220; doi:10.1371/journal.pone.0187015)
Supplement: S3 File — (DOCX) [file pone.0187015.s003.docx]

# S3 Inclusion and exclusion criteria

Patients were eligible for study inclusion when fulfilling all of the following criteria:

- Signed informed consent document
- Male or female with an age ≥ 18 and ≤ 80 years.
- Subjects must have diagnosis of ARDS or ALI, based on ARDSNet Definition [[1](#_ENREF_1)] established within the last 72 hours, confirmed by clinical, radiological, or physiologic findings
- Subject must be intubated
- ≤ 3 days on a ventilator prior to enrollment
- Subjects must have confirmed diagnosis of sepsis (according to the American College of Chest Physicians and Society of Critical Care Medicine criteria) [[2](#_ENREF_2)]
- Subject must have had at least 24 hours of antibiotic therapy
- Pre-menopausal female subjects must have negative pregnancy test
- Subject must be available for periodic blood sampling, study related assessments, and management at the treating institution for the duration of the study. Subject must have permanent home address to allow completion of 60 day follow-up
- Subject or health care proxy has the ability to understand and willingness to sign the informed consent form

The following exclusion criteria were applied:

- Currently participating in another clinical study involving investigational chemical compound, biologic, or device within the last 30 days prior to the start of this trial
- Neuromuscular disease that impairs the ability to ventilate spontaneously, such as C5 or higher spinal cord injury, amyotrophic lateral sclerosis, Guillain-Barré syndrome and myasthenia gravis
- Increased intracranial pressure, tricyclic antidepressant overdose, hemoglobin SS, hemoglobin SC or other conditions where hypercapnia would be contraindicated
- Severe chronic respiratory disease including hospitalization within last 6 months for respiratory failure
- Morbid obesity (Body Mass Index ≥40 kg/m2)
- Burns > 30% BSA, bone marrow transplant, lung transplant or end stage hepatic liver failure
- Subject with mean arterial pressure ≤ 60 mmHg and is not responsive to vassopressors
- Subject with active malignancy receiving chemotherapy or radiation treatment within last 60 days
- Subject with active malignancy receiving chemotherapy or radiation treatment within last 60 days
- Subjects with AIDS, CD4 count of < 200 or 14%, or the presence of an AIDS defining illness (HIV+ subjects may be enrolled)
- Subject with acute coronary syndrome
- Subjects with decompensated heart failure with New York Heart Association (NYHA) classification IV
- Subjects with Chronic Kidney Disease (CKD) stage 5 or greater will be excluded
- Subjects with end stage hepatic liver failure
- Subjects on immunosuppressive agents, excluding corticosteroids
- Platelets ≤ 20,000/mm3
- Subjects on anti-TNF therapy
- Subjects about to receive or receiving drotrecogin alpha (Xigris) therapy
- Subject is pregnant or breastfeeding
- Subject has a known allergy to any component of the CytoSorb hemoperfusion device
- Subject has any active disease condition that could limit compliance with the study procedure, including but not limited to the following: acute coronary syndrome, life-threatening cardiac arrhythmia, or psychiatric or social conditions, considered by investigator(s) to preclude successful completion of the study
