# Supplementary material for: The effect of a novel extracorporeal cytokine hemoadsorption device on IL-6 elimination in septic patients: A randomized controlled trial
Source: PLoS One. 2017 Oct 30;12(10):e0187015. doi: 10.1371/journal.pone.0187015 (PMC5662220; doi:10.1371/journal.pone.0187015)
Supplement: S4 File — (DOCX) [file pone.0187015.s004.docx]

# S4 Study sites

1. Department of Nephrology, Vivantes Klinikum im Friedrichshain, Berlin, Germany (n=18 patients)
2. Department of Internal Medicine – Cardiology, Vivantes Humboldt Klinikum, Berlin, Germany (n=5 patients)
3. Centre of Anaesthesiology, Emergency and Intensive Care Medicine, University Hospital Göttingen, Göttingen, Germany (n=29 patients)
4. Department of Anesthesiology and Intensive Care Medicine, HELIOS Klinikum, Erfurt, Germany (n=8 patients)
5. Anaesthesiology and Intensive Care Medicine, Campus Charité Mitte and Campus Charité Virchow-Klinikum, Charité - University Medicine Berlin, Berlin, Germany (n=3 patients)
6. Department of Anesthesiology and Intensive Care Medicine, HELIOS Klinikum, Erfurt, Germany (n=21 patients)
7. Department of Anesthesiology and Intensive Care Medicine, University of Bonn, Bonn, Germany (n=3 patients)
8. Department of Intensive Care and Intermediate Care, RWTH University Hospital Aachen, Aachen, Germany (n=4 patients)
9. Department of Anesthesiology and Intensive Care Medicine, University Medical Center Schleswig-Holstein, Campus Kiel, Kiel, Germany (n=8 patients)
10. Department of Nephrology and Medical Intensive Care, Charité University Hospital Campus Virchow-Klinikum, Berlin, Germany (n=1 patient)
