# Supplementary material for: The effect of a novel extracorporeal cytokine hemoadsorption device on IL-6 elimination in septic patients: A randomized controlled trial
Source: PLoS One. 2017 Oct 30;12(10):e0187015. doi: 10.1371/journal.pone.0187015 (PMC5662220; doi:10.1371/journal.pone.0187015)
Supplement: S1 Table — Values are given as median and interquartile ranges or as mean ± standard deviation unless otherwise noted. * Missing subscores on the Acute Physiologic and Chronic Health Evaluation (APACHE II Score) were counted as 0. Definition of abbreviations: MODS, multiple organ dysfunction score; P/F, arterial partial pressure of oxygen divided by inspired fraction of oxygen. (DOCX) [file pone.0187015.s005.docx]

**S1 Table. Demographic data and baseline characteristics of the study patients with available data for the primary endpoint.**

| Variable | Treatment group (n=36) | Control group (n=39) |  |
| --- | --- | --- | --- |
| Age [years] | 67 [56-74] | 66 [53-71] |  |
| Male gender, no. (%) | 25 (69.4%) | 29 (74.4%) |  |
| Weight [kg] | 75.8±14.0 | 83.6±17.6 |  |
| Height [cm] | 170.0±7.5 | 172.2±8.1 |  |
| Body mass index [kg/m^2^] | 26.2±4.8 | 28.1±5.3 |  |
| APACHE II score* | 24.6±4.9 | 23.0±5.8 |  |
| MODS | 10 [8-12] | 10 [7-12] |  |
| Renal replacement therapy, no. (%) | 14 (38.9%) | 7 (17.9%) |  |
| Diabetes mellitus, no. (%) | 15 (41.7%) | 15 (38.5%) |  |
| **Lung injury category** |  |  |  |
| Sepsis |  |  |  |
| Primary, no. (%) | 14 (38.9%) | 8 (20.5%) |  |
| Secondary, no. (%) | 22 (61.1%) | 31 (79.5%) |  |
| Trauma |  |  |  |
| Primary, no. (%) | 3 (8.8%) | 3 (7.9%) |  |
| Secondary, no. (%) | 0 (0%) | 0 (0%) |  |
| Aspiration |  |  |  |
| Primary, no. (%) | 3 (8.6%) | 5 (13.2%) |  |
| Secondary, no. (%) | 0 (0%) | 3 (7.9%) |  |
| Multiple transfusion |  |  |  |
| Primary, no. (%) | 0 (0%) | 2 (5.4%) |  |
| Secondary, no. (%) | 8 (22.2%) | 2 (5.4%) |  |
| Pneumonia |  |  |  |
| Primary, no. (%) | 18 (50%) | 17 (43.6%) |  |
| Secondary, no. (%) | 8 (22.2%) | 17 (43.6%) |  |
| Other |  |  |  |
| Primary, no. (%) | 3 (9.4%) | 10 (26.3%) |  |
| Secondary, no. (%) | 7 (21.9%) | 2 (5.3%) |  |
| Other comorbid conditions, no. (%) | 26 (72.2%) | 32 (84.2%) |  |

Values are given as median and interquartile ranges or as mean ± standard deviation unless otherwise noted.

* Missing subscores on the Acute Physiologic and Chronic Health Evaluation (APACHE II Score) were counted as 0.

Definition of abbreviations: MODS, multiple organ dysfunction score; P/F, arterial partial pressure of oxygen divided by inspired fraction of oxygen.
