# Supplementary material for: The effect of a novel extracorporeal cytokine hemoadsorption device on IL-6 elimination in septic patients: A randomized controlled trial
Source: PLoS One. 2017 Oct 30;12(10):e0187015. doi: 10.1371/journal.pone.0187015 (PMC5662220; doi:10.1371/journal.pone.0187015)
Supplement: S2 Table — Values are given as median and interquartile ranges. Definition of abbreviations: GOT, glutamic oxaloacetic transferase; GPT, glutamate pyruvate transferase; Gamma-GT, gamma glutamyl transpeptidase; LDH, lactate dehydrogenase. (DOCX) [file pone.0187015.s006.docx]

**S2 Table: Baseline hemodynamic variables, blood gas values and laboratory values of patients with available data for the primary endpoint.**

| Variable | Treatment group (n=36) | Control group (n=39) |
| --- | --- | --- |
| Heart rate [1/min] | 91.0 [83.0-105.0] | 90.0 [79.5-100.5] |
| Mean arterial blood pressure [mm Hg] | 71.0 [64.0-81.55] | 75.0 [66.5-87.5] |
| Diastolic arterial blood pressure [mm Hg] | 54.5 [46.8-60.5] | 59.0 [50.0-66.0] |
| Systolic arterial blood pressure [mm Hg] | 115.5 [95.0-124.5] | 119 [108.5-122.5] |
| Peripheral oxygen saturation [%] | 97.0 [96.0-98.0] | 95.0 [93.0-97.0] |
| Body temperature [°C] | 36.8 [35.9-37.3] | 37.3 [36.8-37.8] |
| Platelets [1/nl] | 125 [60-232] | 191 [134-319] |
| White blood cell count [1/µl] | 13.3 (8.7-17.8] | 17.3 (12.6-21.8] |
| Creatinine [mg/dl] | 1.7 [0.7-2.2] | 1.5 (1.0-2.8] |
| Urea [mg/dl] | 40.0 [21.0-81.0] | 56 [25.0-85.1] |
| Bilirubin [mg/dl] | 10.0 [5.5-19.1] | 9.7 [6.0-17.9] |
| Direct bilirubin [mg/dl] | 0.5 [0.2-0.9] | 0.3 [0.2-0.8] |
| Albumin [g/dl] | 1.7 [1.5-2.3] | 2.1 [1.9-2.2] |
| Sodium [mmol/l] | 142.0 [138.0-145.8] | 143.5 [138.0-147.0] |
| Potassium [mmol{l] | 4.3 [4.1-4.7] | 4.4 [4.2-4.7] |
| Calcium [mmol/l] | 1.9 [1.8-2.1] | 1.9 [1.8-2.0] |
| Chloride [mmol/l] | 107.0 [102.5-109.5] | 107.0 [104.0-112.0] |
| Phosphate [mmol/l] | 1.0 [0.8-1.3] | 1.2 [0.9-1.5] |
| Glucose [mg/dl] | 147.9 [120.0-173.0] | 133.3 [108.5-165.0] |
| GOT [U/l] | 43.0 [4.8-103.8] | 31.5 [3.2-95.5] |
| GPT [U/l] | 22.8 [4.0-63.0] | 25.0 [1.3-51.5] |
| Alkaline phopsphatase [U/l] | 55.0 [11.8-106.0] | 58.0 [4.0-79.0] |
| Gamma-GT [U/l] | 42.0 [12.7-130.0] | 31.0 [5.9-100.2] |
| LDH [U/l] | 225 [11.6-407.0] | 202.0 [10.3-421.0] |
| Total protein [g/l] | 4.3 [3.8-5.0] | 4.7 [4.2-5.1] |

Values are given as median and interquartile ranges.

Definition of abbreviations: GOT, glutamic oxaloacetic transferase; GPT, glutamate pyruvate transferase; Gamma-GT, gamma glutamyl transpeptidase; LDH, lactate dehydrogenase
