# Supplementary material for: The effect of a novel extracorporeal cytokine hemoadsorption device on IL-6 elimination in septic patients: A randomized controlled trial
Source: PLoS One. 2017 Oct 30;12(10):e0187015. doi: 10.1371/journal.pone.0187015 (PMC5662220; doi:10.1371/journal.pone.0187015)
Supplement: S3 Table — Values are given as median and interquartile ranges or as mean ± standard deviation unless otherwise noted. Definition of abbreviations: APRV, airway pressure release ventilation, HCO3, sodium bicarbonate; HFOV, high frequency oscillation ventilation; PaCO2, arterial partial pressure of carbon dioxide; PaO2, arterial partial pressure of oxygen; pbw, predicted body weight; PCV, pressure controlled ventilation; P-F-ration, arterial partial pressure of oxygen divided by inspired fraction of oxygen; PSV, pressure support ventilation. (DOCX) [file pone.0187015.s007.docx]

**S3 Table. Baseline mechanical ventilation therapy and blood gas values of patients with available data for the primary endpoint.**

| Variable | Treatment group (n=36) | Control group (n=39) |  |
| --- | --- | --- | --- |
| **Mode of mechanical ventilation** |  |  |  |
| PCV, no. (%) | 17 (47.2%) | 22 (56.4%) |  |
| VCV, no. (%) | 11 (30.6%) | 11 (28.2%) |  |
| PSV, no. (%) | 5 (13.9%) | 5 (12.8%) |  |
| APRV, no. (%) | 2 (5.6%) | 0 (0%) |  |
| HFOV, no. (%) | 0 (0%) | 1 (2.6%) |  |
| unknown, no. (%) | 1 (2.8%) | 0 (0%) |  |
| **Ventilator settings** |  |  |  |
| Total respiratory rate [1/min] | 22.3±5.4 | 21.5±5.6 |  |
| Inspired fraction of oxygen [%] | 57.2±17.7 | 54.3±20.3 |  |
| Positive endexpiratory pressure [cm H_2_O] | 12.5±3.2 | 11.5±3.6 |  |
| Pressure support level [cm H_2_O] | 15.9±7.5 | 17.5±6.9 |  |
| **Ventilator measurements** |  |  |  |
| Tidal volume [ml] | 457.0±109.5 | 498.2±111.8 |  |
| Tidal volume [ml/kg pbw] | 6.3±1.9 | 6.1±1.4 |  |
| Minute volume [l/min] | 10.1±2.2 | 10.3±2.9 |  |
| Mean airway pressure [cm H_2_O] | 17.9±4.1 | 17.1±4.4 |  |
| Plateau pressure [cm H_2_O] | 25.1±6.6 | 23.3±8.1 |  |
| **Blood gas values** |  |  |  |
| PF-ratio [mm Hg] | 162.5 [142.6-211.9] | 175.0 [149.7-233.6] |  |
| PaO2 [mm Hg] | 86.5 [75.6-107.2] | 85.0 [72.3-109.0] |  |
| PaCO2 [mm Hg] | 43.6 [37.8-52.5] | 42.2 [37.2-50.3] |  |
| Serum HCO3 [mmol/l] | 24.9 [22.0-27.1] | 25.7 [22.9-28.0] |  |
| Arterial pH | 7.37 [7.27-7.44] | 7.40 [7.27-7.45] |  |

Values are given as median and interquartile ranges or as mean ± standard deviation unless otherwise noted.

Definition of abbreviations: APRV, airway pressure release ventilation, HCO3, sodium bicarbonate; HFOV, high frequency oscillation ventilation; PaCO2, arterial partial pressure of carbon dioxide; PaO2, arterial partial pressure of oxygen; pbw, predicted body weight; PCV, pressure controlled ventilation; P-F-ration, arterial partial pressure of oxygen divided by inspired fraction of oxygen; PSV, pressure support ventilation.
