# Supplementary material for: The effect of a novel extracorporeal cytokine hemoadsorption device on IL-6 elimination in septic patients: A randomized controlled trial
Source: PLoS One. 2017 Oct 30;12(10):e0187015. doi: 10.1371/journal.pone.0187015 (PMC5662220; doi:10.1371/journal.pone.0187015)
Supplement: S4 Table — The effect of treatment for each variable was analyzed by a mixed model which was adjusted for day or day and timepoint if available. # values were log-transformed. Definition of abbreviations: GOT, glutamic oxaloacetic transferase; GPT, glutamate pyruvate transferase; Gamma-GT, gamma glutamyl transpeptidase; HCO3, sodium bicarbonate; LDH, lactate dehydrogenase; paCO2, arterial partial pressure of carbon dioxide; PaO2, arterial partial pressure of oxygen. (DOCX) [file pone.0187015.s008.docx]

**S4 Table. Analysis of the time course of vital parameters and laboratory values of patients with available data for the primary endpoint.**

| Variable | Coefficient | p-Value |  |
| --- | --- | --- | --- |
| Heart rate | 5.3746 | 0.054 |  |
| Systolic blood pressure | -0.9742 | 0.613 |  |
| Mean arterial pressure | -1.3141 | 0.633 |  |
| Diastolic blood pressure | -0.9742 | 0.613 |  |
| Body temperature | -0.3800 | **0.008** |  |
| Breaths per minute | 0.2017 | 0.879 |  |
| PaO_2_ | -6.3867 | 0.096 |  |
| PaCO_2_^#^ | -0.0244 | 0.527 |  |
| Arterial pH | 0.0090 | 0.614 |  |
| Serum HCO3 | 0.1521 | 0.815 |  |
| Platelets^#^ | -0.6161 | **0.003** |  |
| White blood cell count^#^ | -0.3165 | **0.005** |  |
| Creatinine | -0.2138 | 0.216 |  |
| Urea | -14.4210 | 0.091 |  |
| Bilirubine^#^ | -0.2340 | 0.332 |  |
| Direct bilirubine^#^ | -0.3081 | 0.314 |  |
| Albumin | -0.4461 | **<0.001** |  |
| Sodium | -1.4320 | 0.229 |  |
| Potassium | -0.0622 | 0.502 |  |
| Calcium | 0.0266 | 0.694 |  |
| Chloride | -0.6181 | 0.612 |  |
| Phosphate | -0.0675 | 0.207 |  |
| Glucose | 3.3200 | 0.592 |  |
| GOT^#^ | -0.1743 | 0.715 |  |
| GPT^#^ | -0.4800 | 0.335 |  |
| Alkaline phosphatase^#^ | 0.1167 | 0.775 |  |
| Gamma-GT^#^ | 0.0822 | 0.857 |  |
| LDH^#^ | 0.0402 | 0.924 |  |
| Total protein | -0.7102 | **<0.001** |  |

The effect of treatment for each variable was analyzed by a mixed model which was adjusted for day or day and timepoint if available.

^#^ values were log-transformed

Definition of abbreviations: GOT, glutamic oxaloacetic transferase; GPT, glutamate pyruvate transferase; Gamma-GT, gamma glutamyl transpeptidase; HCO3, sodium bicarbonate; LDH, lactate dehydrogenase; paCO2, arterial partial pressure of carbon dioxide; PaO2, arterial partial pressure of oxygen.
